# Templated Generation of a Bcl‐x_L_ Inhibitor by Isomer‐Free SPAAC Based on Azacyclonon‐5‐yne

**DOI:** 10.1002/chem.202202259

**Published:** 2022-10-01

**Authors:** Juliane Brauer, Marina Mötzing, Corinna Gröst, Ralf Hoffmann, Thorsten Berg

**Affiliations:** ^1^ Institute of Organic Chemistry Leipzig University Johannisallee 29 04103 Leipzig Germany; ^2^ Institute of Bioanalytical Chemistry and Center for Biotechnology and Biomedicine Leipzig University Deutscher Platz 5 04103 Leipzig Germany

**Keywords:** azides, cycloalkynes, inhibitors, protein–protein interactions, strain-promoted cycloadditions

## Abstract

High‐affinity inhibitors of large protein–protein interactions often have a high molecular weight, which compromises their cell permeability and oral bioavailability. We recently presented isomer‐free, strain‐promoted azide‐alkyne cycloaddition (iSPAAC) as a method by which to generate large, chemically uniform bioactive molecules inside living cells from two smaller components with higher cell permeability. Here, we present the synthesis of Fmoc‐protected azacyclonon‐5‐yne (Fmoc‐ACN) as the first cyclononyne suitable for iSPAAC. ACN facilitated the structure‐guided development of a single‐digit micromolar triazole inhibitor of the protein–protein interaction domain of the antiapoptotic protein Bcl‐x_L_. Inhibitor formation in aqueous buffer at 37 °C, templated by the target protein Bcl‐x_L_, proceeded 2800 times faster than the reaction between Fmoc‐ACN and benzyl azide under standard conditions in acetonitrile. Our data demonstrate the utility of cyclononynes for iSPAAC and their potential for achieving vastly accelerated templated reactions in aqueous environments.

## Graphical abstract

**Less strain, more gain**. We present Fmoc‐ACN as the first cyclononyne suitable for isomer‐free strain‐promoted azide–alkyne cycloaddition (iSPAAC). We demonstrate its use for the rational design and protein‐templated formation of a low‐micromolar inhibitor of protein–protein interactions mediated by Bcl‐x_L_. The lower intrinsic reactivity of cyclononynes as compared to cyclooctynes facilitated higher template effects and rate accelerations in the aqueous environment.

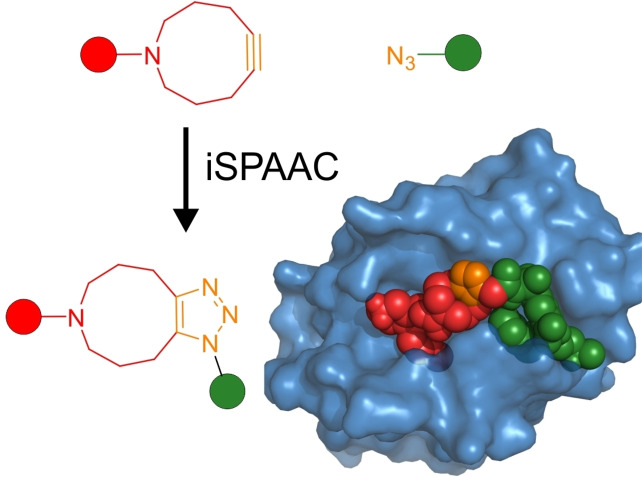

## Introduction

Protein–protein interactions are typically mediated by large and relatively featureless binding pockets.^[1]^ In consequence, high‐affinity inhibitors of protein–protein interactions often display a molecular weight which substantially exceeds the theoretical molecular weight maximum of 500 g mol^−1^, as defined by Lipinski's Rule of Five.^[2]^ This is likely to have negative effects on cell permeability and/or bioavailability, which poses a problem for inhibitors of intracellular protein–protein interactions. A general approach by which to overcome the molecular weight hurdle for large molecules targeting intracellular protein–protein interactions is to generate the molecules inside cells, using smaller building blocks with a higher cell permeability and/or bioavailability. Ideally, the reaction between the components would be sufficiently slow as to not already occur in the extracellular environment, but instead take place inside the cell through a templated reaction in the binding pocket of the biological target.

**Figure 1:**
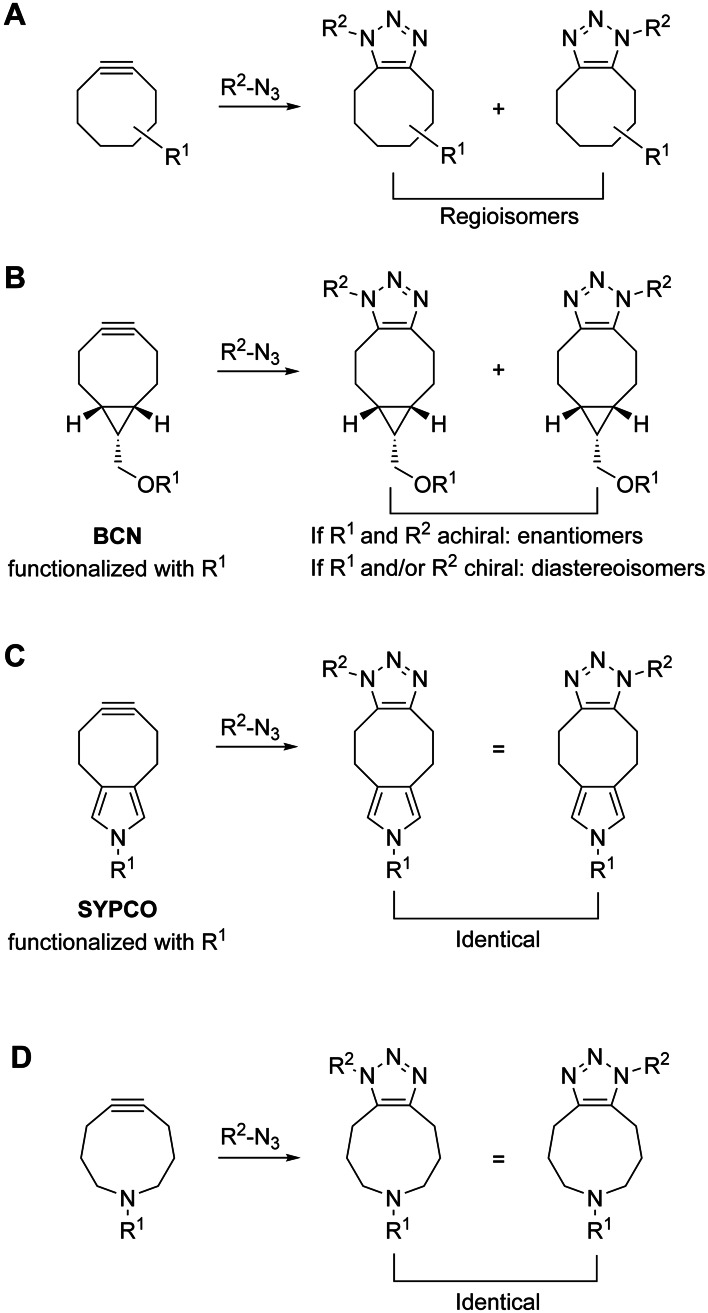
SPAAC with A) conventional cyclooctynes, B) BCN, C) the pyrrolocyclooctyne SYPCO, and D) symmetrical azacyclonon‐5‐ynes.

One possible reaction by which to achieve this goal is the strain‐promoted azide–alkyne cycloaddition using strained cycloalkynes,^[3]^ which has gained tremendous importance in recent years for labeling biomolecules.^[4]^ However, as the reaction between cycloalkynes and azides typically does not favor one of the two possible relative orientations, most functionalized cycloalkynes react in SPAAC to provide a mixture of regioisomers in a roughly equimolar ratio (Figure 1A).^[5]^ Some dibenzocyclooctynes use steric bulk added to one of the annelated benzene rings to favor one of the two possible regioisomers.^[6]^ However, dibenzocyclooctynes are too sterically demanding, even in the absence of additional steric bulk, to be useful for generating high‐affinity ligands of biomolecules. A notable exception to the formation of regioisomers in SPAAC is the bicyclononyne BCN,^[7]^ which generates stereoisomers when functionalized (Figure 1B). Because regio‐ and stereoisomeric organic molecules can have very different affinities for biomolecules, the use of isomeric mixtures for basic and applied research is limited. Chemical uniformity is advantageous when generating bioactive molecules in the biological environment.

We recently introduced the concept of isomer‐free SPAAC (iSPAAC).^[8]^ Key to the development of iSPAAC was the design and synthesis of the symmetrically substituted pyrrolocyclooctynes PYRROC,^[8]^ SYPCO,^[9]^ and TRIPCO,^[10]^ which do not form isomers in the reaction with azides, even when functionalized on their nitrogen atoms (Figure 1C). SYPCO was used to generate a triazole inhibitor of protein–protein interactions mediated by the anti‐apoptotic Bcl‐2 protein Bcl‐x_L._ ^[9]^ This study demonstrated that iSPAAC is a suitable method for the generation of bioactive molecules both in vitro and in cells.

A potential drawback of iSPAAC with pyrrolocyclooctynes is the formation of a tricyclic ring system (Figure 1C), the steric demands of which may impose a restriction on the biological targets that can be addressed. In addition, the high reactivity of pyrrolocyclooctynes may lead to premature product formation in the tissue culture medium before entering cells. Both problems can be addressed by the use of azacyclonon‐5‐yne or symmetrically substituted derivatives thereof, which do not form isomers in SPAAC when functionalized on nitrogen through an alkyl, amide, or carbamate bond (Figure 1D). In this context, we define isomers as molecules that can be interchanged into one another only by breaking and reestablishing of a covalent bond, whilst not regarding the interconverting rotamers of amides (*cis*/*trans*) or carbamates (*syn*/*anti*) as isomers. SPAAC with azacyclonon‐5‐ynes generates a bicyclic ring system, which needs less space in a protein‐binding pocket than the tricyclic system formed by iSPAAC with pyrrolocyclooctynes, and is thus more likely to be compatible with the binding pocket of a given biomolecule (Figure 1D). In addition, the reduced ring strain of cyclononynes as compared to cyclooctynes will reduce the unwanted background reaction outside cells. Overall, cyclononynes^[11]^ have received far less attention than cyclooctynes.

## Results and Discussion

**Figure 2:**
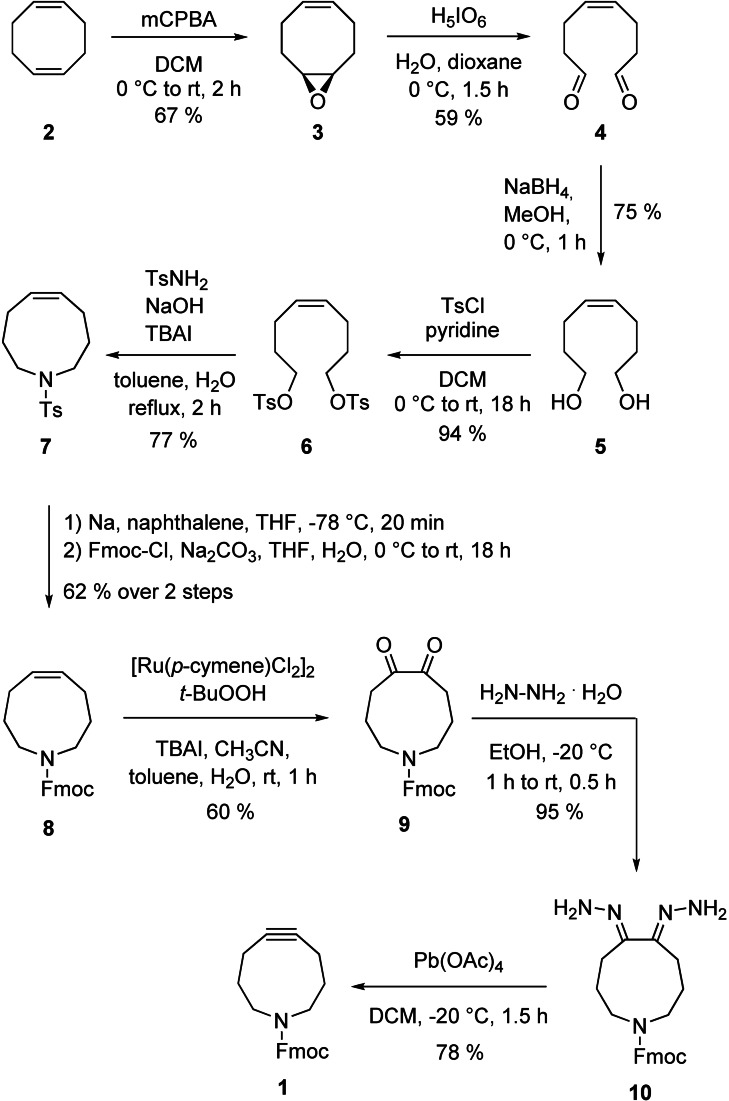
Synthesis of Fmoc‐ACN (**1**).

As a first step to explore the feasibility of using azacyclononynes for iSPAAC, we designed a synthetic access to Fmoc‐protected azacyclonon‐5‐yne (dubbed Fmoc‐ACN, **1**, Figure 2). Synthesis starts with conversion of 1,5‐cyclooctadiene (**2**) to the monoepoxide **3**, which is subjected to oxidative cleavage to afford the dialdehyde **4** (Figure 2). Reduction to the diol **5** and its activation to the ditosylate **6** is followed by reaction with *p*‐toluene sulfonamide, providing the tosylated azacyclononene **7**.^[12]^ After removal of the tosyl group,^[12]^ the liberated amine functionality is protected by an Fmoc group (**8**). Oxidation of the alkene **8** with *t*BuOOH using [Ru(cymene)Cl_2_]_2_ as a catalyst^[13]^ affords the diketone **9**, which is transformed to the bishydrazone **10**. The triple bond is generated by treatment with Pb(OAc)_4_,^[14]^ providing Fmoc‐ACN (**1**) in a total yield of 5.9 % based on cycloocta‐1,5‐diene (**2**). This compares favorably with the total yields achieved in the synthesis of the pyrrolocyclooctynes PYRROC (3.3 %),^[8]^ SYPCO (1.5 %)^[9]^ and TRIPCO (1.0 %),^[10]^ all of which are also based on 1,5‐cyclooctadiene (**2**).

The second‐order rate constant for the reaction of Fmoc‐protected ACN (**1**) with benzyl azide in CD_3_CN determined by ^1^H NMR was found to be 4.7±0.1×10^−6^ M^−1^ s^−1^ (Figure S1). The methylene protons adjacent to the triazole ring appear as two distinct signals in the ^1^H NMR at room temperature, but merge to a single resonance at 70 °C (Figure S1). This is consistent with a small rotational barrier around the nitrogen–carbon bond of the carbamate group, which is lower than the rotational barrier of amides.^[15]^ The carbamate moiety is contained in several clinically approved drugs.^[16]^

Thus, **1** reacts an order of magnitude slower with benzyl azide than the cyclononyne DIFN (*k*=5.9×10^−5^ M^−1^ s^−1^),^[11d]^ the reactivity of which in SPAAC is likely to be increased by two geminal fluorine substituents. To exclude the possibility that the fluorenyl moiety of **1** negatively affected the reactivity with azides, we also synthesized the *N*‐Boc‐protected ACN **1 a** (Figure S2). ^1^H NMR‐based analysis of the reaction kinetics of **1 a** with benzyl azide in acetonitrile at room temperature indicated approximately the same rate constant (*k*=5.9±1.6×10^−6^ M^−1^ s^−1^, Figure S3) as with Fmoc‐ACN (**1**; *k*=4.7±0.1×10^−6^ M^−1^ s^−1^, Figure S1), arguing against a major effect of the fluorenyl group on reaction kinetics.

**Figure 3:**
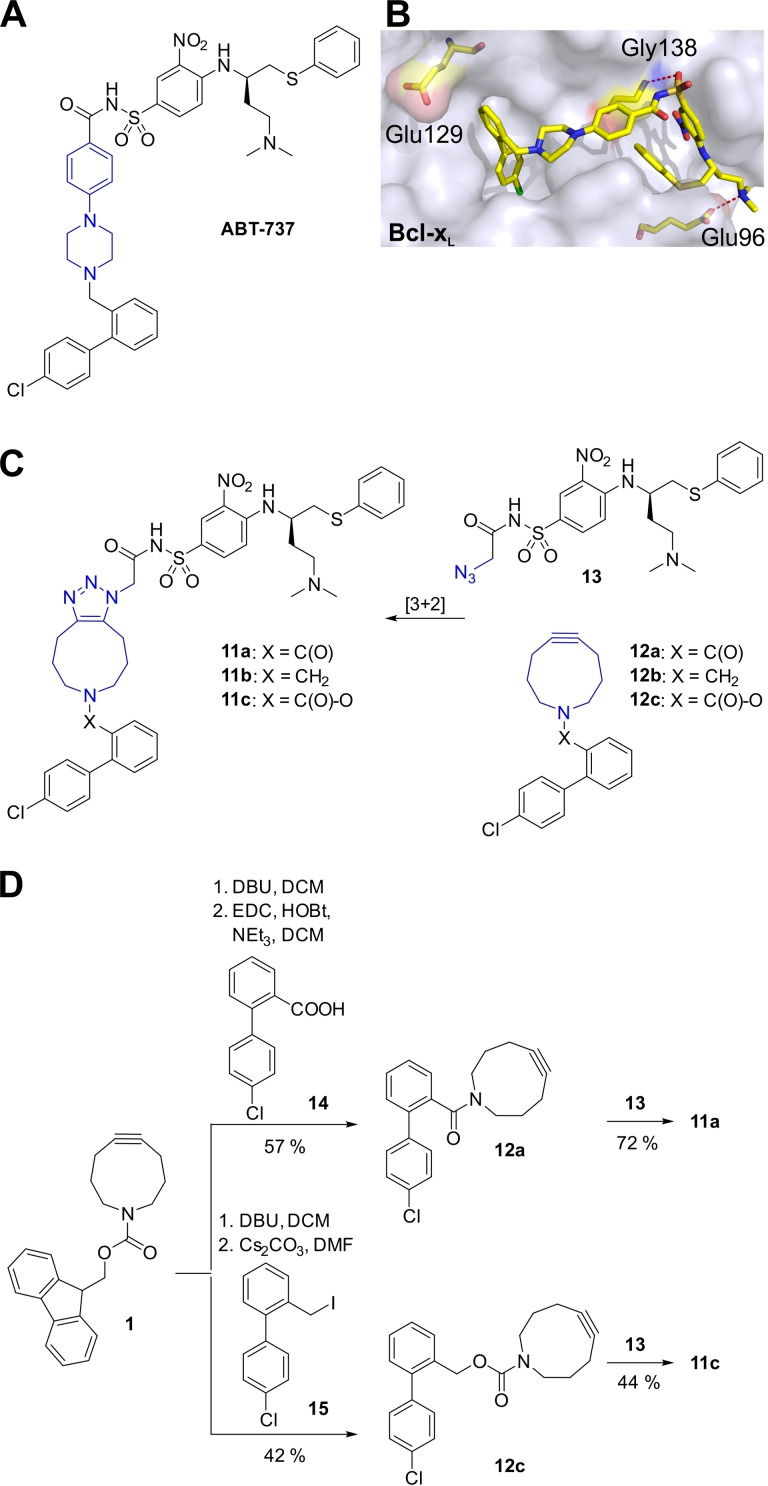
A) Structure of ABT‐737.^[17]^ B) X‐ray structure of the ABT‐737/Bcl‐x_L_ complex (PDB ID: 2YXJ).^[19]^ C) Design of the triazole‐based ABT‐737 mimetics **11**. D) Synthesis of building blocks **12 a**/**c** and triazoles **11 a**/**c**.

We aimed to explore the feasibility of using **1** for generating triazole inhibitors based on the structure of ABT‐737, a high‐affinity inhibitor of protein–protein interactions mediated by Bcl‐x_L_ (Figure 3A).^[17]^ With a molecular weight of 813 g mol^−1^, ABT‐737 is still cell‐permeable in tissue culture, but has poor oral bioavailability.^[18]^ X‐ray structure analysis of the ABT‐737/Bcl‐x_L_ complex indicated that binding of ABT‐737 is based mostly on hydrophobic interactions, supported by a salt bridge between its tertiary amine and the side chain of Glu96, and a long hydrogen bond between the acylsulfonamide and the backbone amide of Gly138 (Figure 3B).^[19]^ In the ABT‐737–based triazoles **11**, the central *N*‐phenyl piperazine core of ABT‐737, which connects the biphenyl and the acylsulfonamide moieties, was replaced by the bicyclic ring system created by iSPAAC with ACN (Figure 3C).^[9]^ The biphenyl‐derived moiety of ABT‐737 was envisaged to be linked to the deprotected azacyclononyne via an amide bond (**12 a**), a methylene bridge (**12 b**), or a carbamate linker (**12 c**, Figure 3C). The azide building block **13** contains the acylsulfonamide part of ABT‐737 (Figure 3C).^[9]^

DBU‐induced removal of the Fmoc‐protecting group of **1** and subsequent amide bond formation with the carboxylic acid **14** gave the expected building block **12 a** (Figure 3D). However, analogous treatment of **1** with DBU, followed by treatment with the benzyl iodide **15**, did not generate the expected methylene‐linked compound **12 b**. Instead, the carbamate‐linked building block **12 c** was obtained (Figure 3D). This was particularly surprising because in analogous control experiments using the Fmoc‐protected azacyclononene **8**, the expected methylene‐bridged compound was indeed isolated (Figure S4). Reaction of the functionalized cyclononynes **12 a**/**c** with the functionalized azide **13** provided the target triazoles **11 a**/**c** (Figure 3D).

**Figure 4:**
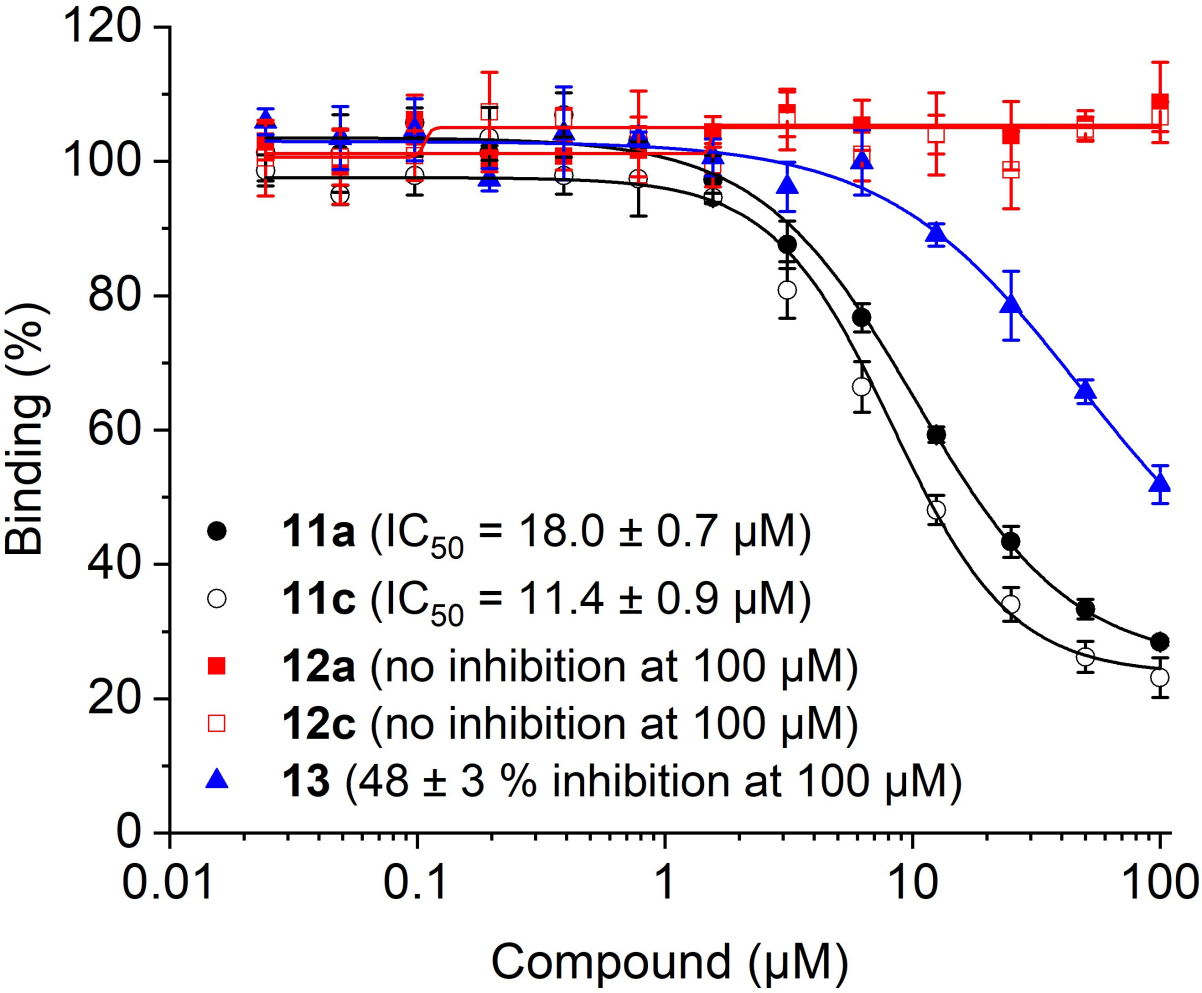
Activity of ABT‐737‐based triazoles **11 a**/**c** and their building blocks **12 a**/**c** and **13** against Bcl‐x_L_ in FP assays.

The abilities of triazoles **11 a**/**c** to interfere with binding between Bcl‐x_L_ and a fluorescein‐labeled peptide derived from the pro‐apoptotic Bcl‐2 protein Bak was analyzed in a competitive binding assay based on fluorescence polarization (FP).[^9^, ^20^] The amide‐bridged compound **11 a** (IC_50_=18.0±0.7 μM, Figure 4) was twice as active as the previously reported ABT‐737‐mimicking triazole based on the pyrrolocyclooctyne SYPCO (IC_50_=37.7±3.5 μM).^[9]^ The carbamate‐linked compound **11 c** was even more potent (IC_50_=11.4±0.9 μM), supporting the notion that the lower steric demand of the bicyclic structure formed by iSPAAC with azacyclononynes is preferable to the tricyclic structure formed with pyrrolocyclooctynes. The building blocks for the triazoles **11 a**/**c** were significantly less potent. While the azide **13** weakly inhibited Bcl‐x_L_ (48±3 % at 100 μM), the functionalized azacyclononynes **12 a**/**c** were inactive (Figure 4).

**Figure 5:**
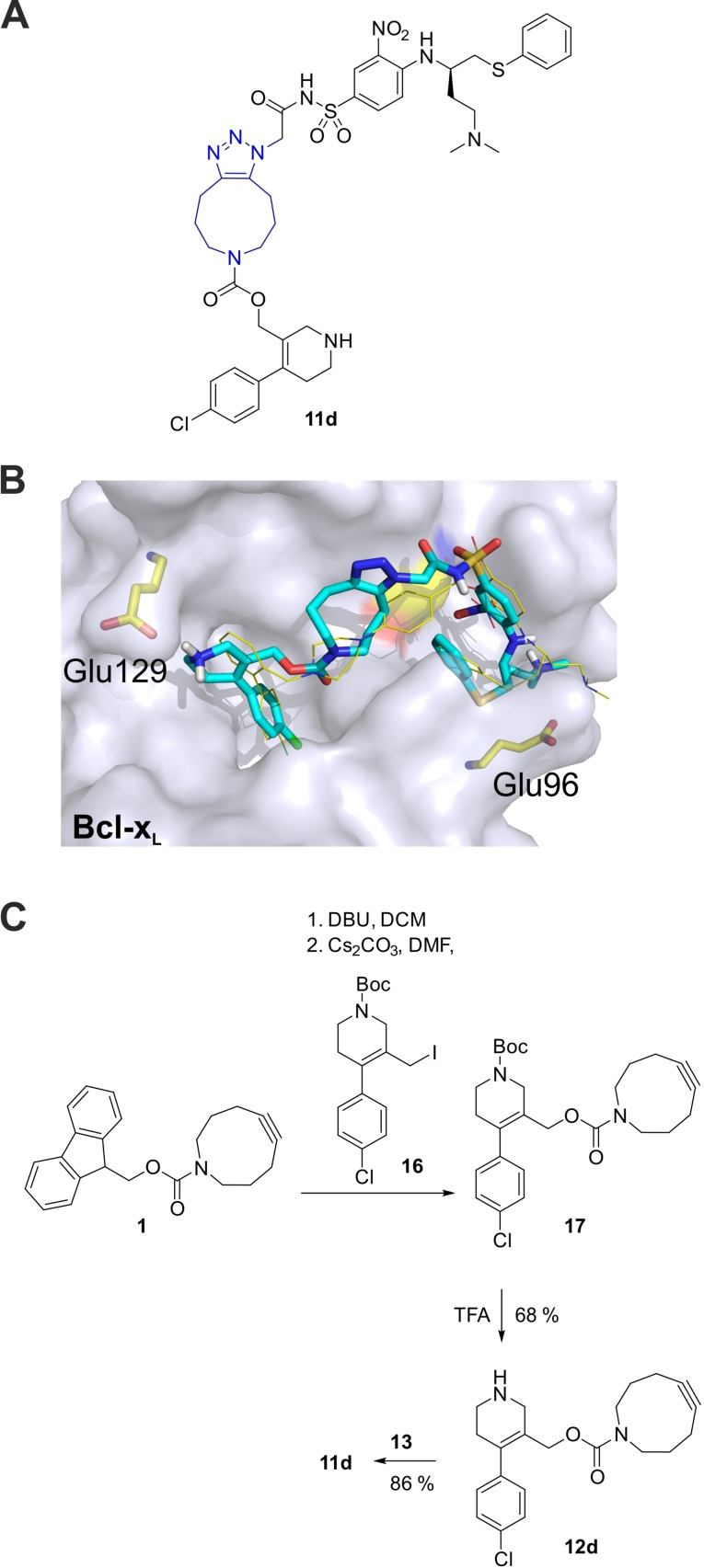
A) Structure of **11 d**. B) Docking pose of **11 d** into the co‐crystal structure of Bcl‐x_L_ and ABT‐737 (PDB ID: 2YXJ),^[19]^ from which ABT‐737 had been removed. The position of ABT‐737 is shown for comparison, with carbon atoms in yellow. The side chains of Glu129 and Glu96 were defined as flexible. C) Synthesis of **11 d**.

The X‐ray structure of ABT‐737 in complex with Bcl‐x_L_ shows that the upper end of the biphenyl moiety is in close proximity to the side chain of Glu129, without engaging in an obvious interaction (Figure 3B).^[19]^ Assuming that the biphenyl moiety of **11 c** occupies the same binding pocket as in ABT‐737 (Figure 3B), we aimed to exploit possible contributions of the side chain of Glu129 in order to improve the affinity of a triazole inhibitor. To this end, we designed the triazole **11 d**, in which the “upper” phenyl ring of the biphenyl moiety has been replaced by a tetrahydropyridine ring (Figure 5A). As the tetrahydropyridine moiety of **11 d** will be protonated at physiological pH, **11 d** was expected to engage in electrostatic interactions with Glu129, which should be reflected by a higher activity against Bcl‐x_L_ as compared to **11 c**. Molecular docking of **11 d** into Bcl‐x_L_ using AutoDockFR,^[21]^ allowing side chain flexibility for Glu129 and Glu96, supported the design concept (Figure 5B). Synthesis of the required building block **16** was achieved in a five‐step synthesis (Figure S5). DBU treatment of **1** and reaction with **16** generated the carbamate **17**. Removal of the *N*‐Boc group under carefully controlled acidic conditions generated the cycloalkyne building block **12 d**, which reacted with the azide **13** to provide the target compound **11 d**.

**Figure 6:**
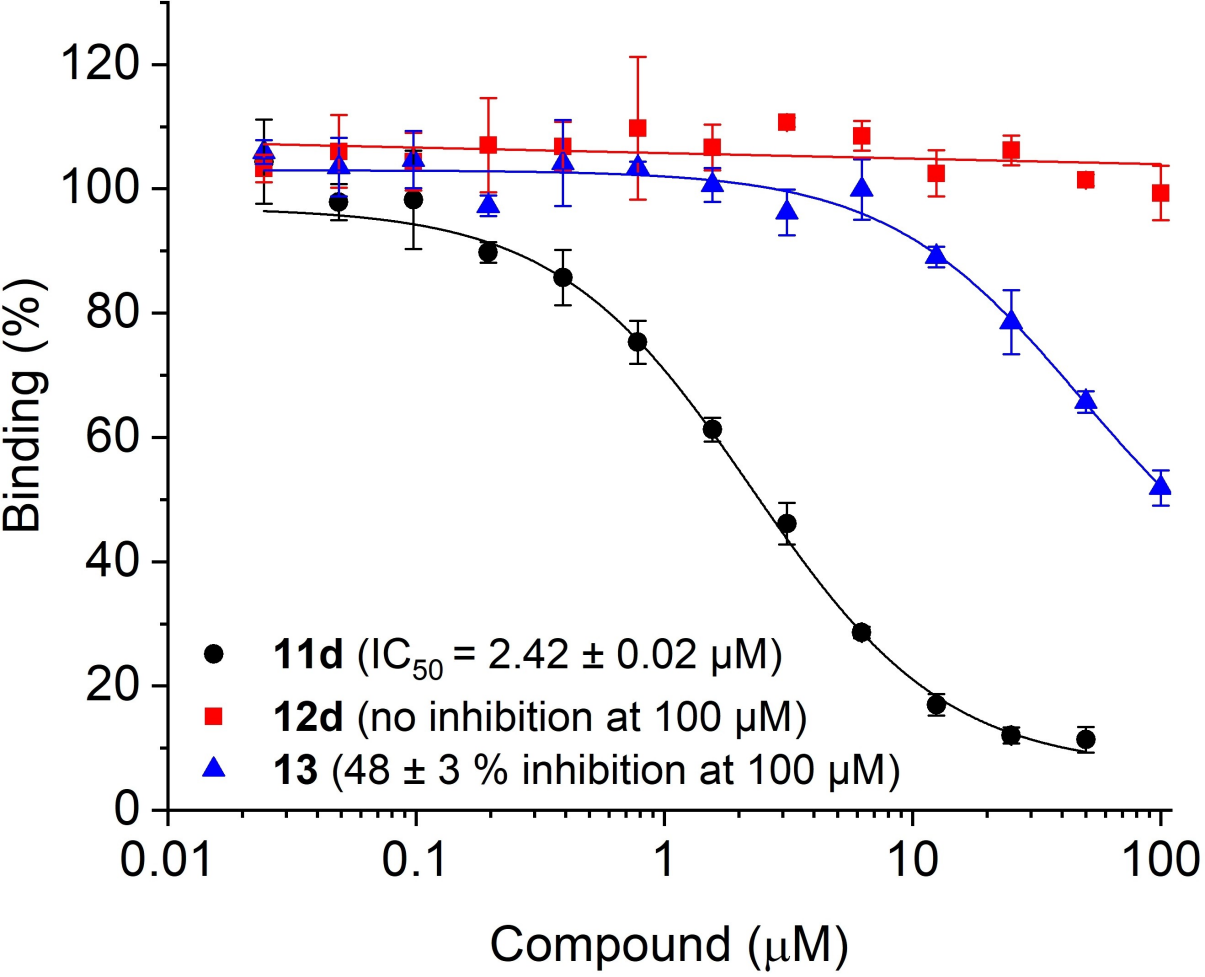
Activity of ABT‐737‐based triazole **11 d** and of the building blocks **12 d** and **13** against Bcl‐x_L_ in FP assays.

In the competitive binding assay against Bcl‐x_L_, the tetrahydropyridine **11 d** showed almost 5‐fold higher inhibitory potency (IC_50_=2.42±0.02 μM, Figure 6) than the biphenyl compound **11 c** (IC_50_=11.4±0.9 μM), demonstrating the success of the design concept. The newly developed functionalized cyclononyne **12 d** was inactive at 100 μM.

**Figure 7:**
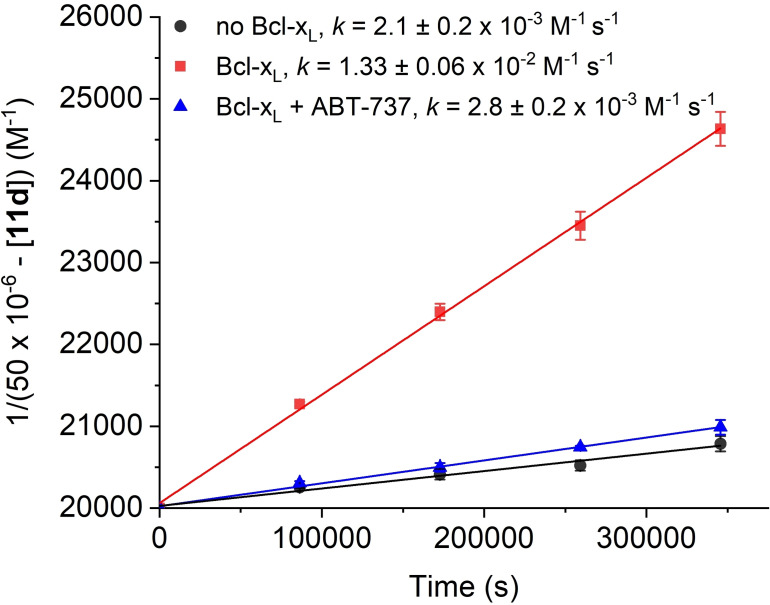
Kinetic analysis of the reaction between **12 d** and **13** to generate **11 d**.

While the reactivities of cycloalkynes are typically characterized by their second‐order rate constants in the reaction with benzylazide in CD_3_CN, or sometimes CD_3_OD, the relevant reactivity of a cycloalkyne for use in iSPAAC is the reactivity between the functionalized cycloalkyne and the functionalized azide in aqueous media at the physiological temperature of 37 °C. We therefore analyzed the kinetics of the reaction between the functionalized cycloalkyne **12 d** and the azide **13** in aqueous buffer by reversed‐phase (RP)‐HPLC. Incubation of **12 d** and **13** (both at 50 μM) at 37 °C generated the triazole **11 d** with a second‐order rate constant of 2.1±0.2×10^−3^ M^−1^ s^−1^ (Figures 7 and S6). This represents a 450‐fold rate acceleration over the reaction of **1** and benzyl azide in acetonitrile at room temperature (*k*=4.7±0.1×10^−6^ M^−1^ s^−1^). In the presence of an additional, equimolar amount of Bcl‐x_L_, this rate was further increased by a factor of 6 (*k*=1.33±0.06×10^−2^ M^−1^ s^−1^), representing a 2800‐fold higher rate constant than observed in the standard reaction between **1** and benzyl azide in acetonitrile at room temperature. In the presence of both Bcl‐x_L_ and the Bcl‐x_L_ inhibitor ABT‐737, which blocks the binding site of cycloalkyne **12 d** and the azide **13**, the template effect was largely removed (*k*=2.8±0.2×10^‐3^ M^−1^ s^−1^), thus indicating that the major component of the rate acceleration observed in the presence of the target protein was of a specific nature. These data add iSPAAC with azacyclononynes to the existing set of protein‐templated reactions.^[22]^ Of particular relevance in this context are the templated synthesis of sulfonamide‐based Bcl‐x_L_ inhibitors using sulfonyl azides and thioacids,^[23]^ and protein‐templated formation of 1,2,3‐triazoles from azides and terminal alkynes in the absence of Cu^I^.^[24]^

Incubation of **12 d** and **13** at 50 μM at 37 °C to afford the triazole **11 d** proceeded with a 450‐fold higher rate constant (*k*=2.1±0.2×10^−3^ M^−1^ s^−1^, Figures 7 and S6) than the reaction between Fmoc‐ACN and benzyl azide in acetonitrile at room temperature (26 °C, *k*=4.7±0.1×10^−6^ M^−1^ s^−1^, Figure S1). The divergent rate constants could be caused by the differences in reaction temperatures, in the polarity of the reaction media, and in the electronic nature of the reactants, or by a combination of these factors. In order to analyze the influence of the reaction temperature on the second‐order rate constant, we carried out the reaction between **1** and benzyl azide in acetonitrile at 37 °C, and observed a 3.2‐fold rate acceleration (*k*=1.5±0.02×10^−5^ M^−1^ s^−1^; Figure S7) compared to the standard conditions at room temperature (26 °C, *k*=4.7±0.1×10^−6^ M^−1^ s^−1^; Figure S1).

SPAAC rate constants tend to increase in more polar environments.[^5b^, ^5c^, ^7^, ^8^, ^9^, ^25^] Possible explanations for this effect include the stabilization of the transition state by the reduction of the total water‐exposed hydrophobic surface area, which is entropically favorable,^[26]^ and/or by hydrogen‐bonding effects.^[27]^ Increases in rate constants of up to 28‐fold have been determined by supplementing organic solvents with an increasing proportion of water, and rate accelerations exceeding 100‐fold have been extrapolated when changing from an entirely organic solvent to an entirely aqueous environment.^[25]^ In order to assess the effect of the solvent, we carried out the reaction between **12 d** and **13** at 37 °C in [D_6_]DMSO. The reaction proceeded with a rate constant of *k*=3.7±0.01×10^−5^ M^−1^ s^−1^ (Figure S8), which is ∼57 times slower than between **12 d** and **13** at 37 °C in aqueous buffer (*k*=2.1±0.2×10^−3^ M^−1^ s^−1^; Figure 7). The use of [D_6_]DMSO instead of CD_3_CN, which was required for solubility reasons, does not appear to significantly affect the reaction rate, given that the reaction between **1** and benzyl azide proceeds only slightly faster in [D_6_]DMSO (*k*=5.7±0.7×10^−6^ M^−1^ s^−1^; Figure S9) than in CD_3_CN (*k*=4.7±0.1×10^−6^ M^−1^ s^−1^; Figure S1).

Thus, the combined effects of increased temperature (3.2‐fold) and the exchange of an organic solvent for aqueous buffer (∼57‐fold) account for a ∼180‐fold rate increase for the reaction between **12 d** and **13** at 37 °C in aqueous buffer, as compared to the reaction between **1** and benzyl azide in [D_6_]DMSO at room temperature. This leaves a reactivity difference of ∼2.5‐fold for the reaction between **12 d** and **13** as compared to the reaction between Fmoc‐ACN and benzyl azide to be explained by electronic effects. While possible electronic reasons include a smaller HOMO–LUMO energy gap between the pairs of reactants (**12 d** reacting with **13**, versus Fmoc‐ACN reacting with benzyl azide),^[28]^ the scope of accelerating SPAAC with azacyclononynes through electronic effects will be analyzed in future studies.

## Conclusion

In summary, we report the design, synthesis and application of Fmoc‐ACN (**1**) as the first cyclononyne suitable for isomer‐free SPAAC. Fmoc‐ACN was used for the structure‐guided development of the triazole **11 d**, an inhibitor of the protein–protein interaction domain of the antiapoptotic protein Bcl‐x_L_ (IC_50_=2.42±0.02 μM). Compound **11 d** is the most potent inhibitor developed by iSPAAC to date. While the second‐order rate constant of **1** in the reaction with benzyl azide in CD_3_CN was low (*k*=4.7±0.1×10^−6^ M^−1^ s^−1^), the reaction of a functionalized azacyclononyne based on **1** with a functionalized azide in aqueous buffer at 37 °C generated a triazole mimetic of the Bcl‐x_L_ inhibitor ABT‐737 with a 450‐fold higher rate. This rate of reaction was further increased by a sixfold template effect in the presence of the target protein Bcl‐x_L_, resulting in a 2800‐fold rate acceleration compared to the reaction between **1** and benzyl azide under standard conditions. Our data demonstrate the utility of azacyclononynes for iSPAAC and their potential for achieving vastly accelerated, templated reactions in the aqueous environment.

## Experimental Section

NMR spectra were recorded at ambient temperature unless stated otherwise using Varian Mercury plus 400 (400 MHz for ^1^H NMR, 100 MHz for ^13^C NMR, 376 MHz for ^19^F NMR), Varian Mercury plus 300 (300 MHz for ^1^H NMR, 75 MHz for ^13^C NMR, 282 MHz for ^19^F NMR), Bruker Avance III HD 400 (400 MHz) and Bruker Fourier 300 (300 MHz) spectrometers. Chemical shifts are given in parts per million (ppm). ^1^H NMR spectra and ^13^C NMR spectra are referenced to the signal of the residual solvent (^1^H NMR: CDCl_3_ 7.26 ppm, CD_2_Cl_2_ 5.32 ppm, CD_3_CN 1.94 ppm, [D_6_]DMSO 2.50 ppm; ^13^C NMR: CDCl_3_ 77.16 ppm, CD_2_Cl_2_ 53.84 ppm, CD_3_CN 1.32 ppm, [D_6_]DMSO 39.52 ppm).^[29]^ ^19^F NMR spectra are referenced to CCl_3_F as external standard. High‐resolution electrospray ionization (HR‐ESI) mass spectra were recorded on a Bruker Daltonics Impact II and Bruker Daltonics micrOTOF time of flight mass spectrometer (ESI‐TOF‐MS). High‐resolution electron ionization (HR‐EI) mass spectra were obtained on a Finnigan MAT 95XP GC‐MS system. UV/Vis spectra were recorded with a Jasco UV‐Vis‐V‐630 spectrometer. The substances were dissolved and measured in the specified organic solvent. The absorption maxima are given in nanometers. IR spectra were measured with a Jasco FT/IR‐4100 fourier transform infrared spectrometer using a KBr pellet or film. Wave numbers and signal intensities (s=strong, m=medium, w=weak, b=broad) are shown. Uncorrected melting points were measured using a Rapido PHMK apparatus from Veb Wägetechnik or melting point M‐560 apparatus from Büchi.

Analytical RP‐HPLC measurements were conducted on a Beckman Coulter System Gold^®^ 125NM HPLC System equipped with a Knauer Variable Wavelength Monitor (detection at 214 nm) and a Phenomenex Jupiter C_18_ column (internal diameter: 2 mm, length: 150 mm, particle size: 5 μm, pore size: 30 nm). The flow rate was set to 0.2 mL min^−1^. Samples were eluted using a linear gradient from 20 to 80 % eluent B in 60 min. Eluent A: 0.1 % (*v*/*v*) TFA in water. Eluent B: 0.1 % TFA in 90 % (*v*/*v*) aqueous acetonitrile.

**Kinetic analysis by** ^**1**^ **H NMR**: Azacyclononyne [Fmoc‐ACN (**1**), Boc‐ACN (**1 a**), or **12 d**] and azide (benzyl azide or **13**) were separately dissolved in CD_3_CN or [D_6_]DMSO at 30 mM and 150 mM, respectively. The stock solutions were rapidly mixed in a 5 : 1 ratio to give a final concentration of 25 mM for each of the reactants. ^1^H NMR spectra were recorded immediately at the indicated temperature. Unless stated otherwise, the conversion was calculated from the integral of the methylene protons of the azide (−CH_2_−N_3_) relative to the integral of the corresponding methylene protons in the triazole product. The second‐order rate constant *k* was determined by plotting the reciprocal of the concentration of benzyl azide or **12 d** versus time. Linear regression gave the second‐order rate constant *k* as the slope of the linear curve.

**Kinetic analysis by HPLC**: **12 d** (50 μM) and **13** (50 μM) were incubated at 37 °C in the absence of Bcl‐x_L_, in the presence of Bcl‐x_L_ (50 μM), and in the presence of both Bcl‐x_L_ (50 μM) and ABT‐737 (50 μM). At the given time points, aliquots of 60 μL were diluted with 60 μL of 0.1 % TFA in 4.5 % acetonitrile/H_2_O and analyzed by RP‐HPLC (100 μL injection volume). The formation of **11 d** was quantified by peak area using a standard curve. 1/(50×10^−6^−[**11 d**]) [M^−1^] was plotted against time. Linear regression gave the second‐order rate constant *k* as the slope. Experiments were carried out in triplicate. Buffer composition: 10 mM Tris pH 8.0, 50 mM NaCl, 1 mM EDTA, 0.1 % Nonidet P‐40 substitute, and 4 % DMSO.

**Fluorescence polarization assays**: Assays were performed essentially as described previously.[^20^, ^30^] The protein Bcl‐x_L_ (amino acids 1‐209, Δ45–84, kindly provided by Prof. Ho Sup Yoon, Nanyang Technological University, Singapore)^[31]^ was incubated with the test compounds for 1 h at room temperature at a protein concentration of 37 nM. Subsequently, fluorophore‐labeled Bak BH3‐derived peptide (5‐carboxyfluorescein‐GQVGRQLAIIGDDINR‐NH_2_ was added (final peptide concentration: 4 nM), and the fluorescence polarization was measured after another 60 min in a plate reader (Tecan Infinite F500). All experiments were carried out in triplicate. Buffer composition: 10 mM Tris pH 8.0, 50 mM NaCl, 1 mM EDTA, 0.1 % Nonidet P‐40 substitute, 1 mM DTT, and 2 % DMSO.

**Synthesis of compound 8**: A Schlenk tube was equipped with 2.06 g (16.1 mmol, 6.2 equiv.) naphthalene and THF (42 mL). 370 mg (16.1 mmol, 6.2 equiv.) sodium were added and the solution was stirred for 20 min at room temperature. The freshly prepared sodium naphthalenide solution was added dropwise to a solution of 726 mg (2.06 mmol, 1.0 equiv.) **7** in THF (10 mL) at −78 °C.^[12]^ The reaction mixture was stirred for 20 min at this temperature and subsequently quenched by the addition of a saturated NaHCO_3_ solution (15 mL). The mixture was extracted with ethyl acetate (6×50 mL). The combined organic layers were dried over Na_2_SO_4_ and the solvent was removed in vacuo. The crude product was used directly in the next step without further purification. To the deprotected amine in THF (10 mL) at 0 °C was added a solution of 744 mg (7.02 mmol, 2.7 equiv.) Na_2_CO_3_ in water (5 mL). 1.01 g (3.90 mmol, 1.5 equiv.) 9‐fluorenylmethoxycarbonyl chloride were added and the reaction was stirred for 18 h. The mixture was extracted with ethyl acetate (3×30 mL). The combined organic layers were dried over Na_2_SO_4_ and purified by column chromatography (*n*‐hexane/ethyl acetate, 20 : 1 to 10 : 1, *v/v*) to give **8** as a colorless oil, which solidified upon standing. Yield: 555 mg (1.60 mmol, 62 %).

**Synthesis of compound 9**: Under ambient atmosphere 22.8 mg (65.6 μmol, 1.0 equiv.) **8**, 7.27 mg (19.7 μmol, 0.3 equiv.) TBAI and 0.40 mg (0.66 μmol, 0.01 equiv.) [Ru(*p*‐cymene)Cl_2_]_2_ were dissolved in toluene (0.42 mL), acetonitrile (0.42 mL) and water (0.08 mL). 32.3 μL (70 wt. % in water, 30.4 mg, 337 μmol, 3.6 equiv.) *tert*‐butyl hydroperoxide solution were added and the reaction mixture was stirred for 1 h at room temperature. The reaction was quenched by the addition of a saturated Na_2_SO_3_ solution (1 mL) and it was extracted with ethyl acetate (3×30 mL). The combined organic layers were dried over Na_2_SO_4_ and purified by column chromatography (*n*‐hexane/ethyl acetate, 10 : 1 to 5 : 1, *v/v*) to give **9** as a colorless oil. Yield: 14.8 mg (39.2 μmol, 60 %).

**Synthesis of compound 10**: To a solution of 25.9 μL (26.6 mg, 531 μmol, 3.3 equiv.) hydrazine monohydrate in ethanol (0.16 mL) at −20 °C was added a solution of 60.0 mg (159 μmol, 1.0 equiv.) **9** in ethanol (0.13 mL). The reaction mixture was stirred for 1 h at −20 °C and for 30 min at room temperature. The solvent was removed in vacuo to give **10** as a colorless oil, which was used directly in the next step without further purification. Yield: 61.1 mg (151 μmol, 95 %).

**Synthesis of Fmoc‐ACN** (**1**): 44.5 mg (110 μmol, 1.0 equiv.) **10** were dissolved in DCM (0.25 mL) and cooled to −20 °C. A solution of 105 mg (236 μmol, 2.1 equiv.) lead tetraacetate (***CAUTION***! Lead tetraacetate is highly toxic!) in DCM (0.25 mL) was added slowly over a period of 15 min and the mixture was stirred for 1.5 h at −20 °C. The reaction was quenched by the addition of a saturated NaHCO_3_ solution (5 mL) and it was extracted with DCM (3×30 mL). The combined organic layers were dried over Na_2_SO_4_, adsorbed onto silica and purified by column chromatography (*n*‐hexane/acetone, 20 : 1 to 10 : 1, *v/v*) to give **1** as a colorless oil. Yield: 29.6 mg (85.7 μmol, 78 %).

## Conflict of interest

The authors declare no conflict of interest.

1

## Supporting information

As a service to our authors and readers, this journal provides supporting information supplied by the authors. Such materials are peer reviewed and may be re‐organized for online delivery, but are not copy‐edited or typeset. Technical support issues arising from supporting information (other than missing files) should be addressed to the authors.

Supporting Information

## Data Availability

The data that support the findings of this study are available in the supplementary material of this article.
