# Supplementary material for: Templated Generation of a Bcl‐xL Inhibitor by Isomer‐Free SPAAC Based on Azacyclonon‐5‐yne
Source: Chemistry. 2022 Oct 1;28(66):e202202259. doi: 10.1002/chem.202202259 (PMC9827882; doi:10.1002/chem.202202259)
Supplement: Supplementary file 1 — Supporting Information [file CHEM-28-0-s001.pdf]

Chemistry–A European Journal

Supporting Information

Templated Generation of a Bcl-x_L Inhibitor by Isomer-Free SPAAC Based on Azacyclonon-5-yne

Juliane Brauer, Marina Mötzing, Corinna Gröst, Ralf Hoffmann, and Thorsten Berg*

Table of contents

Figure S1 2
Figure S2 3
Figure S3 4
Figure S4 5
Figure S5 5
Figure S6 6
Figure S7 10
Figure S8 11
Figure S9 12
Synthesis and spectroscopic characterization of compounds 13
NMR spectra 32
Supporting references 49

Figure S1

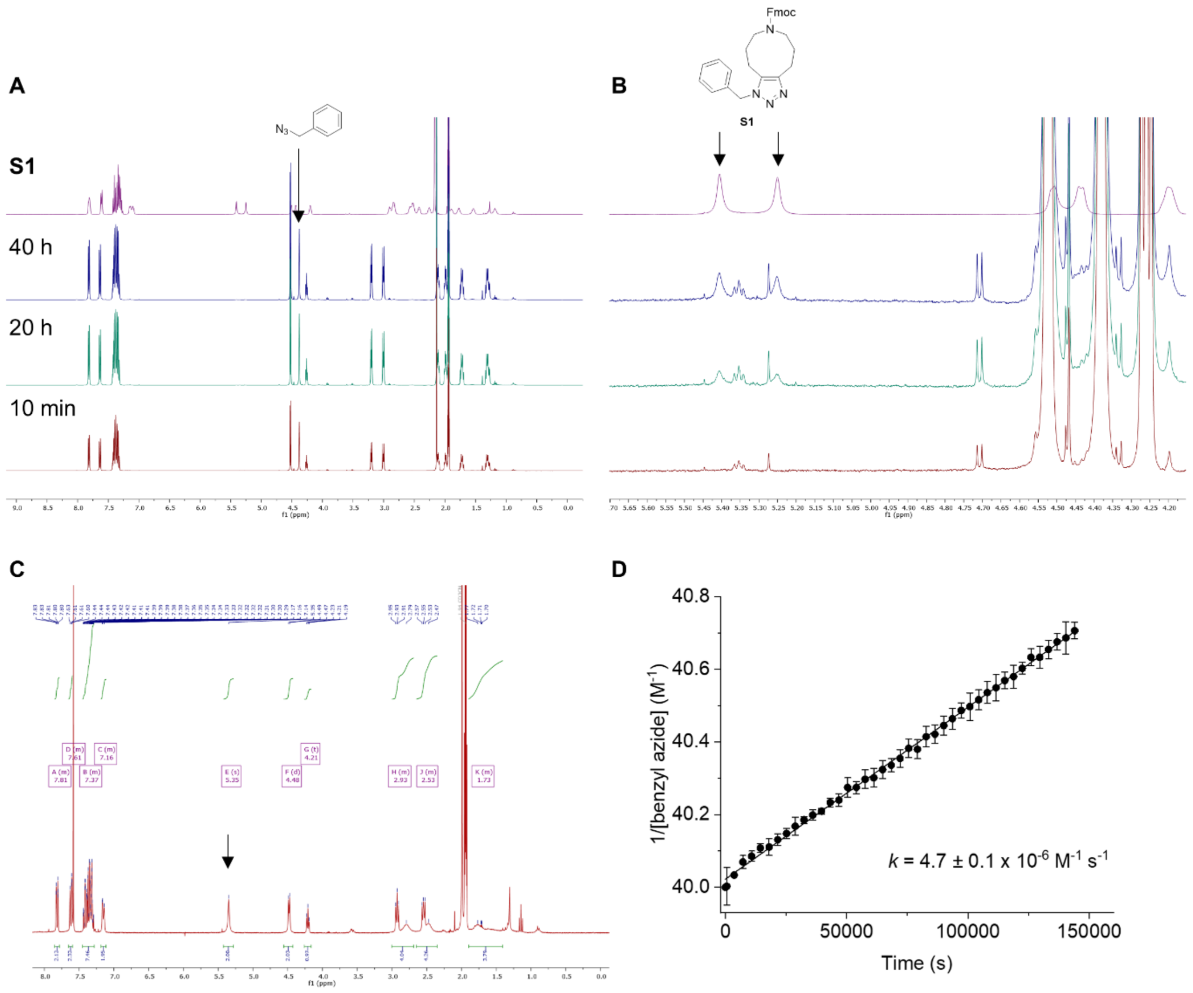

Figure S1. Determination of second-order rate constant of Fmoc-ACN (**1**) in the reaction with benzyl azide at 26 °C in CD_3CN by ^{1}H-NMR spectroscopy. A) ^{1}H-NMR spectra of the cycloaddition were recorded at the indicated time points after addition of benzyl azide. The spectrum of the purified triazole **S1** is shown on top. B) Close-up of the spectra shown in A) with a focus on the methylene protons of **S1**. Progress of the reaction is indicated by the increase of the signals of the methylene protons of **S1** at 5.41 ppm and 5.25 ppm. C) ^{1}H-NMR spectrum of **S1** at 70 °C shows only one singlet of the methylene protons at 5.35 ppm, indicating that the appearance of 2 signals for the methylene protons at room temperature is due to the rotational barrier of the carbamate bond. D) The second-order rate constant was determined by plotting 1/[benzyl azide] versus time. The plot was fitted to a linear regression and the slope corresponds to the second-order rate constant *k*. Mean values and standard deviations from three independent experiments (n = 3) are shown.

Figure S2

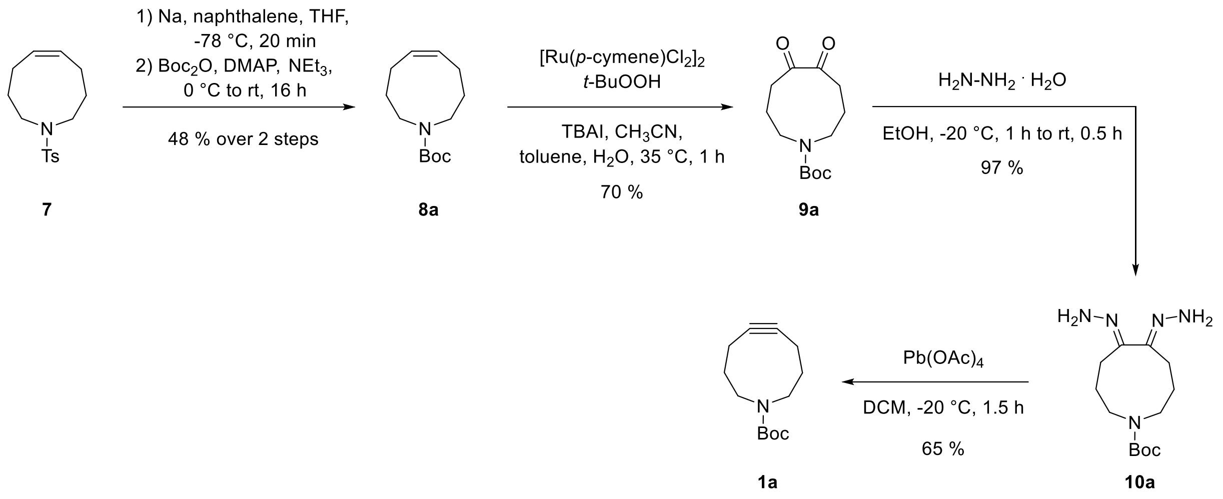

Figure S2. Synthesis of the Boc-protected azacyclononyne **1a**.

Figure S3

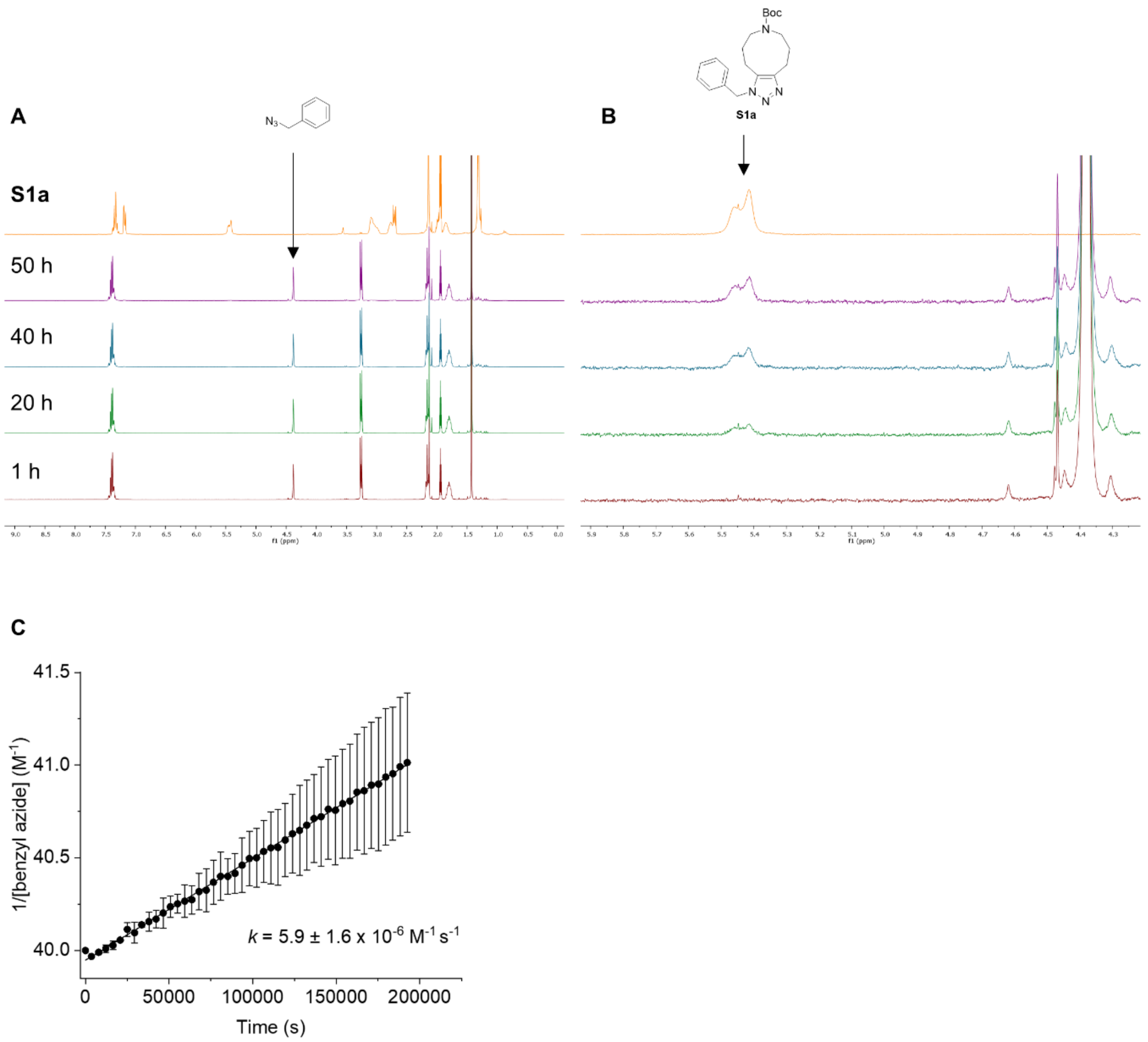

Figure S3. Determination of second-order rate constant of Boc-ACN (**1a**) in the reaction with benzyl azide at 26 °C in CD_3CN by ^{1}H-NMR spectroscopy. A) ^{1}H-NMR spectra of the cycloaddition were recorded at the indicated time points after addition of benzyl azide. The spectrum of the purified triazole **S1a** is shown on top. B) Close-up of the spectra shown in A) with a focus on the methylene protons of **S1a**. Progress of the reaction is indicated by the increase of the signals of the methylene protons of **S1a** at 5.46 ppm and 5.41 ppm. C) The second-order rate constant was determined by plotting 1/[benzyl azide] versus time. The plot was fitted to a linear regression and the slope corresponds to the second-order rate constant *k*. Mean values and standard deviations from two independent experiments (n = 2) are shown.

Figure S4

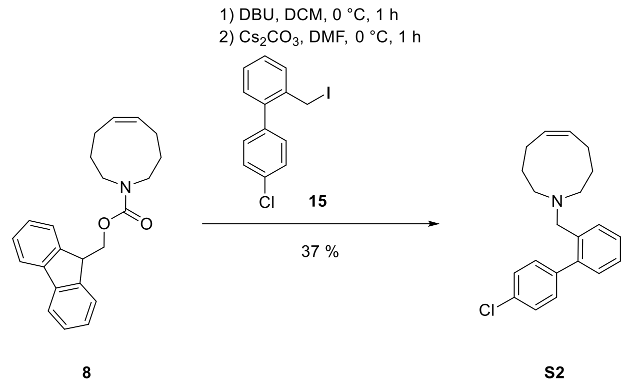

Figure S4. Deprotection of Fmoc-protected azacyclononene **8** and conversion with benzyl iodide **15** provides the methylene bridged compound **S2**.

Figure S5

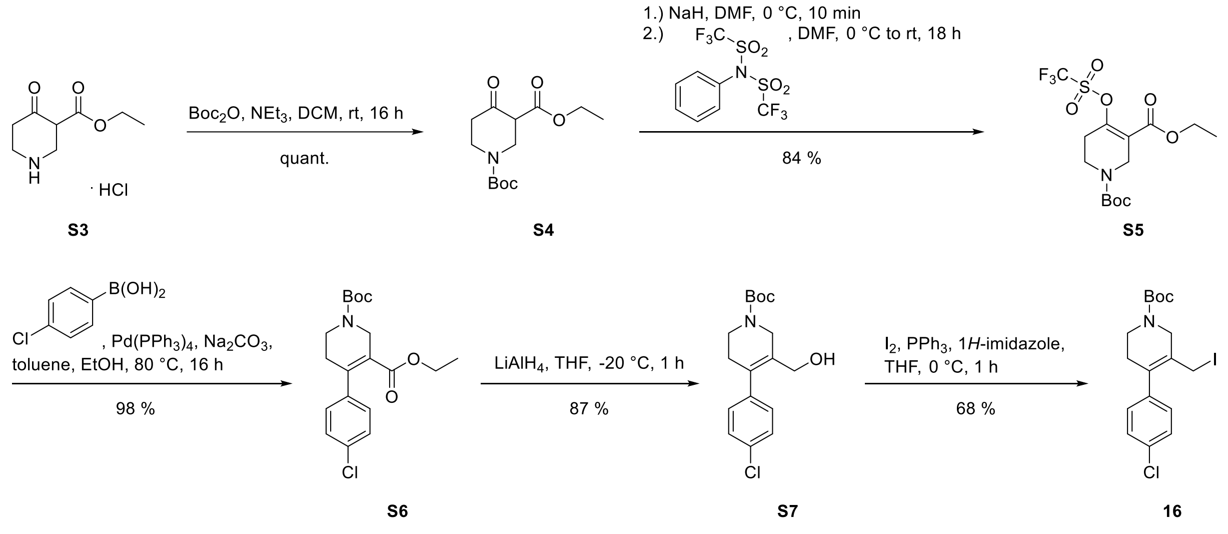

Figure S5. Synthesis of the building block **16**.

Figure S6

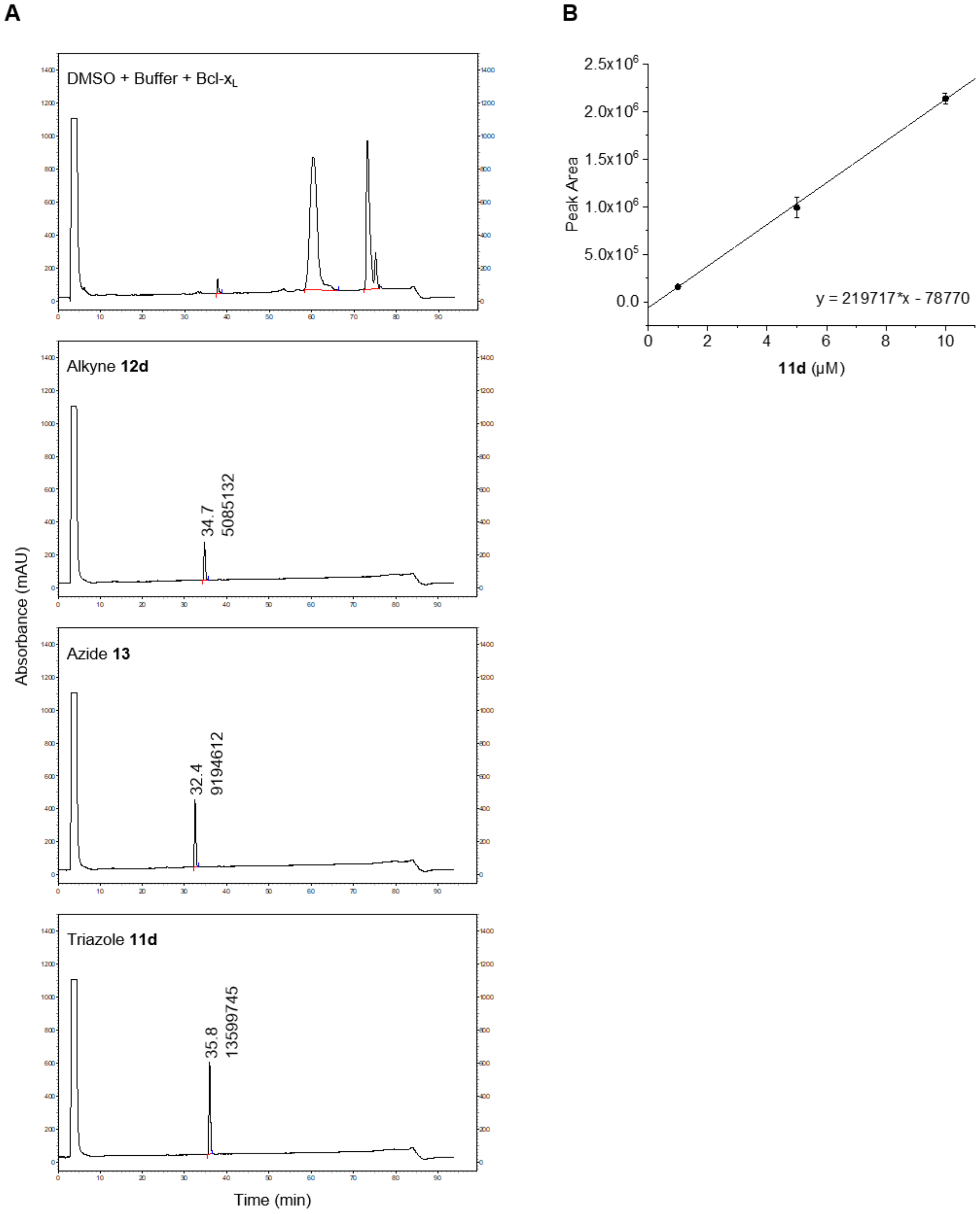
A
B
DMSO + Buffer + Bcl-xL
Alkyne 12d
34.7
5085132
Azide 13
32.4
9194612
Triazole 11d
35.8
13599745
Absorbance (mAU)
Time (min)
Peak Area
11d (μM)
y = 219717*x - 78770

Figure S6 (continued)

C - Bcl-x_L

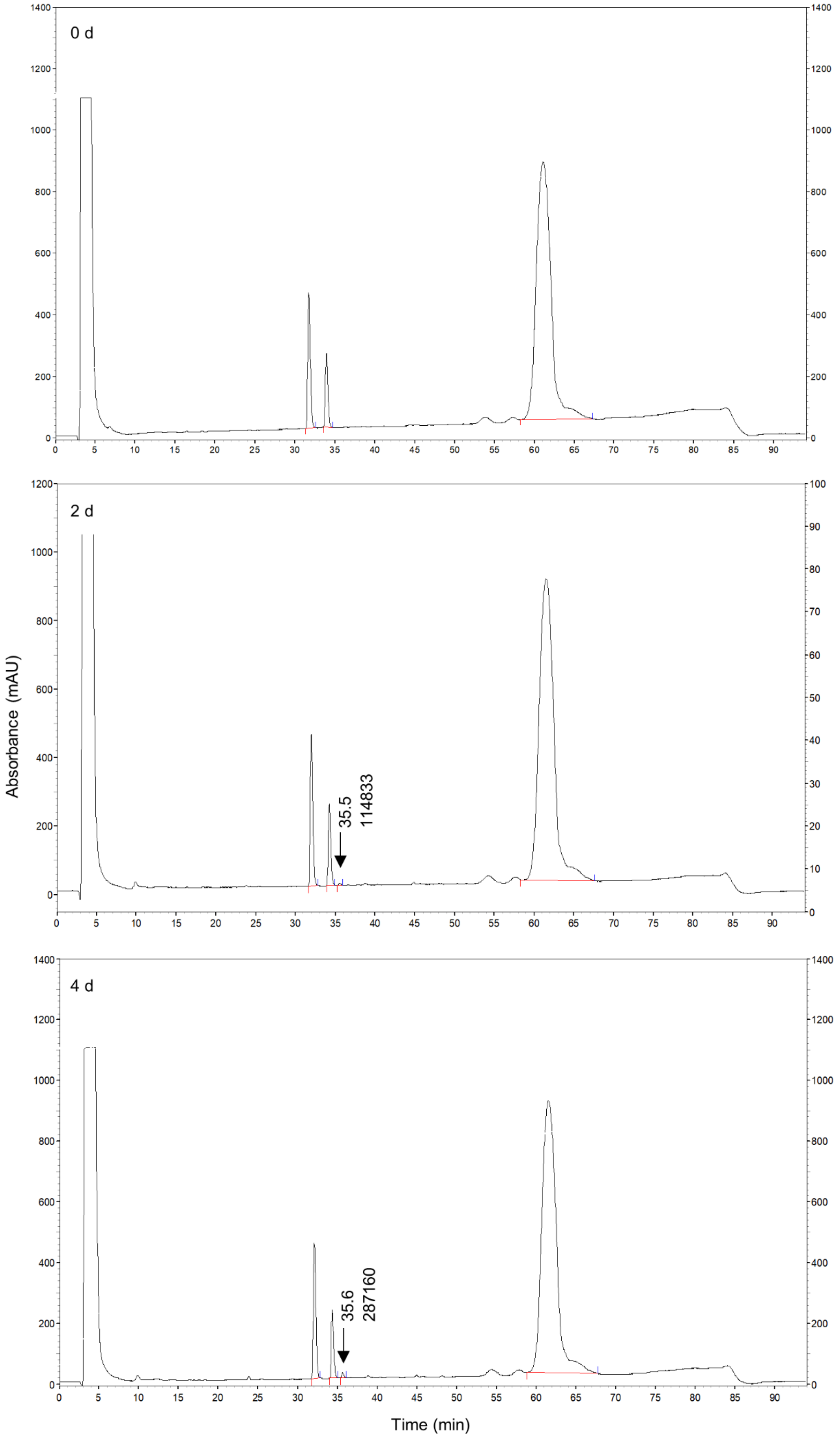

Figure S6 (continued)

D + Bcl-x_L

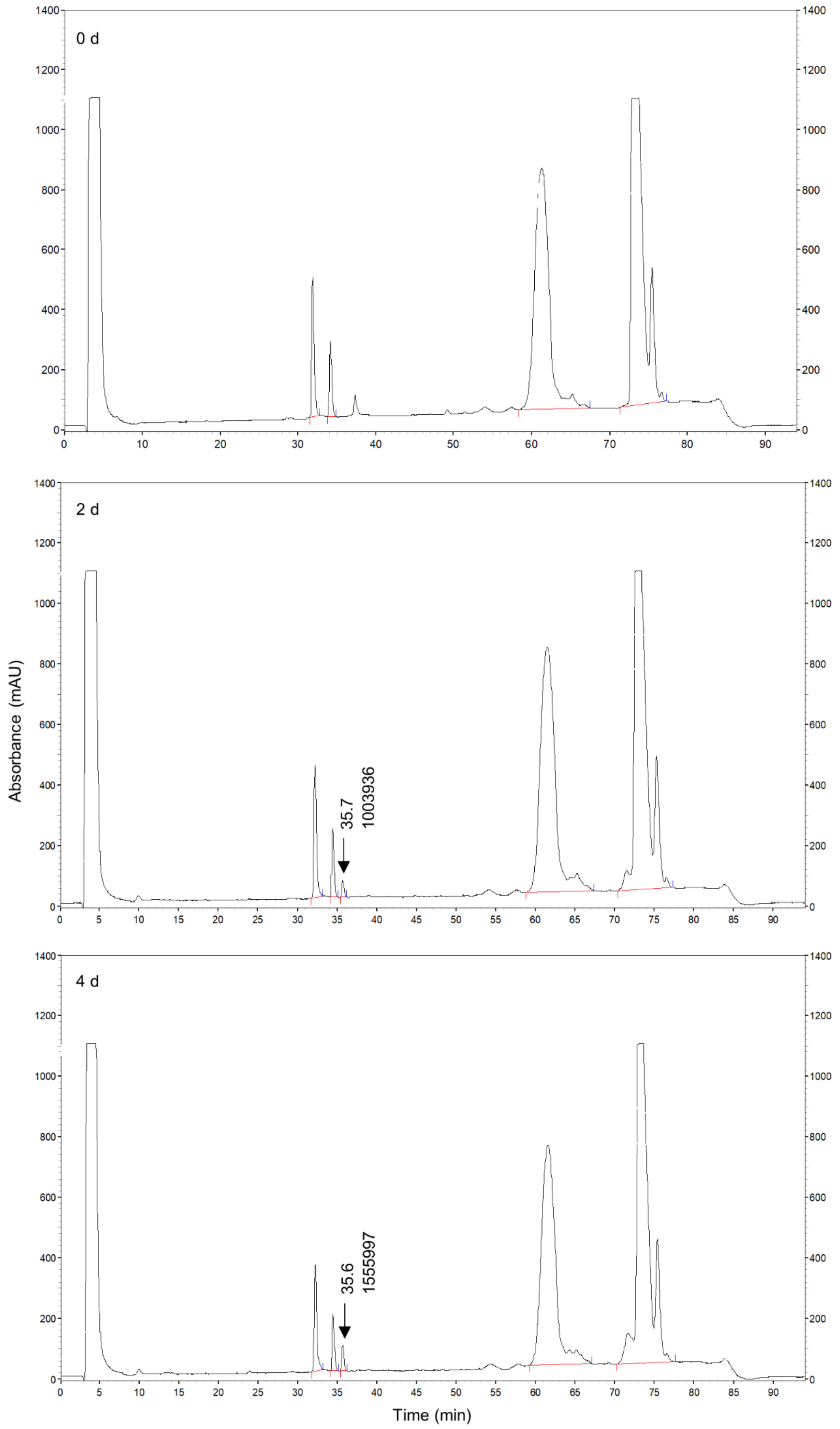

Figure S6 (continued)

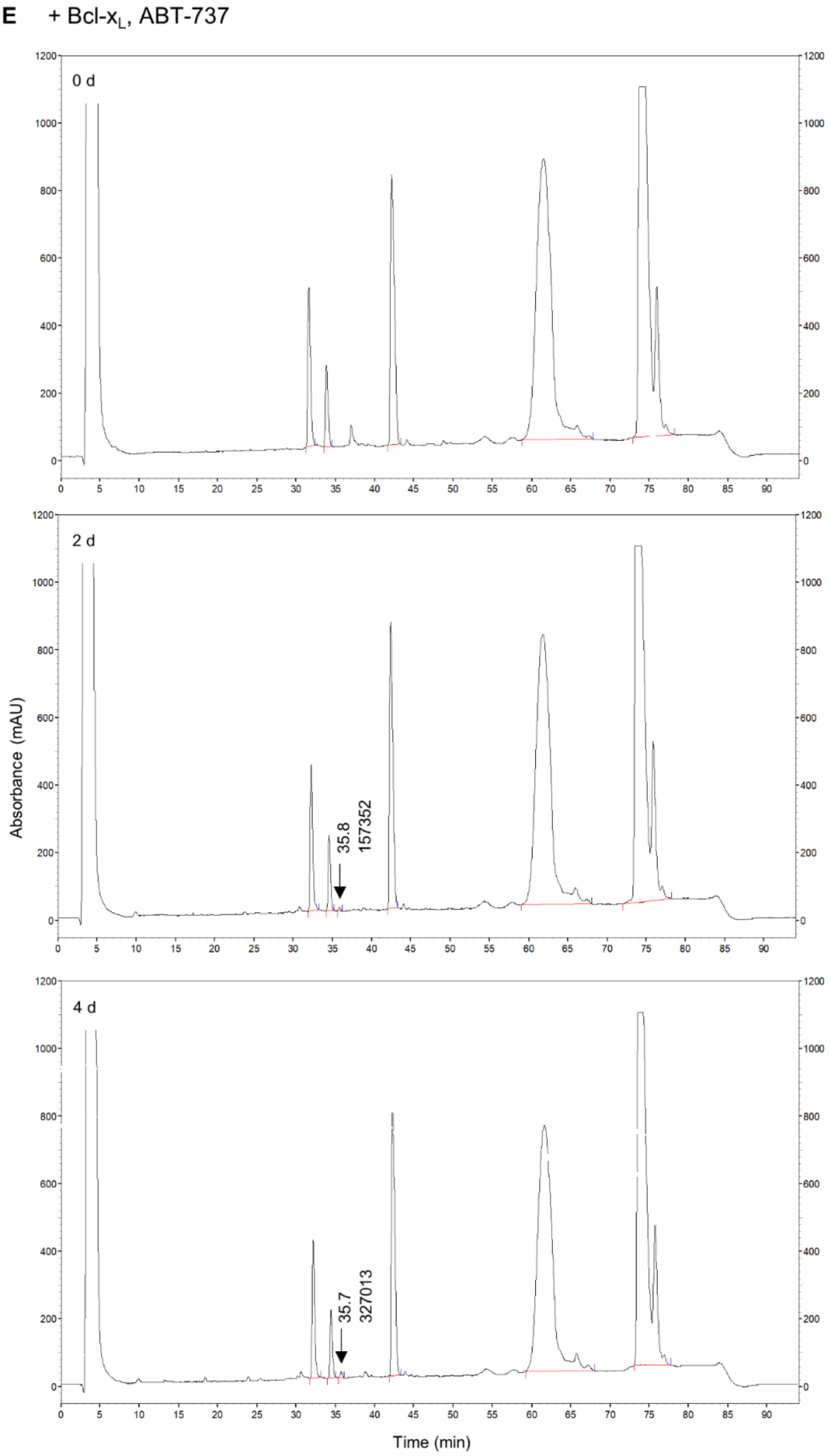

Figure S6. HPLC analysis of the *in vitro* generation of **11d**. A) HPLC traces of buffer components, alkyne **12d**, azide **13** and pre-synthesized triazole **11d** (all dissolved in DMSO). The retention times and peak integrals are given. B) Standard curve for **11d**. Mean values and standard deviations from three independent experiments (n = 3) are shown. C)-E) HPLC analysis of the reaction between **12d** and **13** at 37 °C after mixing (0 d), after 2 d and 4 d, C) in the absence of Bcl-xL, D) in the presence of Bcl-xL and E) in the presence of both Bcl-xL and ABT-737. All experiments were carried out in triplicate.

Figure S7

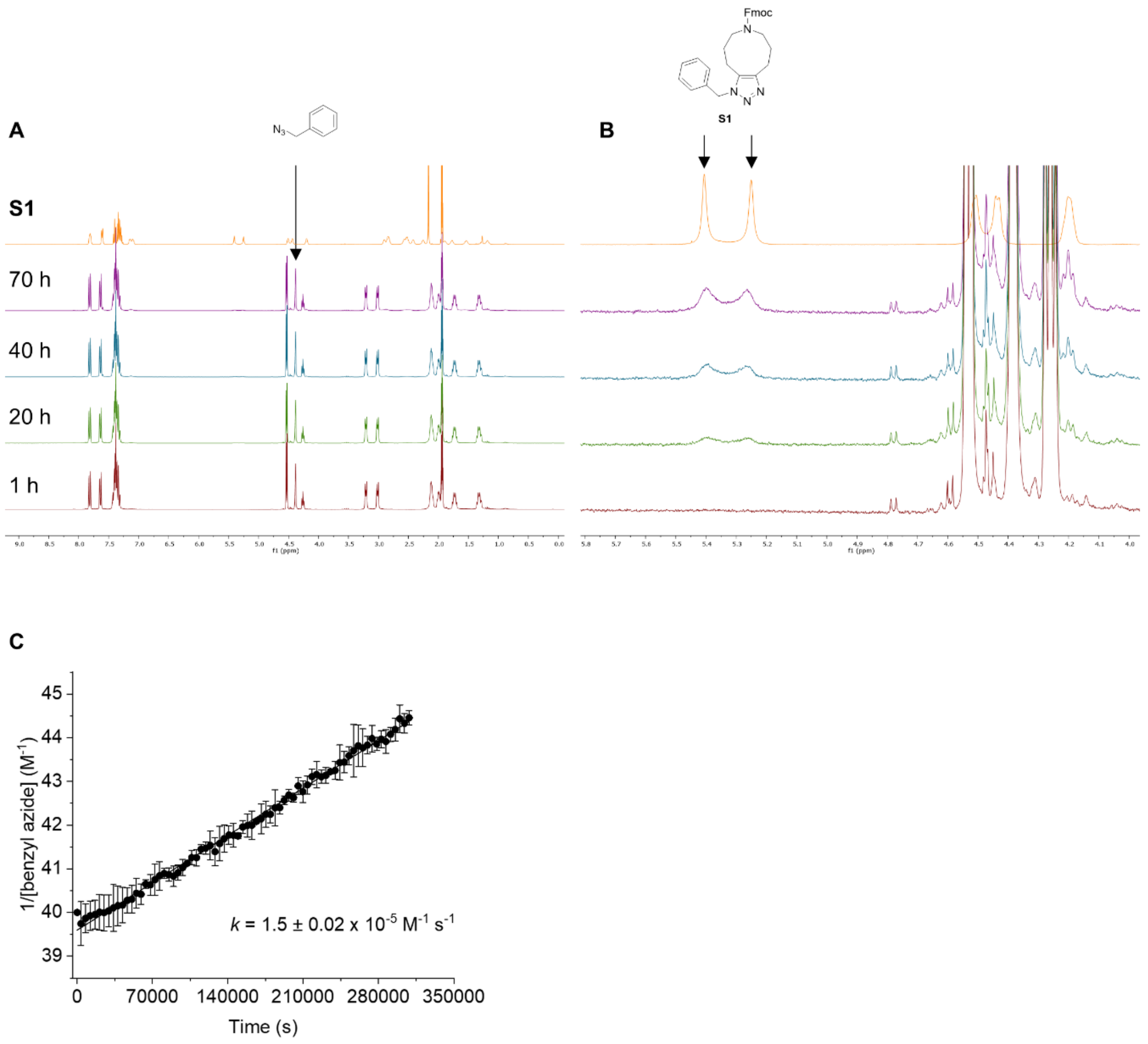

Figure S7. Determination of second-order rate constant of Fmoc-ACN (**1**) in the reaction with benzyl azide at 37 °C in CD_3CN by ^{1}H-NMR spectroscopy. A) ^{1}H-NMR spectra of the cycloaddition were recorded at the indicated time points after addition of benzyl azide. The spectrum of the purified triazole **S1** is shown on top. B) Close-up of the spectra shown in A) with a focus on the methylene protons of **S1**. Progress of the reaction is indicated by the increase of the signals of the methylene protons of **S1** at 5.40 ppm and 5.26 ppm. C) The second-order rate constant was determined by plotting 1/[benzyl azide] versus time. The plot was fitted to a linear regression and the slope corresponds to the second-order rate constant k. Mean values and standard deviations from two independent experiments (n = 2) are shown.

Figure S8

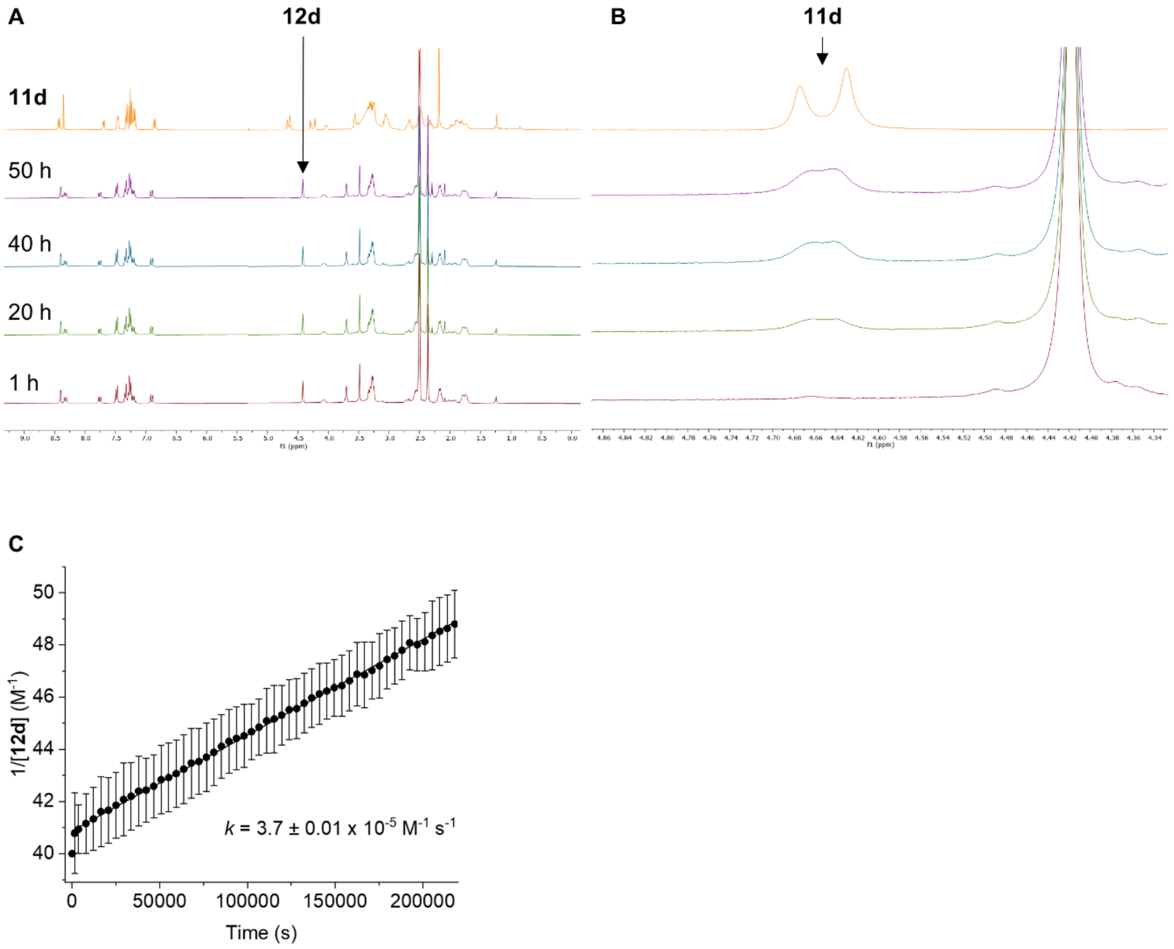

Figure S8. Determination of second-order rate constant of **12d** in the reaction with azide **13** at 37 °C in DMSO-d_6 by ^{1}H-NMR spectroscopy. A) ^{1}H-NMR spectra of the cycloaddition were recorded at the indicated time points after addition of azide **13**. The spectrum of the purified triazole **11d** is shown on top. B) Close-up of the spectra shown in A) with a focus on the methylene protons of **11d**. Progress of the reaction is indicated by the increase of the signals of the methylene protons of **11d** at 4.67 ppm and 4.63 ppm. The integrals were corrected by subtracting the background. C) The second-order rate constant was determined by plotting 1/[**12d**] versus time. The plot was fitted to a linear regression and the slope corresponds to the second-order rate constant *k*. Mean values and standard deviations from two independent experiments (n = 2) are shown.

Figure S9

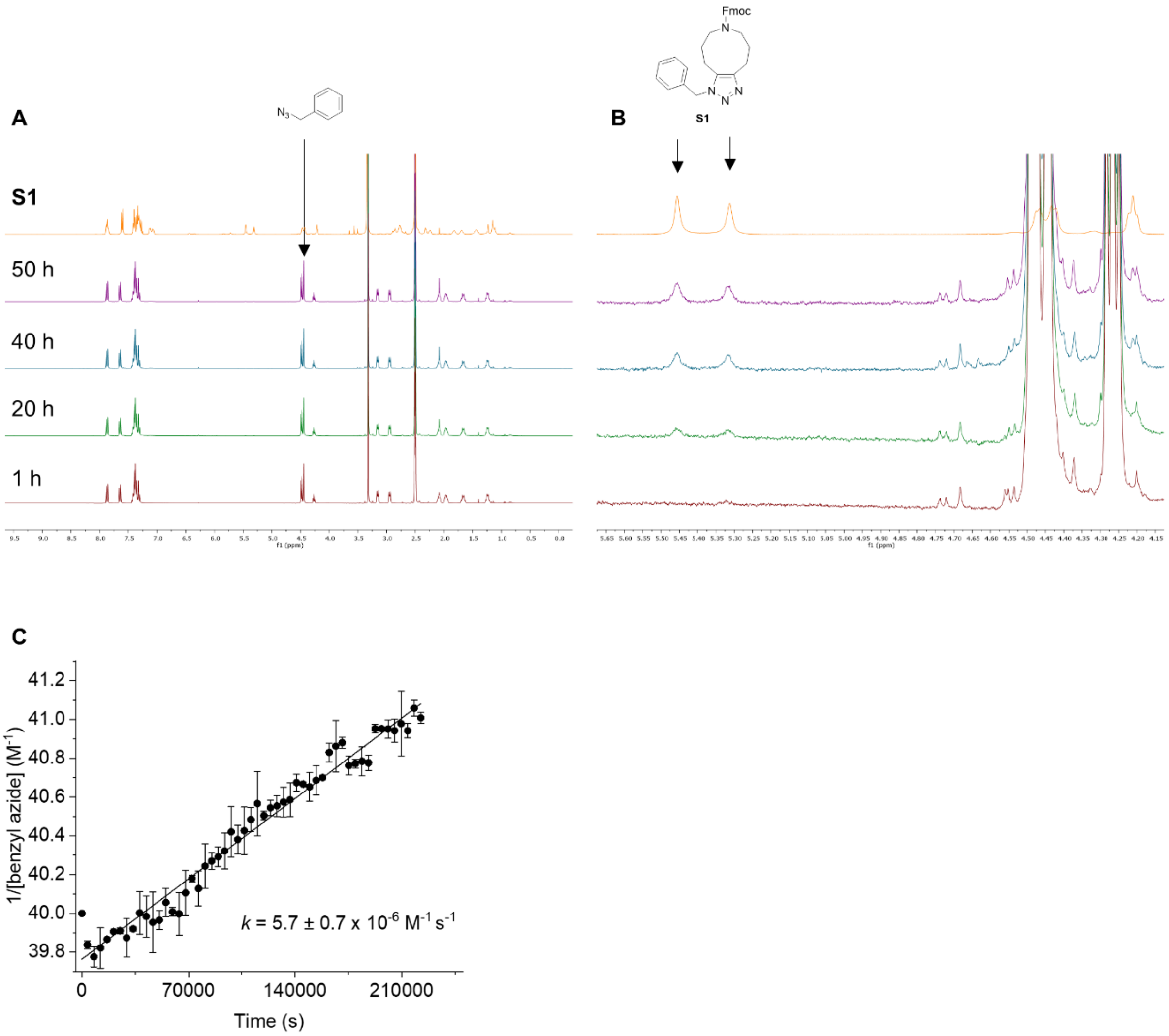

Figure S9. Determination of second-order rate constant of Fmoc-ACN (**1**) in the reaction with benzyl azide at 26 °C in DMSO-d_6 by ^{1}H-NMR spectroscopy. A) ^{1}H-NMR spectra of the cycloaddition were recorded at the indicated time points after addition of benzyl azide. The spectrum of the purified triazole **S1** is shown on top. B) Close-up of the spectra shown in A) with a focus on the methylene protons of **S1**. Progress of the reaction is indicated by the increase of the signals of the methylene protons of **S1** at 5.45 ppm and 5.32 ppm. C) The second-order rate constant was determined by plotting 1/[benzyl azide] versus time. The plot was fitted to a linear regression and the slope corresponds to the second-order rate constant *k*. Mean values and standard deviations from two independent experiments (n = 2) are shown.

Synthesis and spectroscopic characterization of compounds

All air and moisture sensitive reactions were carried out in oven-dried glassware, using dry solvents and under an inert gas atmosphere (argon or nitrogen). Chemicals were purchased from SIGMA ALDRICH, ABCR, ACROS ORGANICS, ALFA AESAR, MERCK, FLUKA, FLUOROCHEM, EURISO-TOP and TCI EUROPE. Dry solvents were either obtained from commercial sources or dried using standard methods (THF over sodium and benzophenone, ethanol over sodium and diethyl phthalate, and DCM over CaH_2). Solvents were degassed by repeated ultrasonic treatment under vacuum. All water used was deionized.

(1*R*,8*S*,*Z*)-9-Oxabicyclo[6.1.0]non-4-ene (**3**) [1]

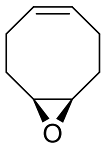

11.0 mL (9.74 g, 90.0 mmol, 1.0 equiv.) (*Z*,*Z*)-1,5-cyclooctadiene were dissolved in DCM (144 mL) and cooled to 0 °C. 18.6 g (≤ 77 wt. %, 108 mmol, 1.2 equiv.) *m*-chloroperoxybenzoic acid were added slowly and the mixture was stirred for 2 h. The reaction was quenched by the addition of a saturated $Na_2S_2O_3$ solution (20 mL). The precipitate formed was filtered off and it was extracted with DCM (3 x 30 mL). The combined organic layers were dried over Na_2SO_4 and purified by column chromatography (DCM) to give the desired product as a colorless liquid. **Yield**: 7.50 g (60.4 mmol, 67 %). $\mathbf{R_f}$ = 0.58 (*n*-hexane/ethyl acetate, 5:1, *v*/*v*). **^{1}H NMR** (400 MHz, $CDCl_3$): δ = 5.61 – 5.50 (m, 2H), 3.08 – 2.97 (m, 2H), 2.51 – 2.35 (m, 2H), 2.20 – 1.95 (m, 6H) ppm. **^{13}C NMR** (101 MHz, $CDCl_3$): δ = 128.9, 56.7, 28.1, 23.7 ppm. **IR** (film): $\tilde{\nu}$ = 3006 (m), 2958 (m), 2884 (m), 2836 (w), 1656 (w), 1486 (m), 1445 (w), 1428 (w), 1289 (w), 1228 (w), 1099 (w), 1040 (w), 935 (m), 863 (m), 763 (w), 746 (w), 697 (w), 621 (w), 459 (w), 412 (w) cm^{-1}. **HR-ESI-MS** (MeOH) m/z: $C_8H_{12}O$ calc.: 147.0780 $[M+Na]^+$, meas.: 147.0788.

(*Z*)-Oct-4-enedial (**4**) [2]

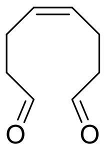

To a solution of 6.97 g (56.1 mmol, 1.0 equiv.) **3** in 1,4-dioxane (28 mL) at 0 °C were added dropwise 12.8 g (56.1 mmol, 1.0 equiv.) periodic acid in water (28 mL). The mixture was stirred for 1.5 h at 0 °C. Water (20 mL) was added and it was extracted with ethyl acetate (3 x 50 mL). The combined organic layers were dried over Na_2SO_4 and purified by column chromatography (*n*-hexane/ethyl acetate, 10:1 to 5:1, *v*/*v*) to give the desired product as a

colorless liquid. **Yield**: 4.66 g (33.3 mmol, 59 %). **R_f** = 0.20 (*n*-hexane/ethyl acetate, 5:1, *v*/*v*). **^{1}H NMR** (300 MHz, $CDCl_3$): δ = 9.77 (t, J = 1.5 Hz, 1H), 5.45 – 5.31 (m, 2H), 2.56 – 2.47 (m, 4H), 2.43 – 2.33 (m, 4H) ppm. **^{13}C NMR** (101 MHz, $CDCl_3$): δ = 201.9, 128.9, 43.6, 20.0 ppm. **IR** (film): ṽ = 3425 (w), 3012 (w), 2958 (w), 2895 (w), 2828 (w), 2728 (m), 1723 (s), 1446 (w), 1411 (w), 1390 (m), 1351 (w), 1112 (w), 1057 (w), 904 (w), 859 (w), 733 (w), 689 (w), 649 (w), 596 (w), 451 (w) cm^{-1}. **UV/Vis** (DCM): λ_{max} = 290, 228 nm. **HR-ESI-MS** (MeOH) m/z: $C_8H_{12}O_2$ calc.: 163.0729 $[M+Na]^+$, meas.: 163.0737.

(*Z*)-Oct-4-ene-1,8-diol (**5**) [2]

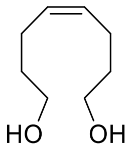

4.66 g (33.3 mmol, 1.0 equiv.) **4** were dissolved in methanol (40 mL) and cooled to 0 °C. 2.52 g (66.5 mmol, 2.0 equiv.) sodium borohydride were added in three portions. The reaction mixture was stirred for 1 h at 0 °C. The reaction was quenched by the addition of a saturated NH_4Cl solution (20 mL). The mixture was extracted with diethyl ether (3 x 50 mL). The combined organic layers were dried over Na_2SO_4 and purified by column chromatography (*n*-hexane/ethyl acetate, 1:1, *v*/*v*) to give the desired product as a colorless liquid. **Yield**: 3.60 g (25.0 mmol, 75 %). **R_f** = 0.37 (ethyl acetate). **^{1}H NMR** (300 MHz, $CDCl_3$): δ = 5.46 – 5.35 (m, 2H), 3.65 (t, J = 6.2 Hz, 4H), 2.25 – 2.13 (m, 4H), 1.75 (s, 2H), 1.69 – 1.57 (m, 4H) ppm. **^{13}C NMR** (101 MHz, $CDCl_3$): δ = 130.0, 62.0, 32.4, 23.4 ppm. **IR** (film): ṽ = 3334 (s, br), 3006 (m), 2936 (s), 2864 (s), 1655 (w), 1473 (m), 1454 (m), 1405 (w), 1374 (w), 1350 (w), 1279 (w), 1179 (w), 1165 (w), 1105 (w), 1061 (s), 1039 (s), 914 (w), 764 (w), 732 (m), 595 (w) cm^{-1}. **HR-ESI-MS** (MeOH) m/z: $C_8H_{16}O_2$ calc.: 167.1042 $[M+Na]^+$, meas.: 167.1038.

(*Z*)-Oct-4-ene-1,8-diyl bis(4-methylbenzenesulfonate) (**6**) [3]

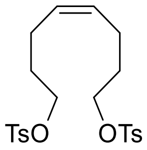

To a solution of 3.60 g (25.0 mmol, 1.0 equiv.) **5** in DCM (33 mL) at 0 °C were added 6.66 mL (6.52 g, 82.4 mmol, 3.3 equiv.) pyridine, followed by 14.3 g (74.9 mmol, 3.0 equiv.) 4-methylbenzenesulfonyl chloride. The mixture was stirred for 18 h at room temperature and subsequently quenched by the addition of water (100 mL) and 1 M HCl (10 mL). The mixture was extracted with DCM (3 x 50 mL). The combined organic layers were washed with saturated $NaHCO_3$ solution (50 mL) and brine (50 mL), dried over Na_2SO_4 and purified by column chromatography (*n*-hexane/ethyl acetate, 5:1, *v*/*v*) to give the desired product as a colorless

oil.[5] **Yield**: 10.7 g (23.5 mmol, 94 %). **R_f** = 0.15 (*n*-hexane/ethyl acetate, 5:1, *v*/*v*). **^{1}H NMR** (400 MHz, $CDCl_3$): δ = 7.79 (d, J = 8.3 Hz, 4H), 7.35 (d, J = 8.1 Hz, 4H), 5.33 – 5.22 (m, 2H), 4.01 (t, J = 6.4 Hz, 4H), 2.45 (s, 6H), 2.08 – 1.95 (m, 4H), 1.73 - 1.60 (m, 4H) ppm. **^{13}C NMR** (101 MHz, $CDCl_3$): δ = 144.9, 133.3, 130.0, 129.3, 128.0, 70.0, 28.8, 23.1, 21.8 ppm. **IR** (film): ṽ = 3008 (w), 2958 (w), 2923 (w), 2849 (w), 1598 (m), 1455 (w), 1358 (s), 1292 (w), 1189 (s), 1176 (s), 1097 (m), 1018 (w), 966 (m), 927 (m), 834 (m), 816 (m), 741 (m), 664 (s), 575 (m), 555 (s) cm^{-1}. **UV/Vis** (DCM): λ_{max} = 263, 233 nm. **HR-ESI-MS** (MeOH) m/z: $C_{22}H_{28}O_6S_2$ calc.: 475.1219 $[M+Na]^+$, meas.: 475.1241.

(*Z*)-1-Tosyl-2,3,4,7,8,9-hexahydro-1*H*-azonine (**7**) [3]

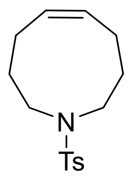

A three-necked round-bottomed flask was charged with 18.0 g (449 mmol, 208 equiv.) NaOH, 1.12 g (3.03 mmol, 1.4 equiv.) TBAI, toluene (585 mL) and water (43 mL). One attached dropping funnel was charged with a solution of 978 mg (2.16 mmol, 1.0 equiv.) **6** in toluene (121 mL) and the other with a suspension of 740 mg (4.32 mmol, 2.0 equiv.) 4-methylbenzenesulfonamide in toluene (121 mL). The mixture was heated to 100 °C and subsequently the reactants were added dropwise at the same rate (note: during the addition heating of the $TsNH_2$ mixture was necessary to maintain a solution) and refluxed for 2 h. The mixture was cooled to room temperature and water (50 mL) was added. The phases were separated and the aqueous phase was extracted with diethyl ether (3 x 100 mL). The combined organic layers were dried over Na_2SO_4 and purified by column chromatography (*n*-hexane/ethyl acetate, 50:1 to 30:1 to 10:1, *v*/*v*) to give the desired product as a colorless solid. **Yield**: 464 mg (1.66 mmol, 77 %). **R_f** = 0.48 (*n*-hexane/ethyl acetate, 5:1, *v*/*v*). **m.p.**: 97 – 99 °C. **^{1}H NMR** (400 MHz, $CDCl_3$): δ = 7.72 (d, J = 8.3 Hz, 2H), 7.31 (d, J = 7.9 Hz, 2H), 5.57 – 5.45 (m, 2H), 2.97 (t, J = 6.3 Hz, 4H), 2.50 – 2.34 (m, 7H), 1.90 – 1.80 (m, 4H) ppm. **^{13}C NMR** (101 MHz, $CDCl_3$): δ = 143.3, 134.6, 130.3, 129.6, 127.8, 53.5, 28.5, 22.4, 21.6 ppm. **IR** (KBr): ṽ = 3435 (m,br), 3005 (m), 2966 (w), 2922 (m), 2901 (m), 1468 (m), 1360 (m), 1339 (s), 1300 (m), 1178 (m), 1156 (s), 1135 (m), 1095 (m), 1066 (m), 973 (s), 879 (m), 808 (m), 713 (m), 688 (s), 639 (m), 549 (s) cm^{-1}. **UV/Vis** (DCM): λ_{max} = 267, 231 nm. **HR-ESI-MS** (MeOH) m/z: $C_{15}H_{21}NO_2S$ calc.: 302.1185 $[M+Na]^+$, meas.: 302.1196.

(9*H*-Fluoren-9-yl)methyl (*Z*)-2,3,4,7,8,9-hexahydro-1*H*-azonine-1-carboxylate (**8**)

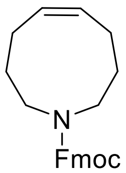

Synthesis of **8** has been described in the main part of the manuscript.

R$_f$ = 0.45 (*n*-hexane/ethyl acetate, 5:1, *v*/*v*). **m.p.**: 79 – 81 °C. **^{1}H NMR** (400 MHz, $CDCl_3$): δ = 7.75 (d, J = 7.5 Hz, 2H), 7.61 (d, J = 7.4 Hz, 2H), 7.45 – 7.28 (m, 4H), 5.50 – 5.29 (m, 2H), 4.62 (d, J = 5.3 Hz, 2H), 4.22 (t, J = 5.3 Hz, 1H), 3.11 (t, J = 6.2 Hz, 2H), 2.95 (t, J = 6.3 Hz, 2H), 2.09 (q, J = 7.2 Hz, 2H), 1.93 (q, J = 7.1 Hz, 2H), 1.82 (p, J = 6.3 Hz, 2H), 1.36 (p, J = 6.3 Hz, 2H) ppm. **^{13}C NMR** (101 MHz, $CDCl_3$): δ = 157.1, 144.4, 141.7, 129.9, 129.8, 127.7, 127.1, 124.8, 120.0, 66.0, 53.1, 51.9, 47.8, 26.4, 25.8, 22.6, 22.5 ppm. **IR** (KBr): ṽ = 3436 (m, br), 3004 (m), 2952 (m), 2916 (m), 2903 (m), 2855 (m), 1709 (s), 1484 (s), 1450 (s), 1416 (s), 1376 (m), 1360 (m), 1346 (m), 1316 (s), 1286 (s), 1223 (s), 1176 (s), 1114 (s), 1095 (m), 1052 (s), 770 (m), 735 (s), 720 (s) cm^{-1}. **UV/Vis** (DCM): λ_{max} = 301, 290, 267, 228 nm. **HR-ESI-MS** (MeOH) m/z: $C_{23}H_{25}NO_2$ calc.: 370.1777 [M+Na]$^+$, meas.: 370.1795.

(9*H*-Fluoren-9-yl)methyl 5,6-dioxoazonane-1-carboxylate (**9**)

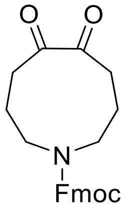

Synthesis of **9** has been described in the main part of the manuscript.

R$_f$ = 0.57 (*n*-hexane/ethyl acetate, 1:1, *v*/*v*). **^{1}H NMR** (400 MHz, $CDCl_3$): δ = 7.76 (d, *J* = 7.5 Hz, 2H), 7.60 (d, *J* = 1.1 Hz, 2H), 7.42 – 7.30 (m, 4H), 4.63 (d, *J* = 5.0 Hz, 2H), 4.26 (t, *J* = 5.0 Hz, 1H), 3.17 (s, 4H), 2.42 (s, 4H), 1.87 (s, 4H) ppm. **^{13}C NMR** (101 MHz, $CDCl_3$): δ = 205.5, 157.5, 144.1, 141.6, 127.8, 127.3, 124.8, 120.0, 67.5, 48.5, 47.4, 35.1, 25.7 ppm. **IR** (film): ṽ = 3445 (w, br), 3019 (w), 2926 (w), 2850 (w), 1699 (m), 1478 (w), 1450 (w), 1418 (w), 1341 (w), 1321 (w), 1263 (w), 1216 (w), 1181 (w), 1129 (w), 1051 (w), 1035 (w), 757 (m),

667 (w), 621 (w), 485 (w) cm^{-1}. **UV/Vis** (DCM): λ_{max} = 301, 290, 266, 228 nm. **HR-ESI-MS** (MeOH) m/z: $C_{23}H_{23}NO_4$ calc.: 432.1781 [M+Na+CH$_3$OH]$^+$, meas.: 432.1799.

(9*H*-Fluoren-9-yl)methyl (5*E*,6*E*)-5,6-dihydrazineylideneazonane-1-carboxylate (**10**)

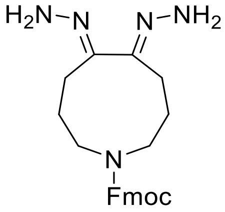

Synthesis of **10** has been described in the main part of the manuscript.

R$_f$ = 0.17 (ethyl acetate + 1 % NEt$_3$). **^{1}H NMR** (400 MHz, CDCl$_3$): δ = 7.80 – 7.71 (m, 2H), 7.62 – 7.54 (m, 2H), 7.43 – 7.27 (m, 4H), 5.26 (s, 4H), 4.56 (d, *J* = 5.2 Hz, 2H), 4.28 – 4.15 (m, 1H), 3.32 – 1.43 (m, 12H) ppm. **^{13}C NMR** (101 MHz, CDCl$_3$): δ = 157.2, 144.4, 141.7, 127.8, 127.2, 124.8, 120.1, 66.6, 47.6, 29.8, 23.1, 22.4 ppm. **HR-ESI-MS** (MeOH) m/z: $C_{23}H_{27}N_5O_2$ calc.: 406.2238 [M+H]$^+$, meas.: 406.2250.

(9*H*-Fluoren-9-yl)methyl 5,6-didehydro-2,3,4,7,8,9-hexahydro-1*H*-azonine-1-carboxylate (**1**)

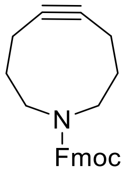

Synthesis of **1** has been described in the main part of the manuscript.

R$_f$ = 0.34 (*n*-hexane/acetone, 5:1, *v*/*v*). **^{1}H NMR** (400 MHz, CD$_3$CN): δ = 7.81 (d, *J* = 7.6 Hz, 2H), 7.63 (d, *J* = 7.5 Hz, 2H), 7.43 – 7.30 (m, 4H), 4.52 (d, *J* = 5.2 Hz, 2H), 4.26 (t, *J* = 5.2 Hz, 1H), 3.25 – 3.14 (m, 2H), 3.04 – 2.96 (m, 2H), 2.13 – 2.08 (m, 2H), 2.02 – 1.96 (m, 2H), 1.76 – 1.67 (m, 2H), 1.34 – 1.25 (m, 2H) ppm. **^{13}C NMR** (101 MHz, CD$_3$CN): δ = 157.1, 145.6, 142.4, 128.5, 128.0, 125.7, 120.9, 85.43, 85.35, 66.9, 54.3, 53.3, 48.4, 29.5, 28.9, 18.82, 18.80 ppm. **IR** (film): ṽ = 3445 (m, br), 3066 (m), 3009 (m), 2925 (s), 2850 (s), 1694 (s), 1475 (s), 1462 (s), 1449 (s), 1416 (s), 1374 (m), 1354 (s), 1343 (m), 1299 (m), 1264 (s), 1205 (s), 1186 (m), 1155 (m), 1120 (s), 1047 (m), 1026 (m), 758 (s), 741 (s), 667 (m) cm^{-1}. **UV/Vis** (DCM): λ_{max} = 301, 290, 266, 228 nm. **HR-ESI-MS** (MeOH) m/z: $C_{23}H_{23}NO_2$ calc.: 368.1621 [M+Na]$^+$, meas.: 368.1625.

(4'-Chloro-[1,1'-biphenyl]-2-yl)-5,6-didehydro-2,3,4,7,8,9-hexahydro-1*H*-azonin-1-yl-methanone (**12a**)

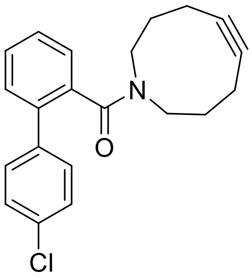

To a solution of 32.6 mg (94.4 µmol, 1.0 equiv.) **1** in DCM (0.50 mL) at 0 °C were added 14.1 µL (14.4 mg, 94.4 µmol, 1.0 equiv.) 1,8-diazabicyclo[5.4.0]undec-7-en and the mixture was stirred for 1.5 h at 0 °C. A solution of 24.2 mg (104 µmol, 1.1 equiv.) 4'-chloro-[1,1'-biphenyl]-2-carboxylic acid (**14**) in DCM (0.71 mL) was prepared. 20.7 mg (153 µmol, 1.6 equiv.) 1-hydroxybenzotriazole hydrate and 27.1 mg (142 µmol, 1.5 equiv.) *N*-(3-dimethylaminopropyl)-*N′*-ethylcarbodiimide hydrochloride were added at 0 °C. Subsequently the deprotected amine prepared above and 39.2 µL (28.6 mg, 283 µmol, 3.0 equiv.) triethylamine were added to the mixture. The reaction was stirred for 5 h, whilst allowing the mixture to warm to room temperature. Water (5 mL) was added, the phases were separated and the aqueous layer was extracted with DCM (3 x 20 mL). The combined organic layers were dried over Na_2SO_4, adsorbed onto silica and purified by column chromatography (*n*-hexane/ethyl acetate, 20:1 to 10:1, *v*/*v*) to give the desired product as a colorless oil. **Yield**: 18.3 mg (54.2 µmol, 57 %). **R_f** = 0.22 (*n*-hexane/ethyl acetate, 3:1, *v*/*v*). **^{1}H NMR** (400 MHz, $CDCl_3$): δ = 7.52 – 7.33 (m, 8H), 3.83 (s, 1H), 3.20 (s, 1H), 2.90 (s, 1H), 2.78 (s, 1H), 2.23 – 1.88 (m, 6H), 1.43 – 1.27 (m, 2H) ppm. **^{13}C NMR** (101 MHz, $CDCl_3$): δ = 172.1, 138.6, 137.1, 136.6, 134.1, 130.3, 129.6, 129.1, 128.8, 128.1, 127.9, 86.0, 84.9, 53.4, 52.6, 30.2, 28.6, 18.9, 18.7 ppm. **IR** (KBr): ṽ = 3050 (m), 2960 (m), 2950 (m), 2918 (s), 2844 (m), 1626 (s), 1496 (m), 1464 (s), 1426 (m), 1415 (s), 1367 (s), 1296 (m), 1281 (m), 1152 (m), 1103 (m), 1088 (s), 1032 (m), 1006 (m), 849 (m), 835 (m), 778 (m), 768 (s), 746 (m), 551 (m), 525 (m), 419 (m) cm^{-1}. **UV/Vis** (DCM): λ_{max} = 248 nm. **HR-ESI-MS** (MeOH) m/z: $C_{21}H_{20}ClNO$ calc.: 338.1306 $[M+Na]^+$, meas.: 338.1309.

(4'-Chloro-[1,1'-biphenyl]-2-yl)methyl 5,6-didehydro-2,3,4,7,8,9-hexahydro-1*H*-azonine-1-carboxylate (**12c**)

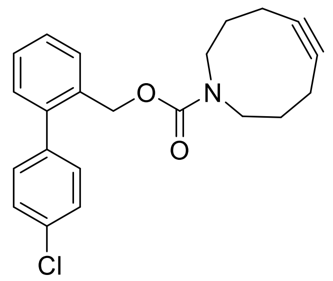

To a solution of 26.6 mg (85.7 µmol, 1.0 equiv.) **1** in DCM (0.44 mL) at 0 °C were added 12.8 µL (13.0 mg, 85.7 µmol, 1.0 equiv.) 1,8-diazabicyclo[5.4.0]undec-7-en and the mixture was stirred at 0 °C for 1 h. Subsequently 75.4 mg (231 µmol, 2.7 equiv.) Cs_2CO_3, 42.2 mg (129 µmol, 1.5 equiv.) 4'-chloro-2-(iodomethyl)-1,1'-biphenyl (**15**) and DMF (0.26 mL) were added and the reaction mixture was stirred at 0 °C for an additional hour. Water (2 mL) and DCM (5 mL) were added and it was extracted with DCM (3 x 20 mL). The combined organic layers were dried over Na_2SO_4, adsorbed onto silica and purified by column chromatography (*n*-hexane/ethyl acetate, 20:1 to 10:1, *v*/*v*) to give the desired product as a colorless oil. **Yield**: 13.2 mg (35.9 µmol, 42 %). **R_f** = 0.41 (*n*-hexane/ethyl acetate, 5:1, *v*/*v*). **^{1}H NMR** (300 MHz, CD_2Cl_2): δ = 7.57 – 7.49 (m, 1H), 7.44 – 7.24 (m, 7H), 5.04 (s, 2H), 3.36 – 3.24 (m, 4H), 2.23 – 2.12 (m, 4H), 1.90 – 1.74 (m, 4H) ppm. **^{13}C NMR** (101 MHz, CD_2Cl_2): δ = 156.6, 141.1, 139.6, 134.9, 133.6, 131.0, 130.3, 129.8, 128.7, 128.4, 128.2, 85.1, 84.7, 64.9, 54.3, 53.1, 29.8, 28.6, 19.0, 18.8 ppm. **IR** (KBr): ṽ = 3446 (w, br), 3011 (w), 2968 (w), 2925 (m), 2849 (w), 1696 (s), 1469 (m), 1415 (m), 1355 (m), 1343 (m), 1263 (m), 1204 (m), 1156 (w), 1121 (m), 1114 (m), 1091 (m), 1020 (m), 1007 (m), 836 (m), 759 (s) cm^{-1}. **UV/Vis** (DCM): λ_{max} = 243 nm. **HR-ESI-MS** (MeOH) m/z: $C_{22}H_{22}ClNO_2$ calc.: 390.1231 $[M+Na]^+$, meas.: 390.1244.

(*R*)-2-Azido-*N*-((4-((4-(dimethylamino)-1-(phenylthio)butan-2-yl)amino)-3-nitrophenyl)sulfonyl)acetamide (**13**)

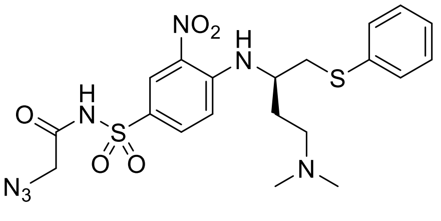

The azide **13** was synthesized as previously described.[4]

(*R*)-2-(7-(4'-Chloro-[1,1'-biphenyl]-2-carbonyl)-5,6,7,8,9,10-hexahydro-[1,2,3]triazolo-[4,5-e]azonin-1(4*H*)-yl)-*N*-((4-((4-(dimethylamino)-1-(phenylthio)butan-2-yl)amino)-3-nitrophenyl)sulfonyl)acetamide (**11a**)

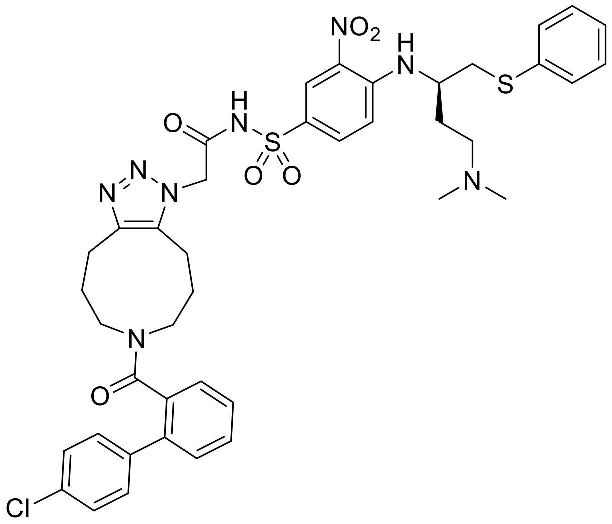

22.4 mg (44.1 µmol, 0.9 equiv.) azide **13** were added to a solution of 14.9 mg (44.1 µmol, 1.0 equiv.) cycloalkyne **12a** in methanol (0.5 mL) and acetonitrile (0.5 mL). The reaction was stirred at 40 °C for 20 days. The solvent was removed *in vacuo*. The residue was adsorbed onto silica and purified by column chromatography (DCM/MeOH, 4:1, *v*/*v*) and reverse phase column chromatography (acetonitrile/water, 3:7 to 4:1, *v*/*v*). Lyophilization gave the desired product as a yellow solid. **Yield**: 26.8 mg (31.7 µmol, 72 %). $\mathbf{R_f}$ (RP) = 0.15 (acetonitrile/water + 1 % TFA, 1:1, *v*/*v*). **m.p.**: 156 – 158 °C. **^{1}H NMR** (400 MHz, CD_2Cl_2): δ = 8.44 (s, 1H), 8.39 – 8.26 (m, 1H), 7.78 – 7.10 (m, 13H), 6.86 – 6.63 (m, 2H), 4.83 (s, 2H), 3.99 (s, 1H), 3.67 – 1.53 (m, 25H) ppm. **^{13}C NMR** (101 MHz, CD_2Cl_2): δ = 173.3, 172.7, 172.2, 145.8, 144.4, 143.5, 138.9, 138.8, 136.9, 136.8, 136.7, 136.6, 135.3, 134.1, 133.9, 133.4, 131.6, 131.2, 131.1, 130.5, 130.4, 129.7, 129.6, 129.5, 129.0, 128.4, 128.3, 127.60, 127.56, 126.2, 114.7, 55.4, 51.5, 50.9, 50.2, 44.2, 39.0, 29.3, 26.7, 26.6, 25.1, 24.7, 23.8, 23.7, 21.4, 21.2 ppm. **IR** (KBr): ṽ = 3060 (w), 2926 (w), 2855 (w), 1616 (m), 1515 (w), 1480 (w), 1470 (w), 1439 (w), 1423 (w), 1358 (w), 1265 (w), 1144 (w), 1092 (w), 1005 (w), 904 (w), 838 (w), 765 (w), 744 (w), 693 (w), 664 (w), 563 (w) cm^{-1}. **UV/Vis** (DCM): λ_{max} = 419, 255 nm. **HR-ESI-MS** (MeOH) m/z: $C_{41}H_{45}ClN_8O_6S_2$ calc.: 845.2665 $[M+H]^+$, meas.: 845.2685.

(4'-Chloro-[1,1'-biphenyl]-2-yl)methyl (*R*)-1-(2-((4-((4-(dimethylamino)-1-(phenylthio)-butan-2-yl)amino)-3-nitrophenyl)sulfonamido)-2-oxoethyl)-4,5,6,8,9,10-hexahydro-[1,2,3]triazolo[4,5-e]azonine-7(1*H*)-carboxylate (**11c**)

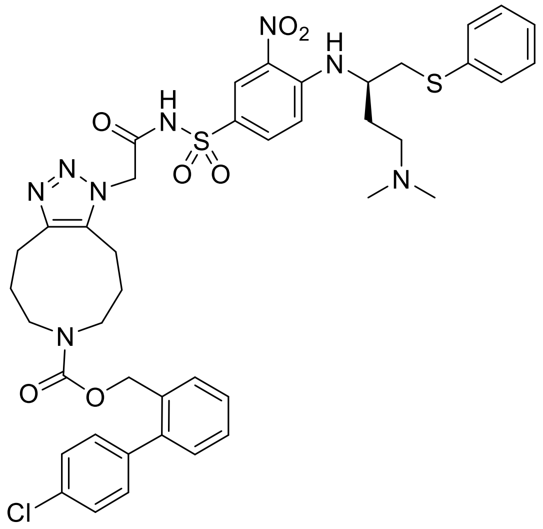

15.7 mg (30.8 µmol, 0.9 equiv.) azide **13** were added to a solution of 11.1 mg (34.3 µmol, 1.0 equiv.) cycloalkyne **12c** in methanol (0.5 mL) and acetonitrile (0.5 mL). The reaction was stirred at 40 °C for 14 days. The solvent was removed *in vacuo*. The residue was adsorbed onto silica and purified by reverse phase column chromatography (acetonitrile/water, 3:7 to 4:1, *v*/*v*). Lyophilization gave the desired product as a yellow solid. **Yield**: 13.2 mg (15.1 µmol, 44 %). $\mathbf{R_f}$ (RP) = 0.24 (acetonitrile/water + 1 % TFA, 1:1, *v*/*v*). **m.p.**: 125 – 129 °C. ^{1}**H NMR** (400 MHz, CD_2Cl_2): δ = 8.41 (s, 1H), 8.33 (d, *J* = 8.4 Hz, 1H), 7.66 (d, *J* = 10.4 Hz, 1H), 7.46 – 7.32 (m, 7H), 7.30 – 7.17 (m, 6H), 6.73 (d, *J* = 9.1 Hz, 1H), 4.94 (s, 1H), 4.88 (s, 1H), 4.80 (s, 1H), 4.74 (s, 1H), 4.00 (s, 1H), 3.25 – 2.84 (m, 9H), 2.66 (s, 10H), 2.24 – 1.71 (m, 6H) ppm. ^{13}**C NMR** (101 MHz, CD_2Cl_2): δ = 173.5, 156.9, 145.8, 144.3, 141.0, 139.5, 135.3, 134.4, 134.2, 134.0, 133.6, 131.4, 131.2, 131.0, 130.9, 130.3, 129.9, 129.7, 128.8, 128.5, 128.3, 127.6, 126.3, 114.6, 65.1, 55.4, 53.2, 51.0, 44.2, 39.0, 30.1, 29.3, 27.6, 25.0, 22.4, 20.0 ppm. **IR** (KBr): ṽ = 3440 (m, br), 2925 (w), 1697 (m), 1615 (m), 1513 (w), 1479 (w), 1448 (w), 1438 (w), 1420 (w), 1354 (w), 1278 (m), 1264 (m), 1230 (w), 1141 (w), 1092 (w), 765 (w), 745 (w), 566 (w), 559 (w) cm^{-1}. **UV/Vis** (DCM): λ_{max} = 417, 257, 230 nm. **HR-ESI-MS** (MeOH) m/z: $C_{42}H_{47}ClN_8O_7S_2$ calc.: 875.2798 $[M+H]^+$, meas.: 875.2771.

(9*H*-Fluoren-9-yl)methyl 1-benzyl-4,5,6,8,9,10-hexahydro-[1,2,3]triazolo[4,5-e]azonine-7(1*H*)-carboxylate (**S1**)

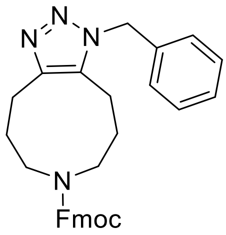

To a solution of 10.4 mg (30.1 µmol, 1.0 equiv.) **1** in acetonitrile (0.60 mL) were added 21.3 µL (20.0 mg, 150 µmol, 5.0 equiv.) benzyl azide and the mixture was stirred at 40 °C for 5 days. The residue was purified by column chromatography (DCM/methanol, 1:0 to 20:1, *v*/*v*) to give the desired product as a colorless oil. **Yield**: 11.3 mg (23.6 µmol, 79 %). $\mathbf{R_f}$ = 0.37 (DCM/methanol, 20:1, *v*/*v*). ^{1}**H NMR** (400 MHz, CD_3CN): δ = 7.85 – 7.76 (m, 2H), 7.61 (d, *J* = 7.4 Hz, 2H), 7.44 – 7.25 (m, 7H), 7.19 – 7.05 (m, 2H), 5.41 (s, 1H), 5.25 (s, 1H), 4.56 – 4.39 (m, 2H), 4.24 – 4.15 (m, 1H), 2.97 – 2.76 (m, 3H), 2.61 – 2.38 (m, 4H), 2.30 – 2.21 (m, 1H), 1.91 – 1.84 (m, 1H), 1.83 – 1.71 (m, 1H), 1.60 – 1.47 (m, 1H), 1.23 – 1.12 (m, 1H) ppm.
^{1}**H NMR** (300 MHz, CD_3CN, **T = 70 °C**) δ = 7.85 – 7.78 (m, 2H), 7.65 – 7.59 (m, 2H), 7.46 – 7.29 (m, 7H), 7.19 – 7.11 (m, 2H), 5.35 (s, 2H), 4.48 (d, J = 5.1 Hz, 2H), 4.21 (t, J = 5.1 Hz, 1H), 3.00 – 2.69 (m, 4H), 2.64 – 2.35 (m, 4H), 1.89 – 1.43 (m, 4H) ppm. ^{13}**C NMR** (101 MHz, CD_3CN): δ = 157.2, 157.0, 145.42, 145.35, 142.3, 137.2, 133.5, 129.7, 128.9, 128.5, 128.0, 125.8, 125.7, 120.9, 66.5, 66.2, 52.6, 52.2, 52.0, 51.4, 51.3, 48.5, 27.5, 26.9, 25.7, 25.1, 22.4, 20.0, 19.6 ppm. **IR** (film): ṽ = 3476 (w, br), 3065 (w), 3010 (w), 2925 (m), 2852 (m), 1697 (s), 1483 (s), 1450 (m), 1418 (m), 1378 (m), 1350 (m), 1312 (m), 1288 (m), 1220 (m), 1180 (m), 1160 (m), 1121 (m), 1032 (m), 984 (w), 758 (s), 743 (s), 665 (w) cm^{-1}. **UV/Vis** (DCM): λ_{max} = 301, 290, 267, 229 nm. **HR-ESI-MS** (MeOH) m/z: $C_{30}H_{30}N_4O_2$ calc.: 501.2261 $[M+Na]^+$, meas.: 501.2275.

(*Z*)-1-((4'-Chloro-[1,1'-biphenyl]-2-yl)methyl)-2,3,4,7,8,9-hexahydro-1*H*-azonine (**S2**)

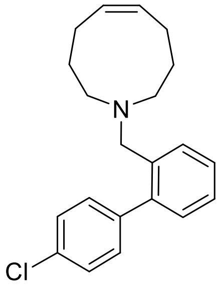

To a solution of 30.0 mg (86.3 µmol, 1.0 equiv.) azacyclononene **8** in DCM (0.44 mL) at 0 °C were added 12.9 µL (13.1 mg, 86.3 µmol, 1.0 equiv.) 1,8-diazabicyclo[5.4.0]undec-7-en and the mixture was stirred at 0 °C for 1 h. Subsequently 56.3 mg (173 µmol, 2.0 equiv.) Cs_2CO_3,

31.2 mg (95.0 µmol, 1.1 equiv.) 4'-chloro-2-(iodomethyl)-1,1'-biphenyl (**15**) and DMF (0.26 mL) were added and the reaction mixture was stirred at 0 °C for an additional hour. Water (2 mL) and DCM (5 mL) were added and it was extracted with DCM (3 x 20 mL). The combined organic layers were dried over Na_2SO_4, adsorbed onto silica and purified by column chromatography (*n*-hexane/ethyl acetate, 100:1 to 50:1, *v*/*v*) to give the desired product as a colorless oil, which solidified upon standing. **Yield**: 10.3 mg (31.6 µmol, 37 %). **R_f** = 0.55 (*n*-hexane/ethyl acetate, 10:1, *v*/*v*). **m.p.**: 82 – 85 °C. **^{1}H NMR** (300 MHz, CD_2Cl_2): δ = 7.67 – 7.62 (m, 1H), 7.41 – 7.25 (m, 6H), 7.20 – 7.15 (m, 1H), 5.47 – 5.35 (m, 2H), 3.53 (s, 2H), 2.30 (t, *J* = 6.2 Hz, 4H), 2.18 – 2.08 (m, 4H), 1.37 – 1.27 (m, 4H) ppm. **^{13}C NMR** (75 MHz, CD_2Cl_2): δ = 141.5, 140.7, 138.1, 133.1, 131.2, 130.8, 130.6, 130.2, 128.5, 127.8, 126.9, 57.3, 50.7, 25.6, 22.5 ppm. **IR** (KBr): ṽ = 3444 (m, br), 3056 (w), 3017 (w), 2959 (m), 2945 (m), 2922 (s), 2852 (m), 2790 (m), 1473 (m), 1446 (m), 1372 (m), 1276 (w), 1239 (w), 1142 (w), 1119 (w), 1087 (m), 1017 (m), 1006 (m), 837 (m), 759 (s), 733 (m), 715 (m), 553 (w) cm^{-1}. **UV/Vis** (DCM): λ_{max} = 235 nm. **HR-ESI-MS** (MeOH) m/z: $C_{21}H_{24}ClN$ calc.: 326.1670 $[M+H]^+$, meas.: 326.1679.

1-(*Tert*-butyl) 3-ethyl 4-oxopiperidine-1,3-dicarboxylate (**S4**) [5]

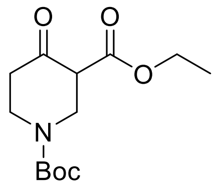

To a solution of 5.27 g (25.4 mmol, 1.0 equiv.) ethyl 4-oxopiperidine-3-carboxylate hydrochloride (**S3**) in DCM (51 mL) were added 7.4 mL (5.39 g, 53.3 mmol, 2.1 equiv.) triethylamine and 6.65 g (30.4 mmol, 1.2 equiv.) di-*tert*-butyl dicarbonate. The mixture was stirred at room temperature for 16 h. Saturated NH_4Cl solution (100 mL) was added and it was extracted with DCM (3 x 50 mL). The combined organic layers were dried over Na_2SO_4 and purified by column chromatography (*n*-hexane/ethyl acetate, 10:1 to 5:1, *v*/*v*) to give the desired product as a colorless oil, which solidified upon standing. **Yield**: 6.89 g (25.4 mmol, quant.). **R_f** = 0.45 (*n*-hexane/ethyl acetate, 5:1, *v*/*v*). **m.p.**: 63 – 65 °C. **^{1}H NMR** (300 MHz, $CDCl_3$): δ = 12.04 (s, 1H), 4.23 (q, *J* = 7.1 Hz, 2H), 4.05 (s, 2H), 3.55 (t, *J* = 5.9 Hz, 2H), 2.40 – 2.31 (m, 2H), 1.47 (s, 9H), 1.29 (t, *J* = 7.1 Hz, 3H) ppm. **^{13}C NMR** (75 MHz, $CDCl_3$): δ = 202.9, 170.9, 168.1, 154.7, 154.4, 81.0, 80.2, 61.7, 60.7, 56.6, 40.8, 40.4, 29.0, 28.5, 28.4, 14.3, 14.2 ppm. **IR** (KBr): ṽ = 2978 (m), 2936 (m), 2914 (w), 2870 (w), 1691 (s), 1623 (m), 1476 (m), 1409 (s), 1366 (s), 1310 (s), 1252 (s), 1231 (s), 1201 (s), 1170 (s), 1119 (m), 1065 (s), 1011 (m), 910 (m), 877 (m), 829 (m), 774 (m), 737 (w), 566 (w), 461 (w) cm^{-1}. **UV/Vis** (DCM): λ_{max} = 267 nm. **HR-ESI-MS** (MeOH) m/z: $C_{13}H_{21}NO_5$ calc.: 326.1574 $[M+Na+CH_3OH]^+$, meas.: 326.1581.

1-(*Tert*-butyl) 3-ethyl 4-(((trifluoromethyl)sulfonyl)oxy)-5,6-dihydropyridine-1,3(2*H*)-dicarboxylate (**S5**) [6]

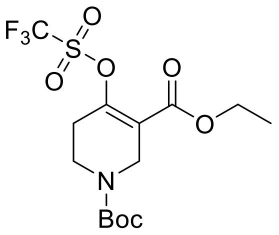

To a solution of 3.01 g (11.1 mmol, 1.0 equiv.) **S4** in DMF (75.5 mL) were added at 0 °C 638 mg (60 wt. % dispersion in mineral oil, 26.6 mmol, 1.5 equiv.) sodium hydride. After stirring for 10 min, 5.15 g (14.4 mmol, 1.3 equiv.) 1,1,1-trifluoro-*N*-phenyl-*N*-((trifluoromethyl)sulfonyl)-methanesulfonamide in DMF (7.8 mL) were added dropwise at 0 °C. The mixture was stirred for 18 h, whilst allowing the mixture to warm to room temperature. Water (100 mL) was added and it was extracted with ethyl acetate (3 x 50 mL). The combined organic layers were dried over Na_2SO_4 and purified by column chromatography (*n*-hexane/acetone, 10:1, *v*/*v*) to give the desired product as a pale yellow oil. **Yield**: 3.75 g (9.29 mmol, 84 %). $\mathbf{R_f}$ = 0.47 (*n*-hexane/acetone, 5:1, *v*/*v*). **^{1}H NMR** (400 MHz, $CDCl_3$): δ = 4.37 – 4.20 (m, 4H), 3.62 (t, J = 5.7 Hz, 2H), 2.55 – 2.46 (m, 2H), 1.48 (s, 9H), 1.33 (t, J = 7.1 Hz, 3H) ppm. **^{19}F NMR** (377 MHz, $CDCl_3$): δ = -74.6 (s) ppm. **^{13}C NMR** (76 MHz, $CDCl_3$): δ = 162.6, 154.2, 150.4, 121.1, 118.4 (q, J = 322.1 Hz), 81.2, 62.1, 43.2, 39.7, 29.0, 28.5, 14.1 ppm. **IR** (film): ṽ = 2983 (m), 1715 (s), 1588 (m), 1426 (s), 1371 (m), 1294 (m), 1248 (s), 1213 (s), 1162 (s), 1141 (s), 1080 (m), 1045 (m), 1031 (m), 910 (m), 872 (m), 828 (m), 768 (m), 639 (m), 620 (m), 595 (m) cm^{-1}. **UV/Vis** (DCM): λ_{max} = 293, 228 nm. **HR-ESI-MS** (MeOH) m/z: $C_{14}H_{20}F_3NO_7S$ calc.: 426.0805 $[M+Na]^+$, meas.: 426.0813.

1-(*Tert*-butyl) 3-ethyl 4-(4-chlorophenyl)-5,6-dihydropyridine-1,3(2*H*)-dicarboxylate (**S6**)

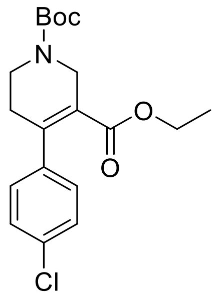

3.70 g (9.18 mmol, 1.0 equiv.) **S5**, 1.72 g (11.0 mmol, 1.2 equiv.) 4-chlorophenyl boronic acid and 1.94 g (18.4 mmol, 2.0 equiv.) Na_2CO_3 were suspended in toluene (55 mL) and ethanol (23 mL). The mixture was degassed by sonication (10x). 1.06 g (918 µmol, 0.1 equiv.) $Pd(PPh_3)_4$ were added and the reaction was stirred at 80 °C for 16 h. After cooling to room temperature, it was filtered over Celite® 545 and washed with ethyl acetate (3 x 50 mL). The solvent was removed *in vacuo* and the crude product was adsorbed onto silica and purified by

column chromatography (*n*-hexane/acetone, 1:0 to 10:1, *v*/*v*) to give the desired product as a colorless oil.[7] **Yield**: 3.28 g (8.97 mmol, 98 %). **R_f** = 0.47 (*n*-hexane/acetone, 5:1, *v*/*v*). **^{1}H NMR** (400 MHz, $CDCl_3$): δ = 7.32 – 7.28 (m, 2H), 7.09 – 7.04 (m, 2H), 4.24 (s, 2H), 3.96 (q, J = 7.1 Hz, 2H), 3.60 (t, J = 5.7 Hz, 2H), 2.50 – 2.42 (m, 2H), 1.50 (s, 9H), 0.97 (t, J = 5.8 Hz, 3H) ppm. **^{13}C NMR** (101 MHz, $CDCl_3$): δ = 166.7, 154.7, 145.2, 140.5, 133.5, 128.5, 128.3, 125.7, 80.4, 60.6, 43.9, 39.7, 33.0, 28.6, 13.8 ppm. **IR** (film): ṽ = 2979 (s), 2931 (m), 2901 (m), 2846 (m), 1705 (s), 1593 (m), 1491 (s), 1393 (s), 1366 (s), 1335 (m), 1296 (s), 1239 (m), 1169 (m), 1136 (m), 1115 (m), 1092 (s), 1053 (s), 1015 (s), 990 (m), 897 (m), 863 (m), 826 (s), 767 (s), 733 (m), 532 (m) cm^{-1}. **UV/Vis** (DCM): λ_{max} = 246, 231 nm. **HR-ESI-MS** (MeOH) m/z: $C_{19}H_{24}ClNO_4$ calc.: 388.1286 $[M+Na]^+$, meas.: 388.1287.

<u>*Tert*-butyl 4-(4-chlorophenyl)-3-(hydroxymethyl)- 1,2,5,6-tetrahydropyridine-1(2*H*)-carboxylate **(S7)**</u>

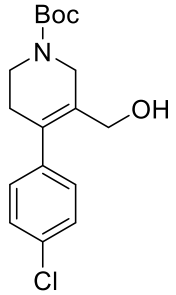

7.13 mL (271 mg, 7.13 mmol, 0.8 equiv.) 1M lithium aluminum hydride solution in THF were added dropwise at -20 °C to a solution of 3.26 g (8.91 mmol, 1.0 equiv.) **S6** in THF (65 mL). The mixture was stirred at -20 °C for 1 h. Saturated NH_4Cl solution (100 mL) was added slowly and it was extracted with ethyl acetate (3 x 50 mL). The combined organic layers were dried over Na_2SO_4 and purified by column chromatography (*n*-hexane/acetone, 20:1 to 5:1, *v*/*v*) to give the desired product as a colorless oil.[7] **Yield**: 2.51 g (7.76 mmol, 87 %). **R_f** = 0.37 (*n*-hexane/acetone, 3:1, *v*/*v*). **^{1}H NMR** (400 MHz, $CDCl_3$): δ = 7.33 – 7.29 (m, 2H), 7.14 – 7.09 (m, 2H), 4.11 (s, 2H), 4.01 (s, 2H), 3.59 (t, J = 5.8 Hz, 2H), 2.38 (t, J = 2.8 Hz, 2H), 1.49 (s, 9H) ppm. **^{13}C NMR** (101 MHz, $CDCl_3$): δ = 155.0, 139.5, 134.4, 133.2, 131.0, 129.5, 128.7, 80.0, 61.6, 44.8, 41.3, 31.6, 28.6 ppm. **IR** (film): ṽ = 3421 (m), 3006 (m), 2978 (s), 2929 (m), 2869 (m), 1677 (s), 1491 (s), 1477 (s), 1429 (s), 1366 (s), 1336 (m), 1292 (s), 1244 (s), 1168 (s), 1128 (s), 1092 (s), 1046 (m), 1014 (s), 976 (m), 893 (m), 859 (m), 825 (m), 757 (s), 665 (m), 450 (m) cm^{-1}. **UV/Vis** (DCM): λ_{max} = 240 nm. **HR-ESI-MS** (MeOH) m/z: $C_{17}H_{22}ClNO_3$ calc.: 346.1180 $[M+Na]^+$, meas.: 346.1180.

Tert-butyl 4-(4-chlorophenyl)-3-(iodomethyl)- 1,2,5,6-tetrahydropyridine-1(2H)-carboxylate (**16**)

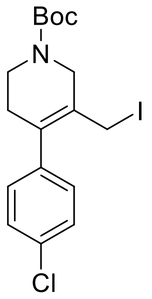

To a stirred solution of 52.0 mg (161 µmol, 1.0 equiv.) **S7** in THF (0.37 mL) were added at 0 °C 50.5 mg (193 µmol, 1.2 equiv.) triphenylphosphane and 13.1 mg (193 µmol, 1.2 equiv.) 1*H*-imidazole. A solution of 48.9 mg (193 µmol, 1.2 equiv.) I_2 in THF (0.13 mL) was added at 0 °C. The reaction mixture was stirred for 1 h at this temperature and subsequently quenched by the addition of a saturated $Na_2S_2O_3$ solution (5 mL). It was extracted with ethyl acetate (3 x 10 mL). The combined organic layers were dried over Na_2SO_4, adsorbed onto silica and purified by column chromatography (*n*-hexane/ethyl acetate, 1:0 to 20:1, *v*/*v*) to give the desired product as a yellow oil. **Yield**: 47.1 mg (109 µmol, 68 %). $\mathbf{R_f}$ = 0.70 (*n*-hexane/ethyl acetate, 3:1, *v*/*v*). ^{1}**H NMR** (400 MHz, $CDCl_3$): δ = 7.40 – 7.35 (m, 2H), 7.25 – 7.20 (m, 2H), 4.21 (s, 2H), 3.79 (s, 2H), 3.59 (t, J = 5.7 Hz, 2H), 2.25 (t, J = 2.7 Hz, 2H), 1.51 (s, 9H) ppm. ^{13}**C NMR** (101 MHz, $CDCl_3$): δ = 154.8, 139.5, 133.5, 129.1, 129.0, 128.7, 128.4, 80.2, 45.5, 40.6, 32.4, 28.6, 5.9 ppm. **IR** (film): ṽ = 2974 (w), 2926 (w), 2854 (w), 1692 (m), 1634 (m), 1595 (w), 1488 (m), 1421 (m), 1393 (w), 1366 (m), 1336 (w), 1244 (w), 1165 (m), 1130 (m), 1092 (m), 1013 (w), 827 (m), 758 (m), 598 (w) cm^{-1}. **UV/Vis** (DCM): λ_{max} = 365, 295, 229 nm. **HR-EI-MS** m/z: $C_{17}H_{21}ClINO_2$ calc.: 375.9596 [M-*t*Bu]$^+$, meas.: 375.9596.

(1-(Tert-butoxycarbonyl)-4-(4-chlorophenyl)-1,2,5,6-tetrahydropyridine-3-yl)methyl 5,6-didehydro-2,3,4,7,8,9-hexahydro-1*H*-azonine-1-carboxylate (**17**)

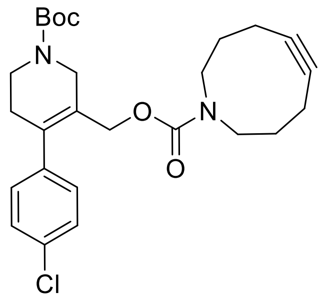

To a solution of 41.7 mg (121 µmol, 1.0 equiv.) **1** in DCM (0.62 mL) at 0 °C were added 18.0 µL (18.4 mg, 121 µmol, 1.0 equiv.) 1,8-diazabicyclo[5.4.0]undec-7-en and the mixture was stirred at 0 °C for 1 h. Subsequently 106 mg (326 µmol, 2.7 equiv.) Cs_2CO_3 and a solution of 78.5 mg (181 µmol, 1.5 equiv.) **16** in DMF (0.36 mL) were added and the reaction mixture was stirred

at 0 °C for an additional hour. Water (2 mL) and DCM (5 mL) were added and it was extracted with DCM (3 x 10 mL). The combined organic layers were dried over Na_2SO_4, adsorbed onto silica and purified by column chromatography (*n*-hexane/ethyl acetate, 20:1 to 10:1, *v*/*v*) to give the desired product as a colorless oil. **Yield**: 34.7 mg (73.4 µmol, 61 %). $\mathbf{R_f}$ = 0.25 (*n*-hexane/ethyl acetate, 3:1, *v*/*v*). ^{1}**H NMR** (400 MHz, CD_2Cl_2): δ = 7.37 – 7.29 (m, 2H), 7.17 – 7.09 (m, 2H), 4.43 (s, 2H), 4.03 (s, 2H), 3.58 (t, J = 5.8 Hz, 2H), 3.37 – 3.28 (m, 4H), 2.44 – 2.34 (m, 2H), 2.25 – 2.14 (m, 4H), 1.94 – 1.79 (m, 4H), 1.46 (s, 9H) ppm. ^{13}**C NMR** (101 MHz, CD_2Cl_2): δ = 156.6, 154.9, 139.9, 136.1, 133.4, 129.9, 128.9, 127.6, 85.2, 84.7, 79.8, 64.0, 54.3, 53.2, 45.2, 31.9, 30.1, 30.0, 28.7, 28.6, 19.0, 18.9 ppm. **IR** (KBr): ṽ = 3436 (m), 2972 (w), 2928 (w), 1696 (s), 1609 (w), 1524 (m), 1491 (w), 1472 (w), 1415 (m), 1352 (m), 1289 (w), 1262 (m), 1242 (m), 1203 (w), 1168 (w), 1127 (m), 1091 (w), 1017 (w), 1006 (w), 825 (w), 770 (w) cm^{-1}. **UV/Vis** (DCM): λ_{max} = 256 nm. **HR-ESI-MS** (MeOH) m/z: $C_{26}H_{33}ClN_2O_4$ calc.: 495.2021 $[M+Na]^+$, meas.: 495.2022.

(4-(4-Chlorophenyl)-1,2,5,6-tetrahydropyridine-3-yl)methyl 5,6-didehydro-2,3,4,7,8,9-hexahydro-1*H*-azonine-1-carboxylate (**12d**)

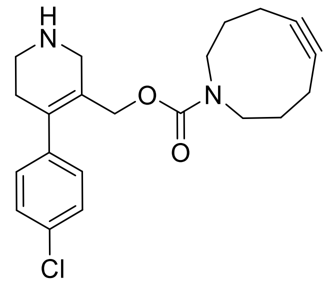

281 µL (418 mg, 3.67 mmol, 50 equiv.) trifluoroacetic acid were added dropwise to a solution of 34.7 mg (73.4 µmol, 1.0 equiv.) **17** in DCM (2.2 mL) at 0 °C. The reaction was stirred for 3 h, whilst allowing the mixture to warm to room temperature. The mixture was adjusted to pH 8 using a saturated $NaHCO_3$ solution and extracted with DCM (3 x 10 mL). The combined organic layers were dried over Na_2SO_4, adsorbed onto silica and purified by column chromatography (DCM/MeOH, 1:0 to 10:1, *v*/*v*) to give the desired product as a colorless oil. **Yield**: 18.7 mg (50.1 µmol, 68 %). $\mathbf{R_f}$ = 0.20 (DCM/MeOH,10:1, *v*/*v*). ^{1}**H NMR** (400 MHz, $CDCl_3$): δ = 7.33 – 7.28 (m, 2H), 7.14 – 7.08 (m, 2H), 4.46 (s, 2H), 3.54 (s, 2H), 3.37 – 3.29 (m, 4H), 3.11 (t, J = 5.8 Hz, 2H), 2.70 (s, 1H), 2.42 – 2.35 (m, 2H), 2.26 – 2.15 (m, 4H), 1.94 – 1.82 (m, 4H) ppm. ^{13}**C NMR** (76 MHz, $CDCl_3$): δ = 156.6, 139.4, 135.9, 133.3, 129.5, 129.2, 128.7, 85.1, 84.5, 64.2, 54.1, 53.0, 46.5, 43.2, 31.5, 29.7, 28.3, 18.9, 18.7 ppm. **IR** (film): ṽ = 3442 (w), 2955 (w), 2921 (m), 2849 (w), 1694 (m), 1491 (w), 1468 (w), 1415 (w), 1359 (w), 1341 (w), 1261 (w), 1202 (w), 1184 (w), 1113 (w), 1092 (w), 1015 (w), 824 (w), 771 (w),

412 (w) cm^{-1}. **UV/Vis** (DCM): λ_{max} = 239 nm. **HR-ESI-MS** (MeOH) m/z: $C_{21}H_{25}ClN_2O_2$ calc.: 373.1678 $[M+H]^+$, meas.: 373.1682.

(4-(4-Chlorophenyl)-1,2,5,6-tetrahydropyridine-3-yl)methyl (*R*)-1-(2-((4-((4-(dimethylamino)-1-(phenylthio)butan-2-yl)amino)-3-nitrophenyl)sulfonamido)-2-oxoethyl)-4,5,6,8,9,10-hexahydro-[1,2,3]triazolo[4,5-e]azonine-7(1*H*)-carboxylate (**11d**)

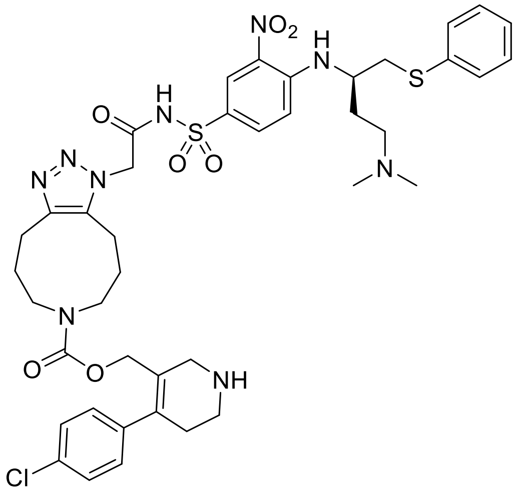

16.5 mg (32.4 µmol, 1.0 equiv.) azide **13** were added to a solution of 12.1 mg (32.4 µmol, 1.0 equiv.) cycloalkyne **12d** in methanol (0.26 mL) and acetonitrile (0.35 mL). The reaction was stirred at 40 °C for 30 days and at room temperature for 30 days. The solvent was removed *in vacuo*. The residue was adsorbed onto silica and purified by reverse phase column chromatography (acetonitrile/water, 1:9 to 1:4 to 3:7 to 1:1 to 7:3 to 4:1, *v*/*v*). Lyophilization gave the desired product as a yellow solid. **Yield**: 24.5 mg (27.8 µmol, 86 %). $\mathbf{R_f}$ (RP) = 0.32 (acetonitrile/water + 1 % TFA, 1:1, *v*/*v*). **m.p.**: 204 – 207 °C. ^{1}**H NMR** (400 MHz, DMSO-d_6): δ = 8.43 (d, J = 8.8 Hz, 1H), 8.35 (d, J = 2.1 Hz, 1H), 7.69 (d, J = 9.1 Hz, 1H), 7.46 (dd, J = 7.1, 4.0 Hz, 2H), 7.30 – 7.21 (m, 7H), 6.85 (d, J = 9.3 Hz, 1H), 4.67 (s, 1H), 4.63 (s, 1H), 4.29 (s, 1H), 4.22 (s, 1H), 4.09 – 3.97 (m, 1H), 3.58 – 3.53 (m, 2H), 3.38 – 3.21 (m, 6H), 3.10 – 2.97 (m, 4H), 2.73 – 2.52 (m, 4H), 2.38 – 2.25 (m, 2H), 2.18 (s, 6H), 2.05 – 1.62 (m, 8H) ppm. ^{13}**C NMR** (101 MHz, DMSO-d_6): δ = 170.3, 170.1, 155.3, 145.4, 142.9, 138.1, 135.4, 134.8, 134.5, 134.3, 133.0, 132.4, 132.3, 129.7, 129.4, 129.0, 128.5, 126.2, 125.7, 125.4, 124.6, 124.4, 113.6, 62.7, 54.9, 52.6, 51.4, 50.9, 50.6, 44.7, 43.0, 40.5, 37.1, 30.4, 27.8, 26.9, 25.2, 23.7, 19.4 ppm. **IR** (KBr): ṽ = 3452 (m, br), 2924 (w), 2851 (w), 1619 (w), 1514 (w), 1482 (w), 1440 (w), 1422 (w), 1384 (w), 1359 (w), 1278 (w), 1232 (w), 1142 (w), 1092 (w), 1065 (w), 1044 (w), 1024 (w), 866 (w), 742 (w), 692 (w), 664 (w), 573 (w), 562 (w), 516 (w) cm^{-1}. **UV/Vis**

(acetonitrile): λ_{max} = 434, 259 nm. **HR-ESI-MS** (MeOH) m/z: $C_{41}H_{50}ClN_9O_7S_2$ calc.: 880.3036 $[M+H]^+$, meas.: 880.3059.

<u>*Tert*-butyl (*Z*)-2,3,4,7,8,9-hexahydro-1*H*-azonine-1-carboxylate (**8a**) [3]</u>

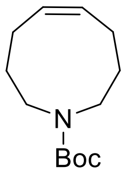

A Schlenk tube was equipped with 723 mg (5.64 mmol, 6.2 equiv.) naphthalene and THF (14.6 mL). 130 mg (5.64 mmol, 6.2 equiv.) sodium were added and the solution was stirred for 20 min at room temperature. The freshly prepared sodium naphthalenide solution was added dropwise to a solution of 254 mg (0.910 mmol, 1.0 equiv.) **7** in THF (3.6 mL) at -78 °C. The reaction mixture was stirred for 20 min at this temperature and subsequently quenched by the addition of a saturated $NaHCO_3$ solution (14 mL). The mixture was extracted with ethyl acetate (6 x 30 mL). The combined organic layers were dried over Na_2SO_4 and the solvent was removed *in vacuo*. The crude product was used directly in the next step without further purification.

To the deprotected amine in DCM (6.4 mL) at 0 °C were added 477 mg (2.81 mmol, 2.4 equiv.) di-*tert*-butyl dicarbonate, 22.2 mg (0.182 mmol, 0.2 equiv.) DMAP and 252 µL (184 mg, 1.82 mmol, 2.0 equiv.) triethylamine. The reaction was stirred for 16 h, whilst allowing the mixture to warm to room temperature. Water (5 mL) and DCM (5 mL) were added and it was extracted with DCM (3 x 30 mL). The combined organic layers were dried over Na_2SO_4 and purified by column chromatography (*n*-hexane/ethyl acetate, 100:1 to 50:1, *v*/*v*) to give the desired product as a colorless oil. **Yield**: 99.2 mg (0.440 mmol, 48 %). $\mathbf{R_f}$ = 0.45 (*n*-hexane/ethyl acetate, 10:1, *v*/*v*). **^{1}H NMR** (400 MHz, $CDCl_3$): δ = 5.55 – 5.42 (m, 2H), 3.11 (dt, *J* = 16.8, 6.3 Hz, 4H), 2.27 – 2.14 (m, 4H), 1.86 (p, *J* = 6.3 Hz, 2H), 1.79 (p, *J* = 6.3 Hz, 2H), 1.46 (s, 9H) ppm. **^{13}C NMR** (101 MHz, $CDCl_3$): δ = 156.5, 130.2, 129.6, 79.0, 52.8, 52.1, 28.7, 27.0, 26.1, 22.8, 22.7 ppm. **IR** (film): $\tilde{\nu}$ = 3004 (m), 2973 (s), 2920 (s), 2858 (m), 1697 (s), 1484 (s), 1460 (m), 1413 (s), 1364 (s), 1352 (s), 1317 (s), 1291 (m), 1255 (m), 1227 (m), 1171 (s), 1115 (m), 1095 (m), 867 (m), 756 (m), 720 (m) cm^{-1}. **UV/Vis** (DCM): λ_{max} = 264, 228 nm. **HR-ESI-MS** (MeOH) m/z: $C_{13}H_{23}NO_2$ calc.: 248.1621 $[M+Na]^+$, meas.: 248.1625.

<u>*Tert*-butyl 5,6-dioxoazonane-1-carboxylate (**9a**)</u>

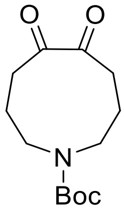

Under ambient atmosphere 84.1 mg (373 µmol, 1.0 equiv.) **8a**, 41.4 mg (112 µmol, 0.3 equiv.) TBAI and 2.29 mg (3.73 µmol, 0.01 equiv.) [Ru(*p*-cymene)Cl_2]$_2$ were dissolved in toluene (0.60 mL), acetonitrile (0.60 mL) and water (0.12 mL). 184 µL (70 wt. % in water, 173 mg, 1.92 mmol, 3.6 equiv.) *tert*-butyl hydroperoxide solution were added and the reaction mixture was stirred for 1 h at 35 °C. The reaction was quenched by the addition of a saturated Na_2SO_3 solution (5 mL) and it was extracted with ethyl acetate (3 x 30 mL). The combined organic layers were dried over Na_2SO_4 and purified by column chromatography (*n*-hexane/ethyl acetate, 10:1 to 5:1, *v*/*v*) to give the desired product as a colorless oil. **Yield**: 66.4 mg (260 µmol, 70 %). $\mathbf{R_f}$ = 0.17 (*n*-hexane/ethyl acetate, 5:1, *v*/*v*). ^{1}**H NMR** (300 MHz, $CDCl_3$): δ = 3.27 (t, J = 6.3 Hz, 4H), 2.69 – 2.60 (m, 4H), 2.14 – 2.02 (m, 4H), 1.49 (s, 9H) ppm. ^{13}**C NMR** (75 MHz, $CDCl_3$): δ = 205.0, 156.9, 81.2, 48.3, 35.1, 28.5, 25.8 ppm. **IR** (film): ṽ = 2978 (w), 2932 (m), 2872 (w), 1698 (s), 1478 (m), 1467 (m), 1406 (m), 1367 (m), 1343 (m), 1274 (m), 1255 (m), 1164 (s), 1130 (m), 1072 (w), 1052 (w), 1028 (w), 887 (w), 864 (w), 776 (w) cm^{-1}. **UV/Vis** (DCM): λ_{max} = 244 nm. **HR-ESI-MS** (MeOH) m/z: $C_{13}H_{21}NO_4$ calc.: 310.1625 $[M+Na+CH_3OH]^+$, meas.: 310.1629.

Tert-butyl (5*E*,6*E*)-5,6-dihydrazineylideneazonane-1-carboxylate (**10a**)

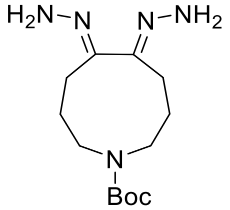

To a solution of 37.4 µL (38.4 mg, 767 µmol, 3.3 equiv.) hydrazine monohydrate in ethanol (0.22 mL) at -20 °C was added a solution of 58.6 mg (230 µmol, 1.0 equiv.) **9a** in ethanol (0.18 mL). The reaction mixture was stirred for 1 h at -20 °C and for 30 min at room temperature. The solvent was removed *in vacuo* to give the desired product as a colorless oil, which was used directly in the next step without further purification. **Yield**: 63.0 mg (222 µmol, 97 %). $\mathbf{R_f}$ = 0.12 (ethyl acetate + 1 % NEt_3). ^{1}**H NMR** (400 MHz, $CDCl_3$): δ = 5.42 (s, 4H), 3.35 – 2.90 (m, 4H), 2.69 – 2.40 (m, 4H), 2.13 – 1.85 (m, 4H), 1.44 (s, 9H) ppm. ^{13}**C NMR** (101 MHz, $CDCl_3$): δ = 156.7, 79.6, 50.3, 28.60, 28.56, 22.8 ppm. **HR-ESI-MS** (MeOH) m/z: $C_{13}H_{25}N_5O_2$ calc.: 306.1900 $[M+Na]^+$, meas.: 306.1903.

Tert-butyl 5,6-didehydro-2,3,4,7,8,9-hexahydro-1*H*-azonine-1-carboxylate (**1a**)

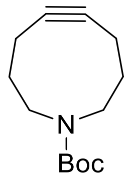

63.0 mg (222 µmol, 1.0 equiv.) **10a** were dissolved in DCM (0.5 mL) and cooled to -20 °C. A solution of 212 mg (478 µmol, 2.1 equiv.) lead tetraacetate (*Caution!* Lead tetraacetate is highly toxic and potentially carcinogenic, requiring additional safety precautions before handling!) in DCM (0.5 mL) was added slowly over a period of 15 min and the mixture was stirred for 1.5 h at -20 °C. The reaction was quenched by the addition of a saturated $NaHCO_3$ solution (5 mL) and it was extracted with DCM (3 x 30 mL). The combined organic layers were dried over Na_2SO_4, adsorbed onto silica and purified by column chromatography (*n*-hexane/ethyl acetate, 50:1 to 10:1, *v*/*v*) to give the desired product as a colorless oil. **Yield**: 32.5 mg (146 µmol, 65 %). $\mathbf{R_f}$ = 0.50 (*n*-hexane/ethyl acetate, 5:1, *v*/*v*). ^{1}**H NMR** (300 MHz, $CDCl_3$): δ = 3.33 – 3.21 (m, 4H), 2.24 – 2.12 (m, 4H), 1.96 – 1.77 (m, 4H), 1.44 (s, 9H) ppm. ^{13}**C NMR** (75 MHz, $CDCl_3$): δ = 156.1, 85.2, 84.6, 79.2, 53.5, 53.1, 29.7, 28.7, 18.9, 18.8 ppm. **IR** (film): $\tilde{\nu}$ = 3003 (w), 2971 (m), 2928 (m), 2850 (w), 1692 (s), 1462 (m), 1409 (m), 1362 (s), 1344 (m), 1300 (w), 1272 (m), 1254 (m), 1209 (w), 1171 (s), 1120 (m), 1038 (w), 1009 (m), 986 (w), 888 (w), 776 (w), 545 (w) cm^{-1}. **UV/Vis** (DCM): λ_{max} = 272, 228 nm. **HR-ESI-MS** (MeOH) m/z: $C_{13}H_{21}NO_2$ calc.: 246.1464 $[M+Na]^+$, meas.: 246.1465.

Tert-butyl 1-benzyl-4,5,6,8,9,10-hexahydro-[1,2,3]triazolo[4,5-e]azonine-7(1*H*)-carboxylate (**S1a**)

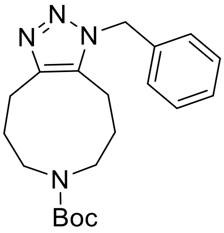

To a sample from the NMR-based kinetic experiment between Boc-ACN (**1a**) and benzyl azide were added 10.2 µL (10.5 mg, 79.0 µmol, 5.0 equiv.) benzyl azide. The reaction was stirred for 17 days at 40 °C, subsequently adsorbed onto silica and purified by column chromatography (DCM/methanol, 1:0 to 100:1 to 50:1 to 20:1 *v*/*v*) to give the desired product as a colorless oil. **Yield**: 4.0 mg (11.2 µmol, 71 %). $\mathbf{R_f}$ = 0.26 (DCM/methanol, 20:1, *v*/*v*). ^{1}**H NMR** (300 MHz, CD_3CN): δ = 7.40 – 7.26 (m, 3H), 7.22 – 7.14 (m, 2H), 5.46 (s, 1H), 5.41 (s, 1H), 3.18 – 2.92 (m, 4H), 2.85 – 2.63 (m, 4H), 2.14 (s, 6H), 2.02 – 1.96 (m, 2H), 1.85 (s, 2H) ppm. ^{13}**C NMR** (76 MHz, CD_3CN): δ = 156.4, 145.7, 137.3, 133.9, 129.8, 128.9, 128.2, 79.5, 52.3, 52.2, 52.0, 30.9, 30.3, 28.5, 23.6, 21.2 ppm. **IR** (film): $\tilde{\nu}$ = 2973 (m), 2926 (m), 2856 (m), 1691 (s), 1481 (m), 1455 (m), 1414 (m), 1364 (s), 1315 (m), 1295 (w), 1251 (m), 1231 (m), 1173 (m), 1159 (m), 1120 (m), 964 (w), 883 (w), 755 (m), 697 (w) cm^{-1}. **UV/Vis** (DCM): λ_{max} = 230 nm. **HR-ESI-MS** (MeOH) m/z: $C_{20}H_{28}N_4O_2$ calc.: 357.2285 $[M+H]^+$, meas.: 357.2273.

NMR spectra

^{1}H and ^{13}C NMR of compound **1**

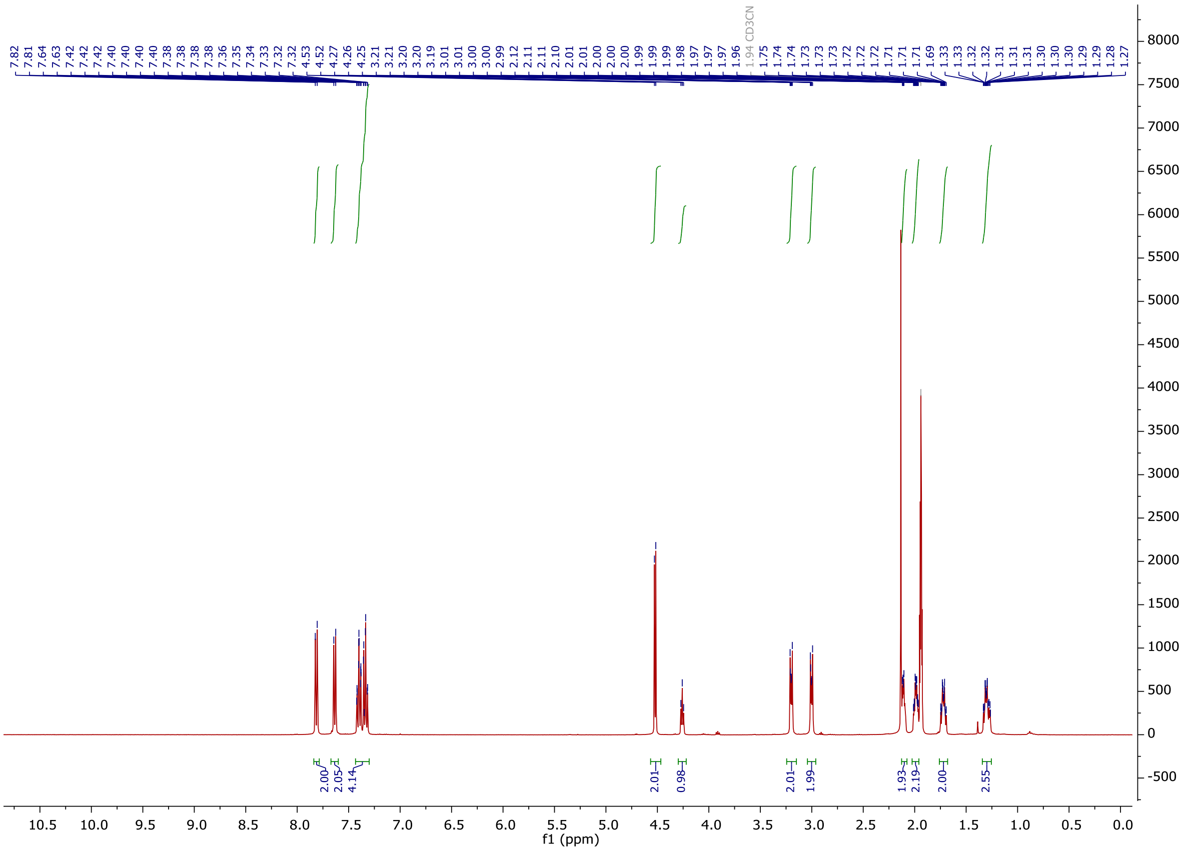

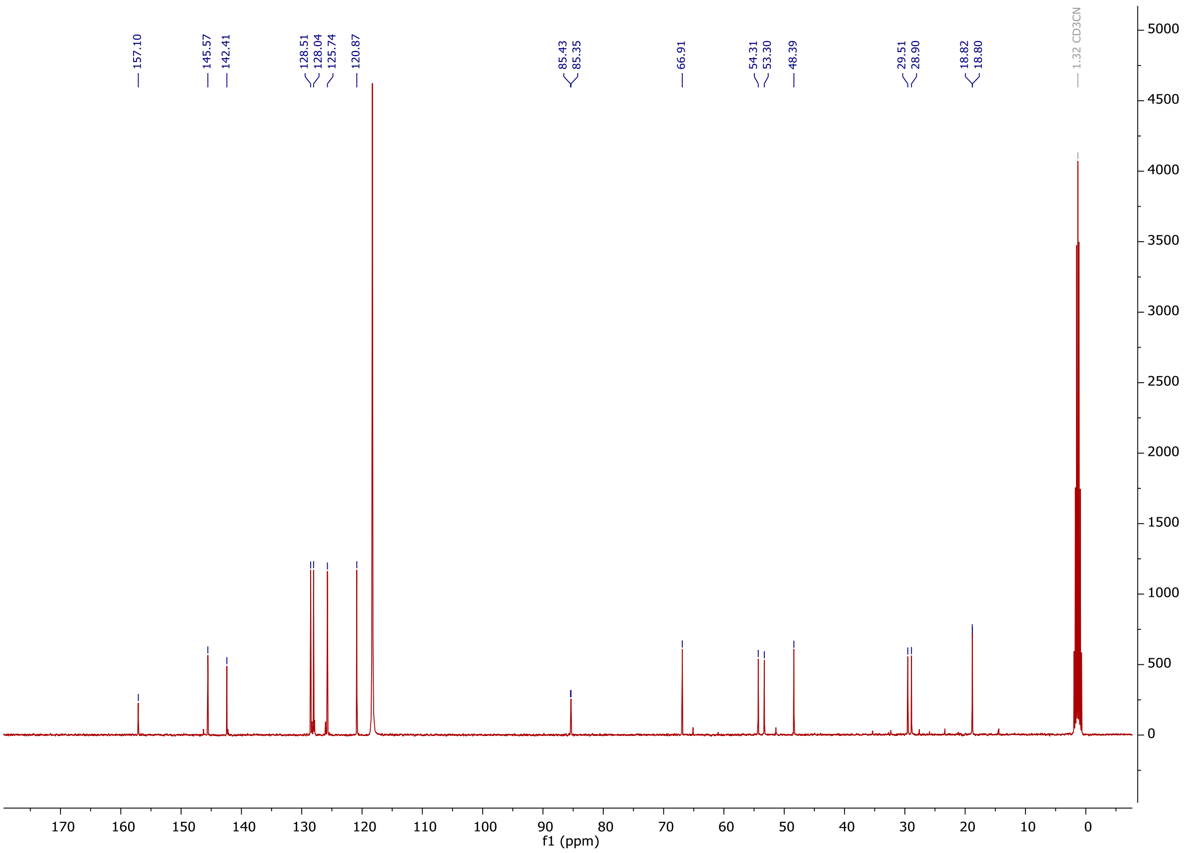

^{1}H and ^{13}C NMR of compound **3**

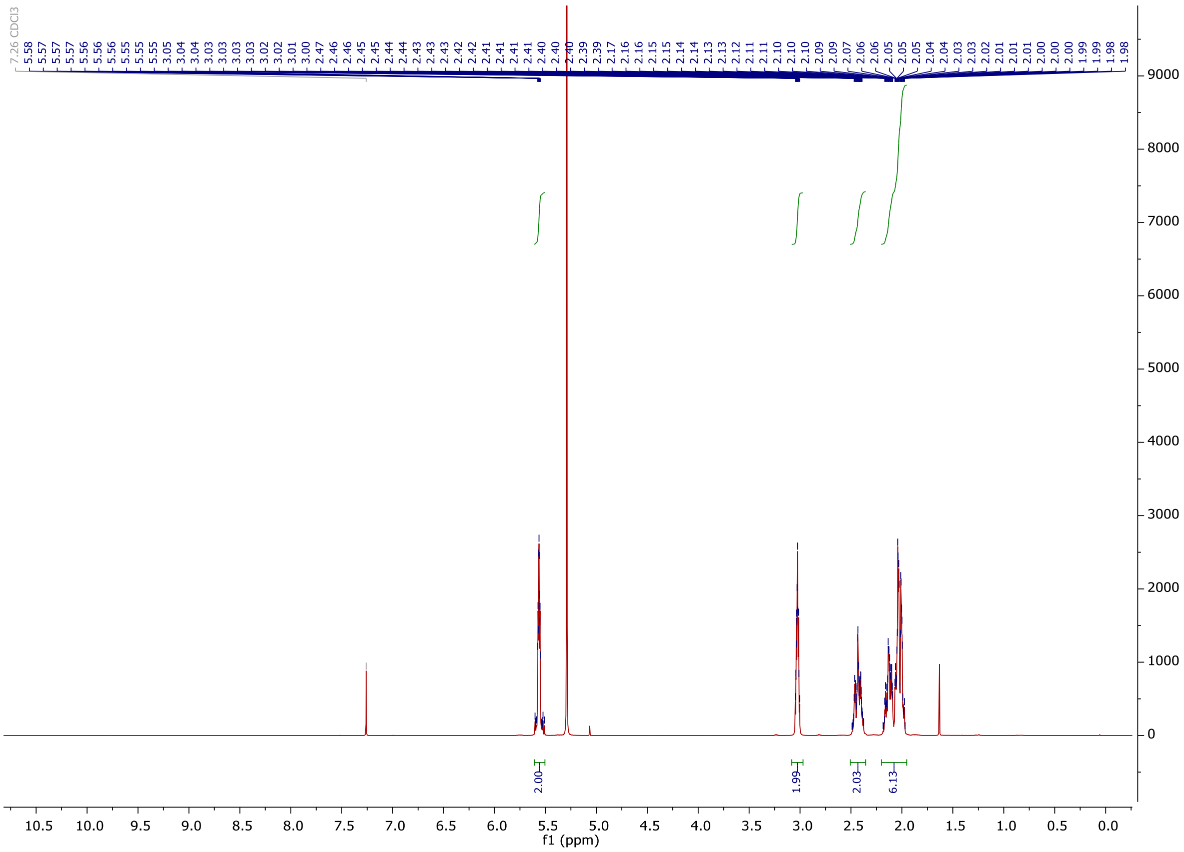

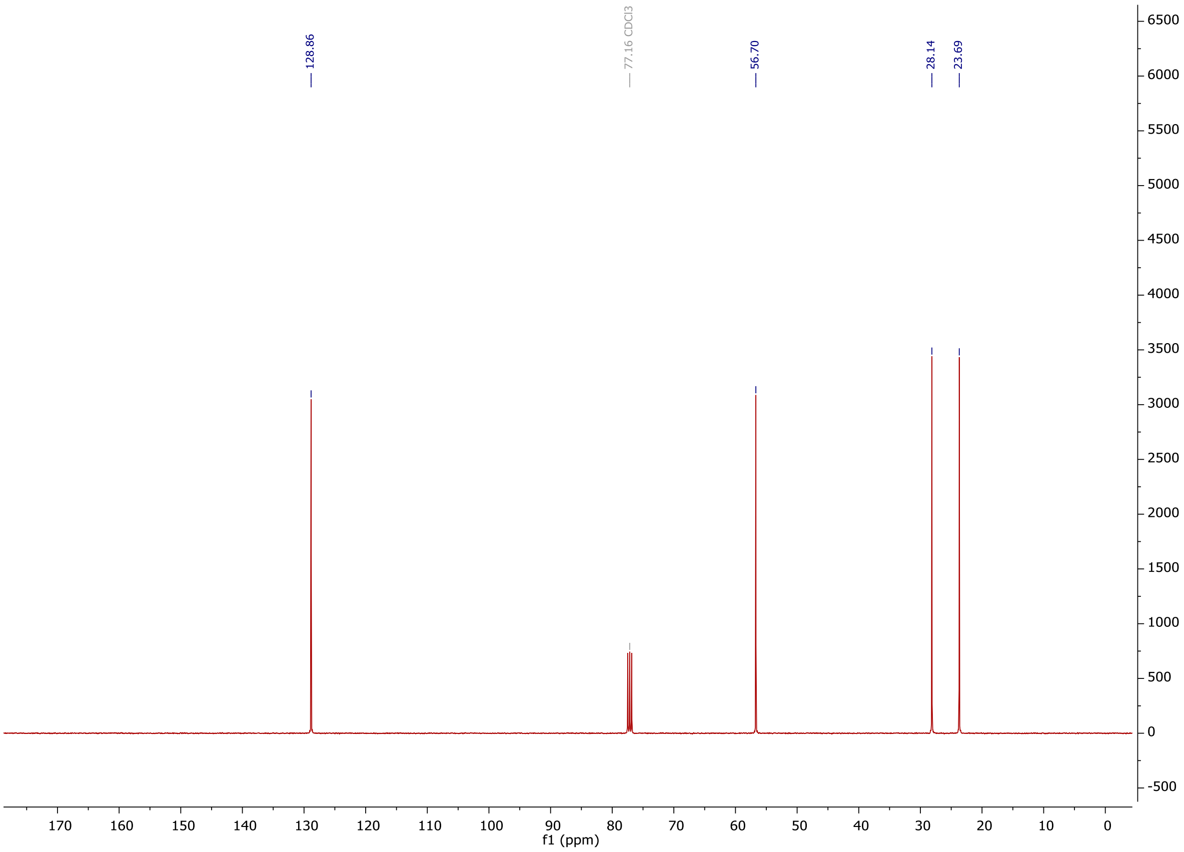

^{1}H and ^{13}C NMR of compound **4**

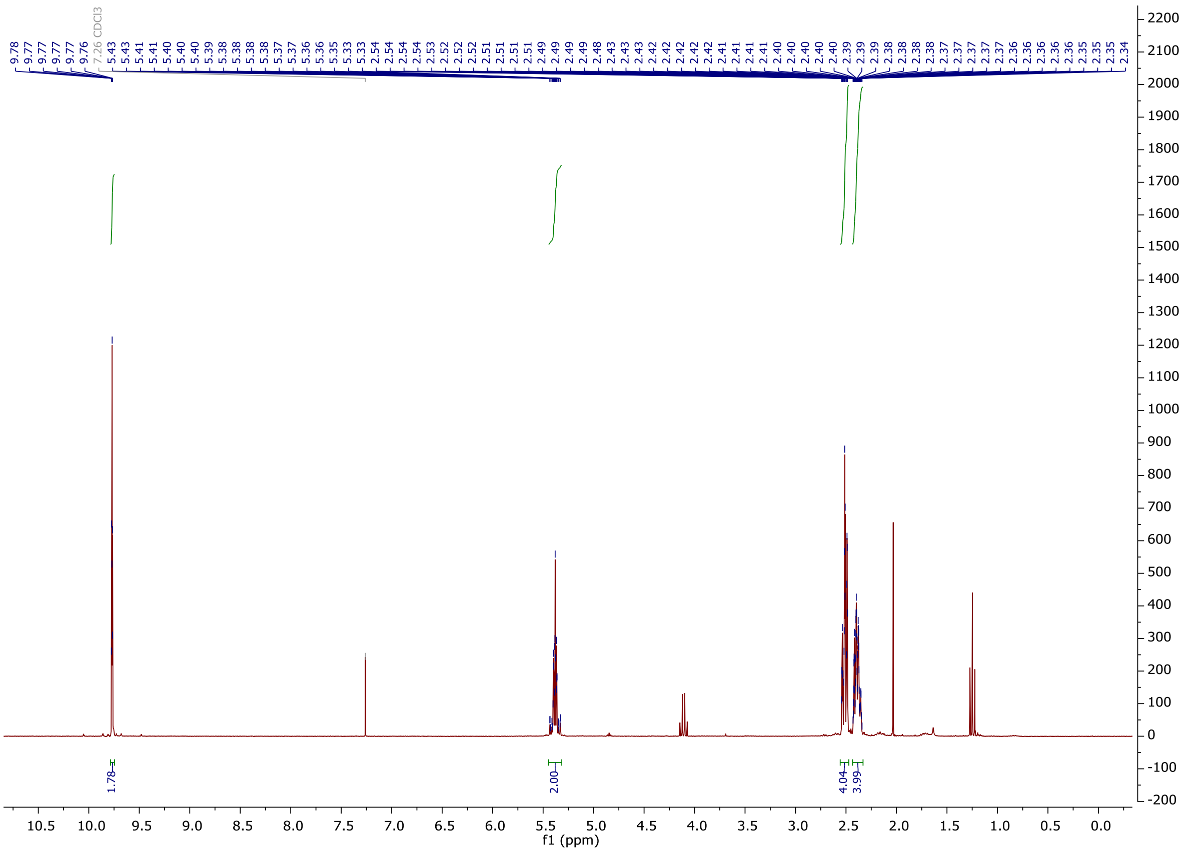

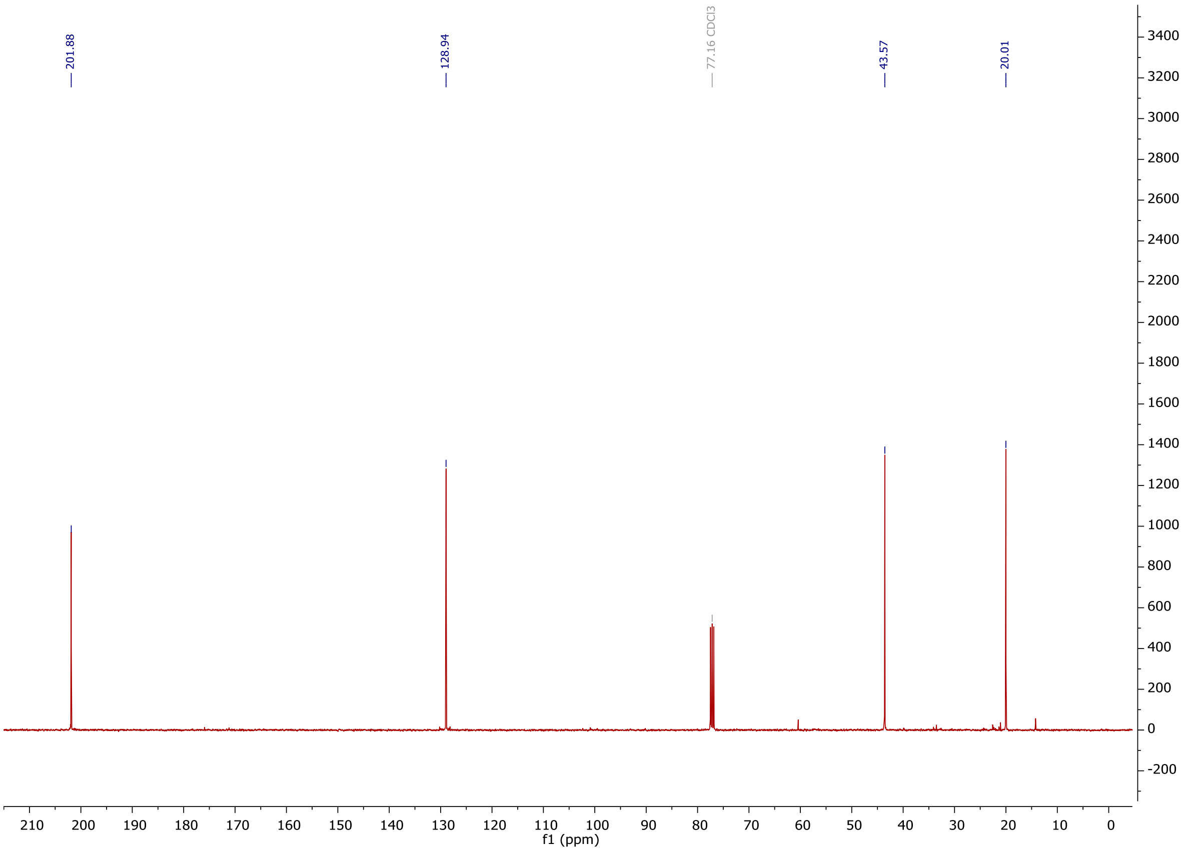

^{1}H and ^{13}C NMR of compound **5**

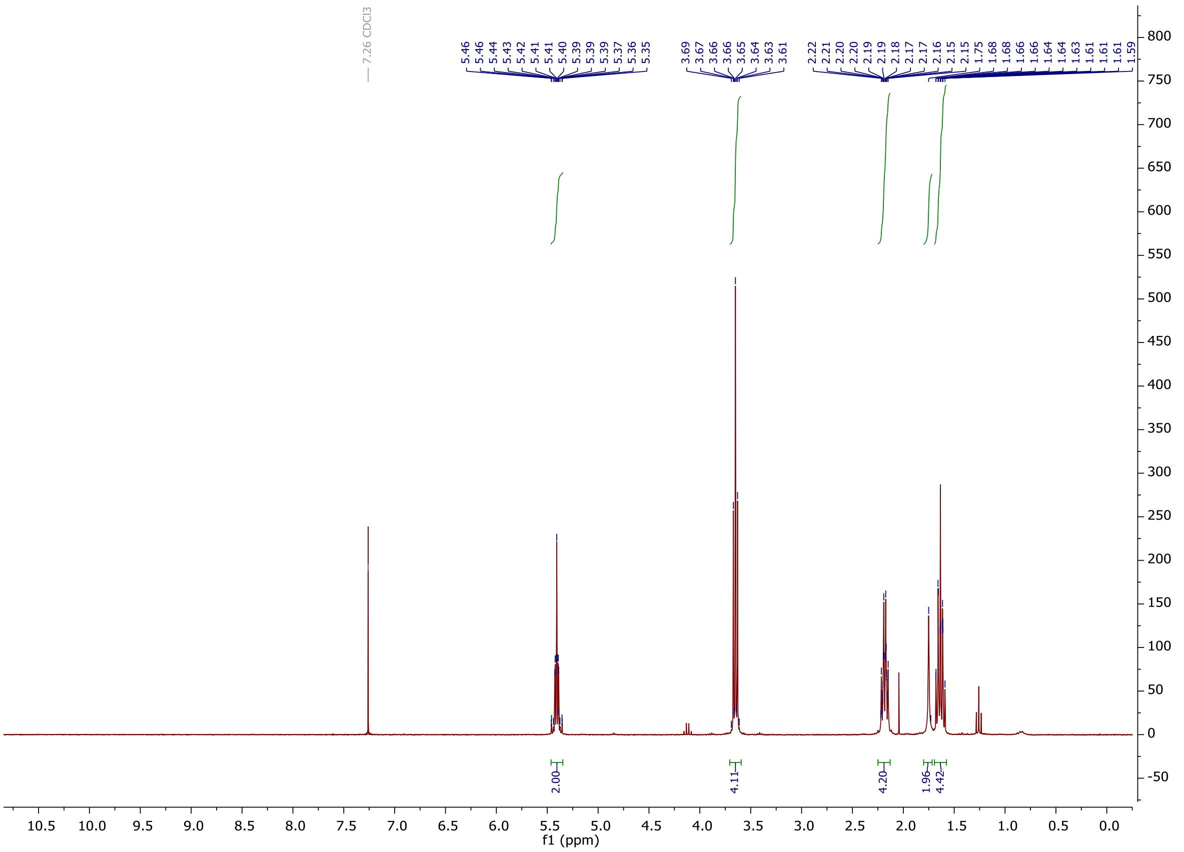

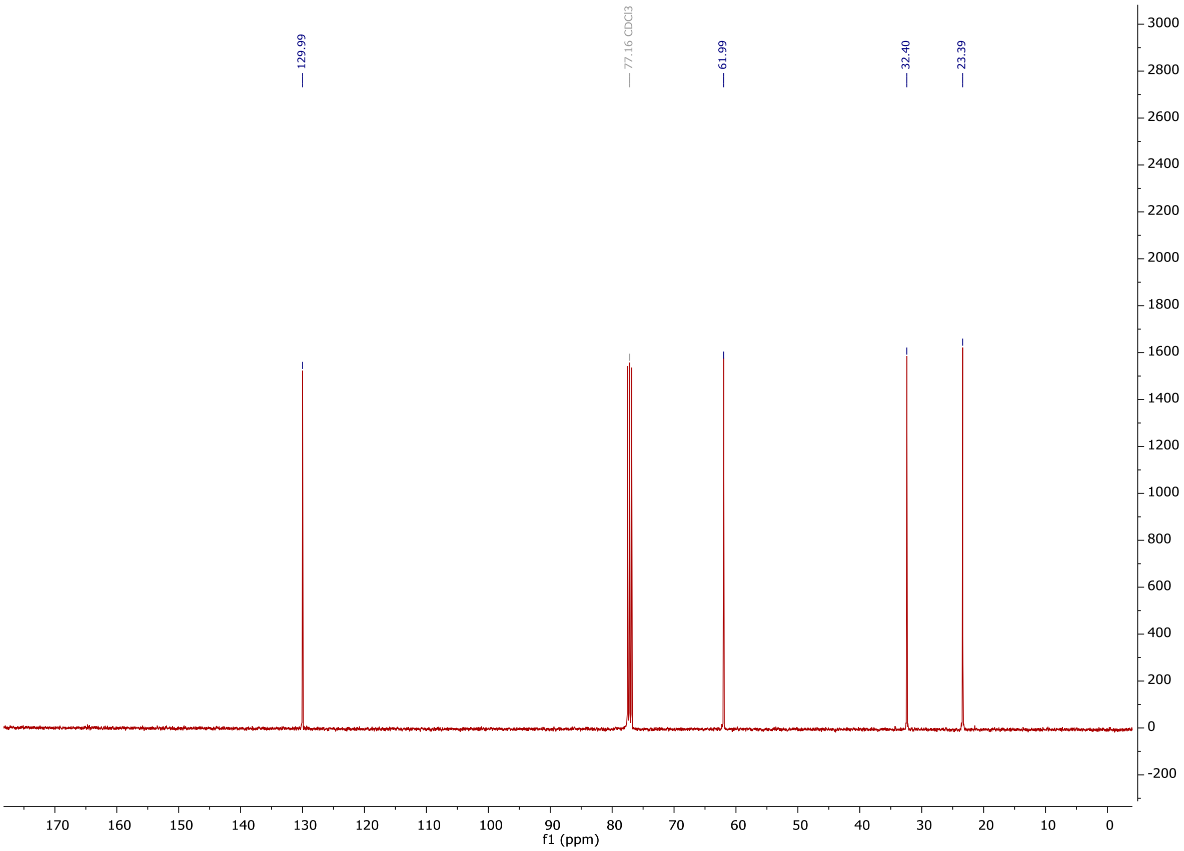

^{1}H and ^{13}C NMR of compound **6**

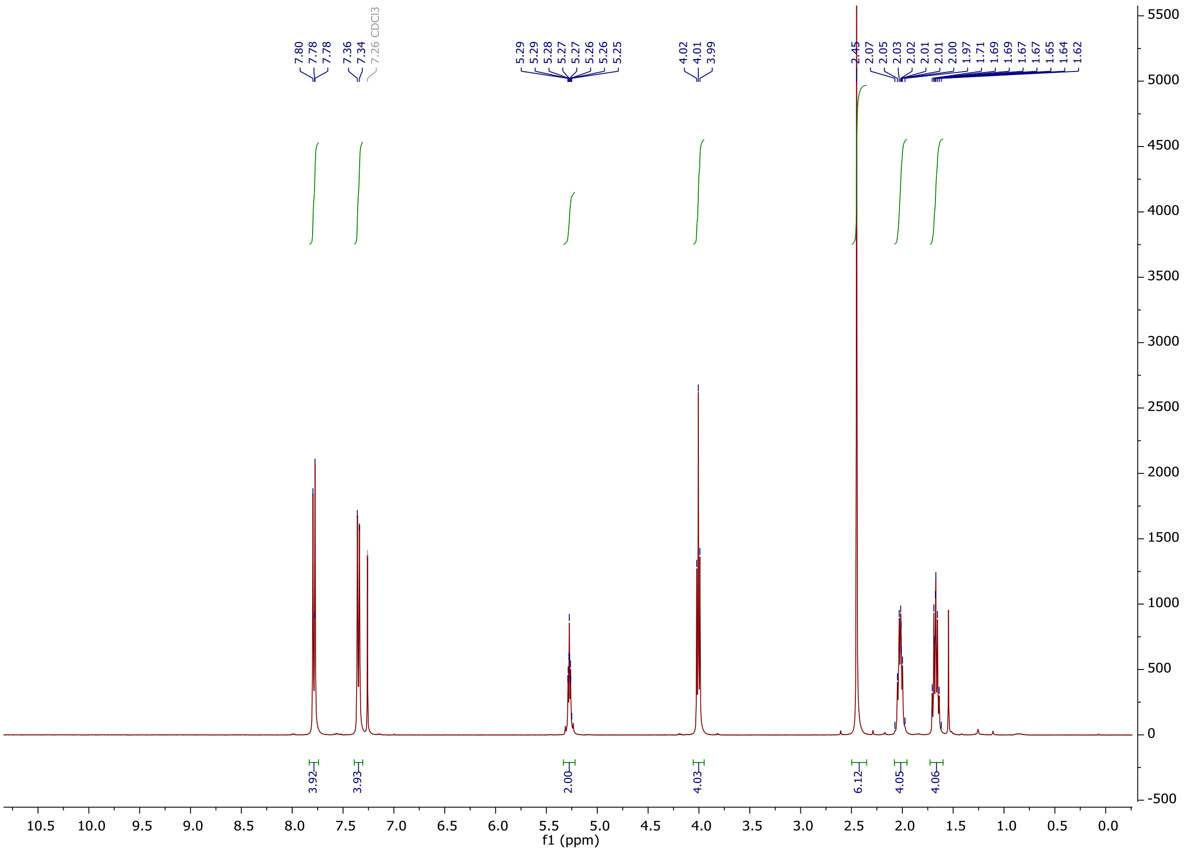

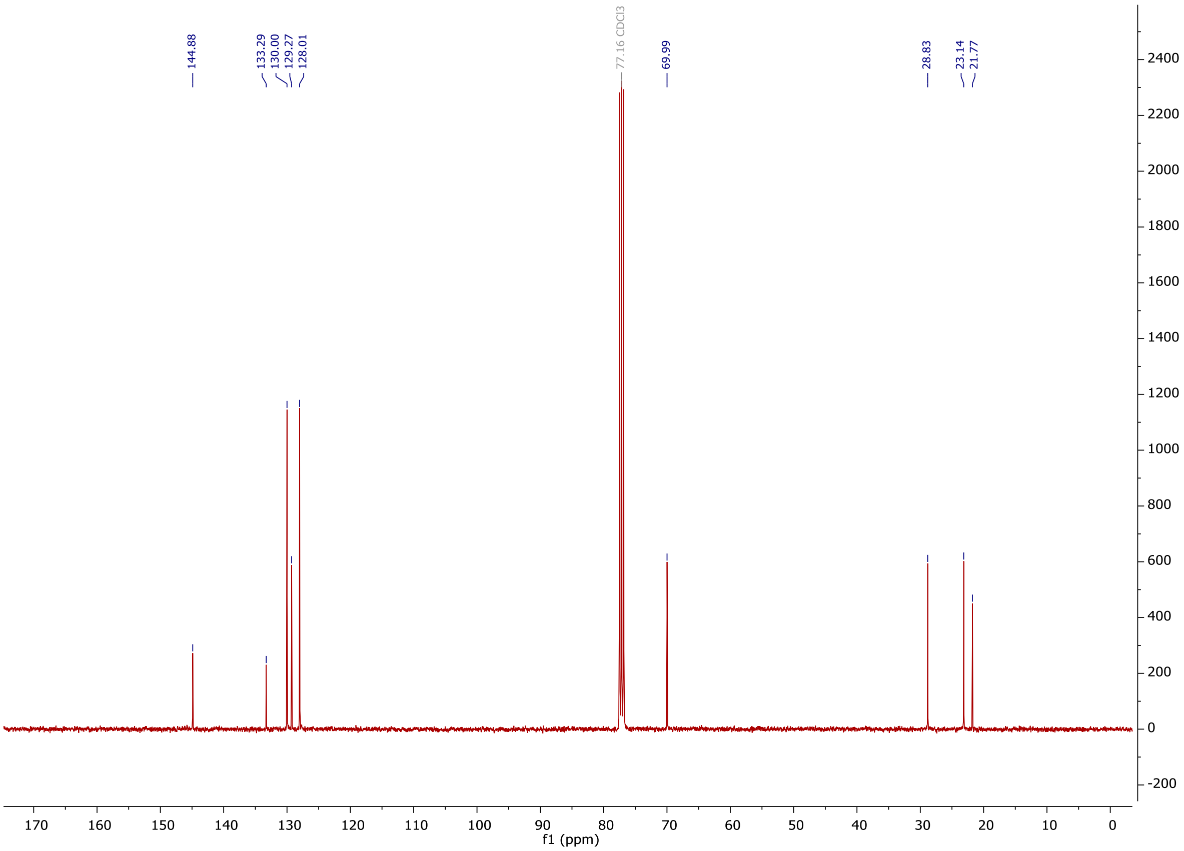

^{1}H and ^{13}C NMR of compound **7**

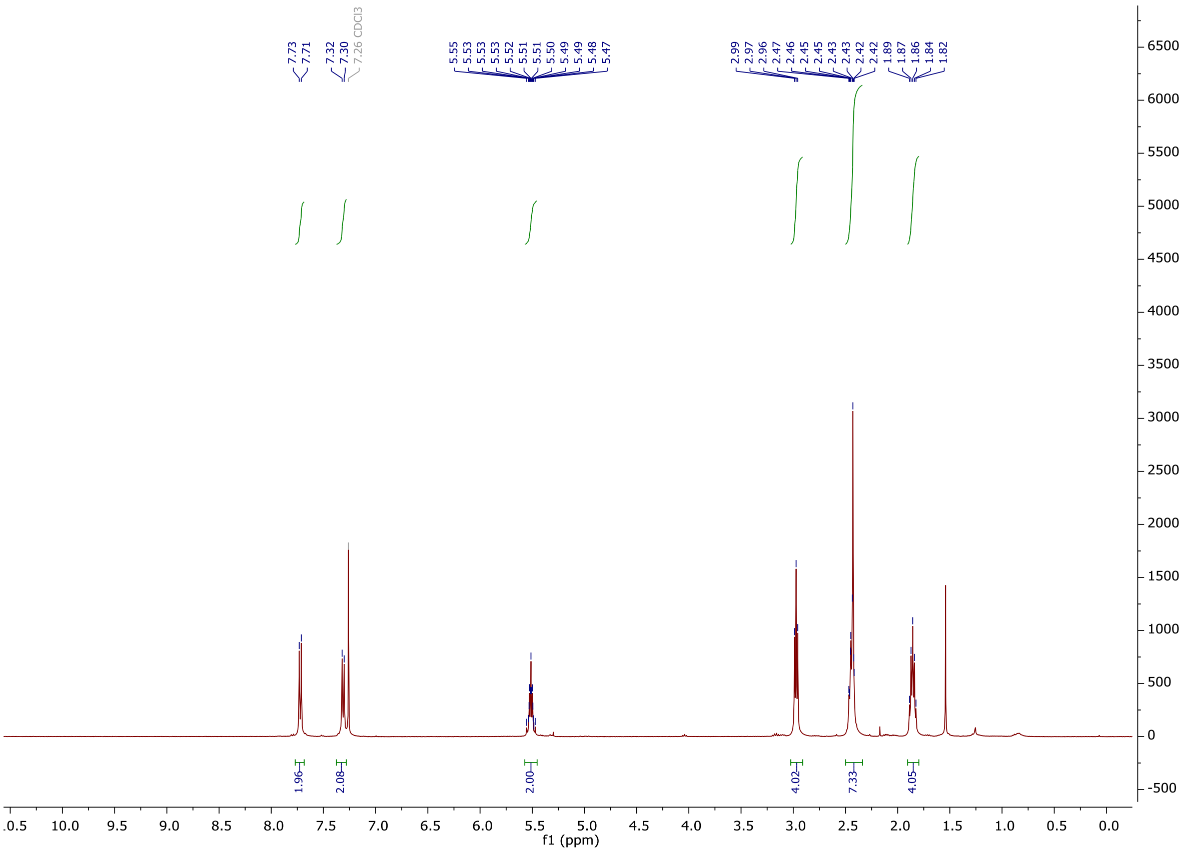

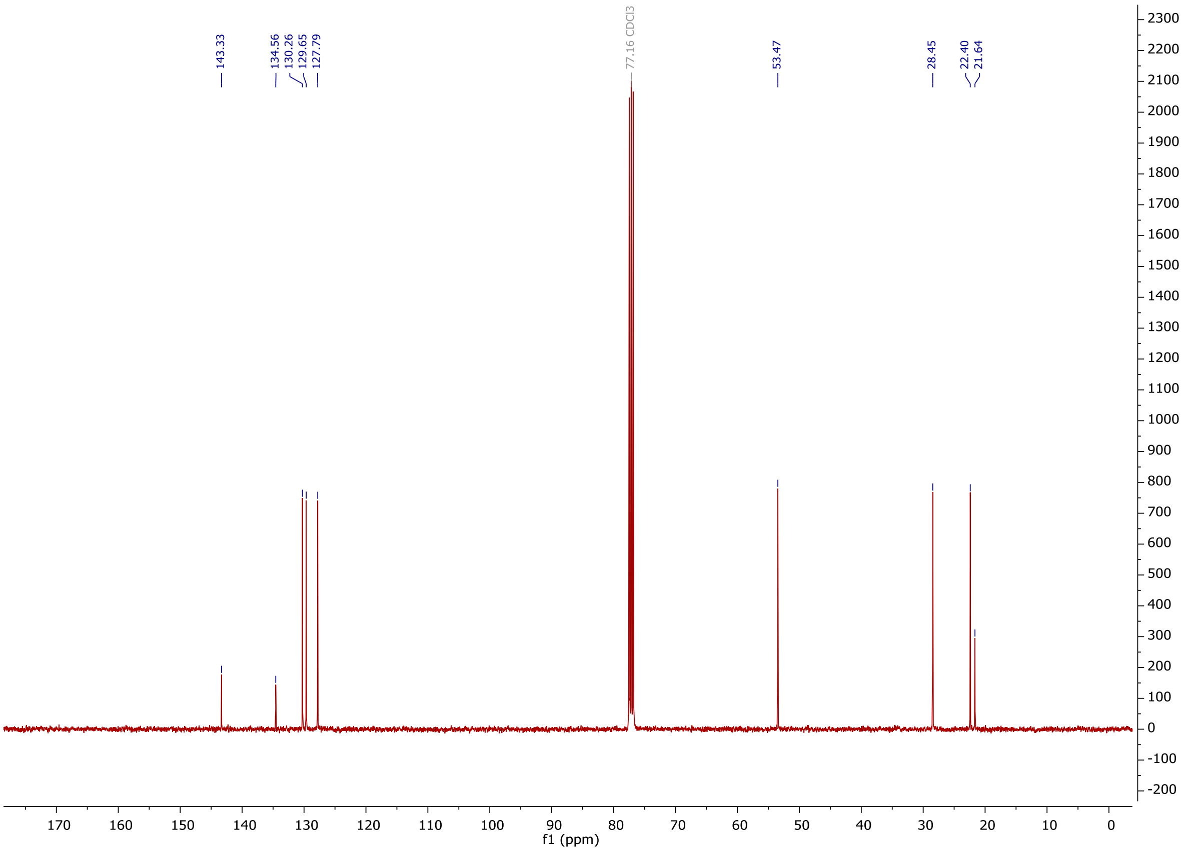

^{1}H and ^{13}C NMR of compound **8**

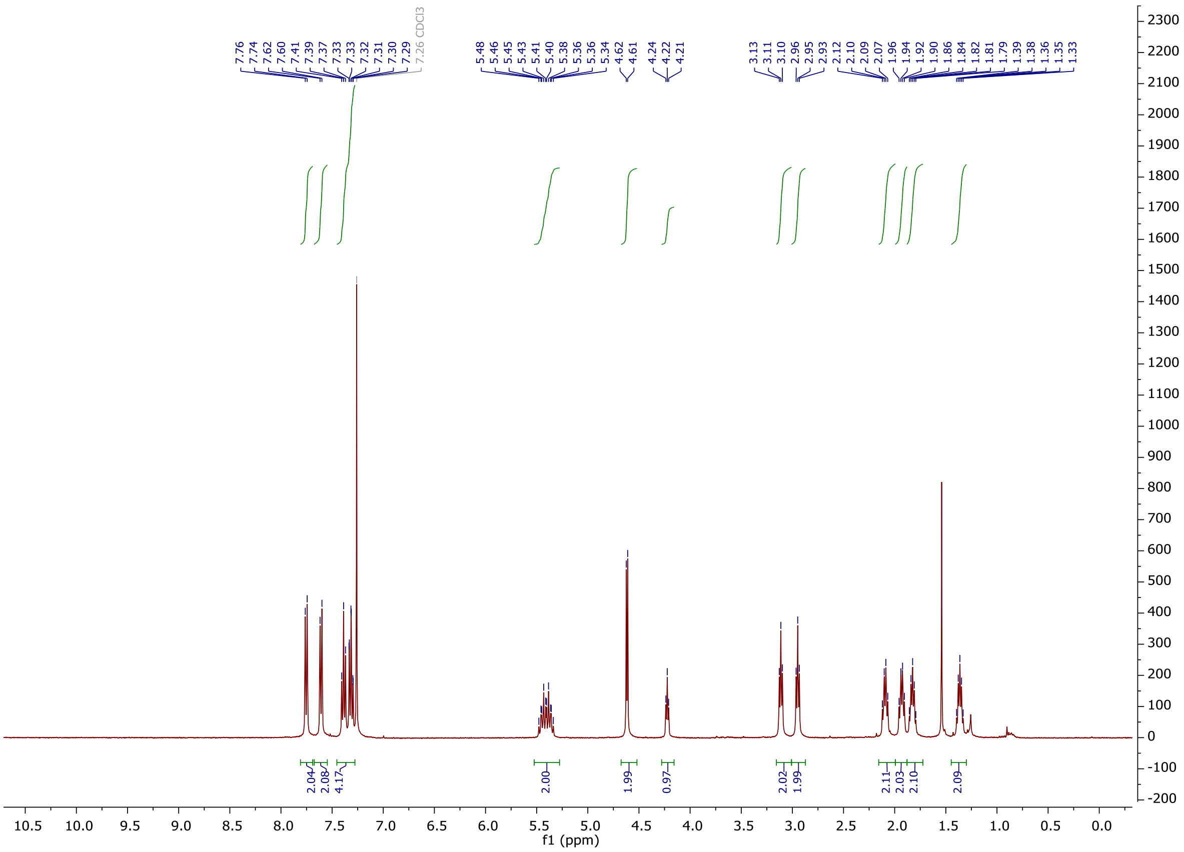

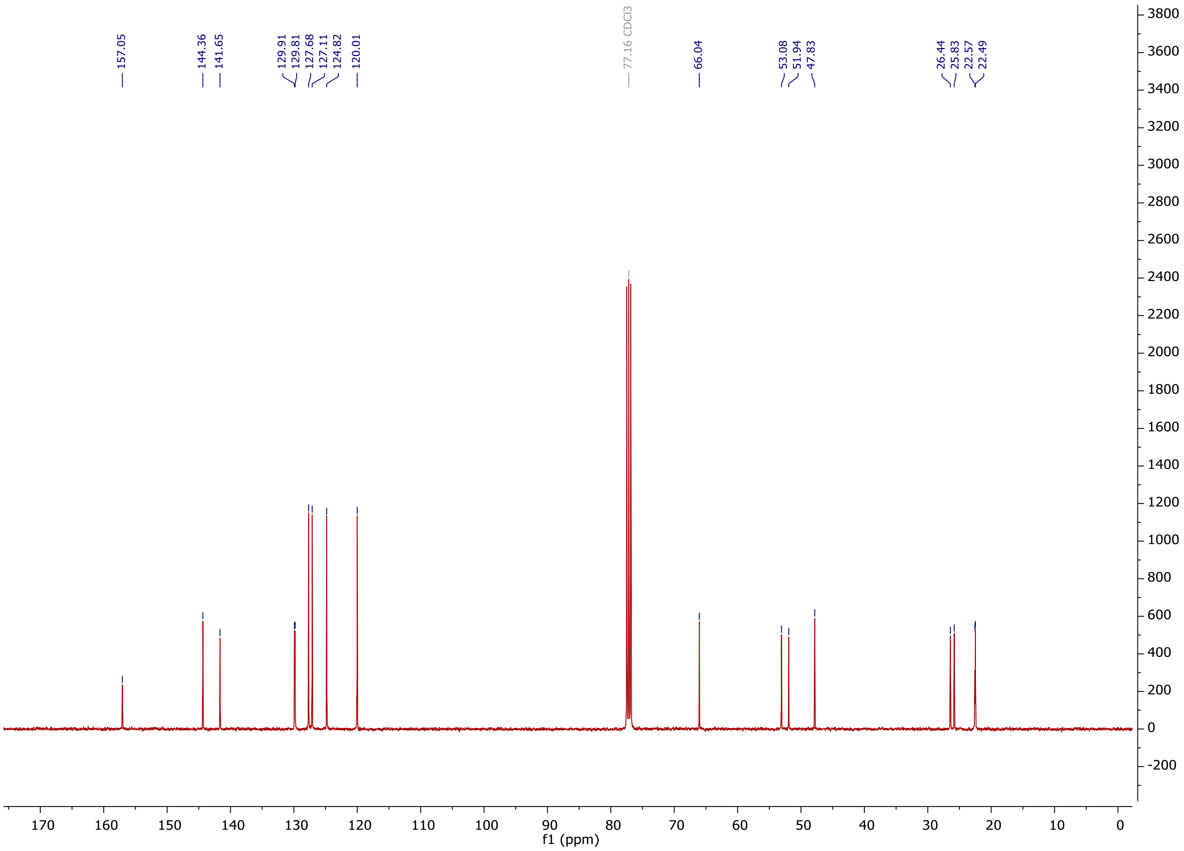

^{1}H and ^{13}C NMR of compound **9**

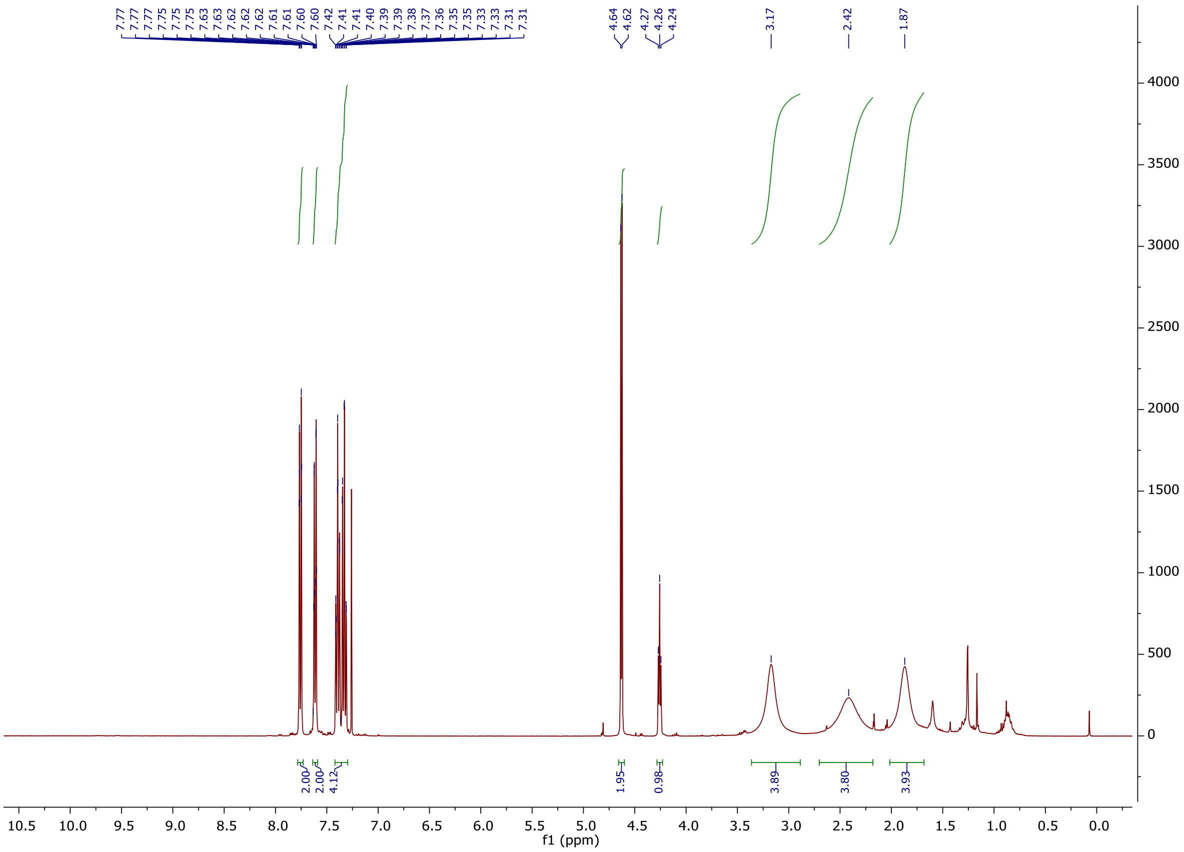

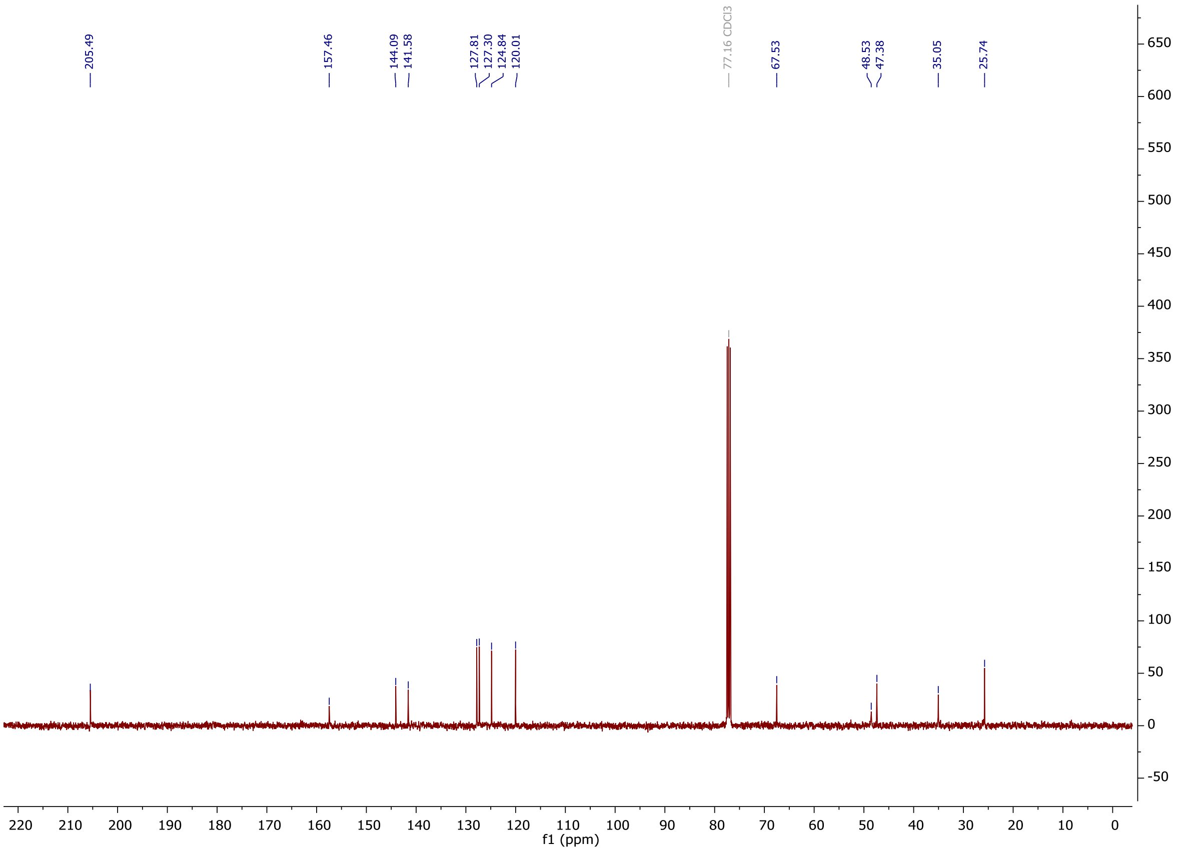

^{1}H and ^{13}C NMR of compound **10**

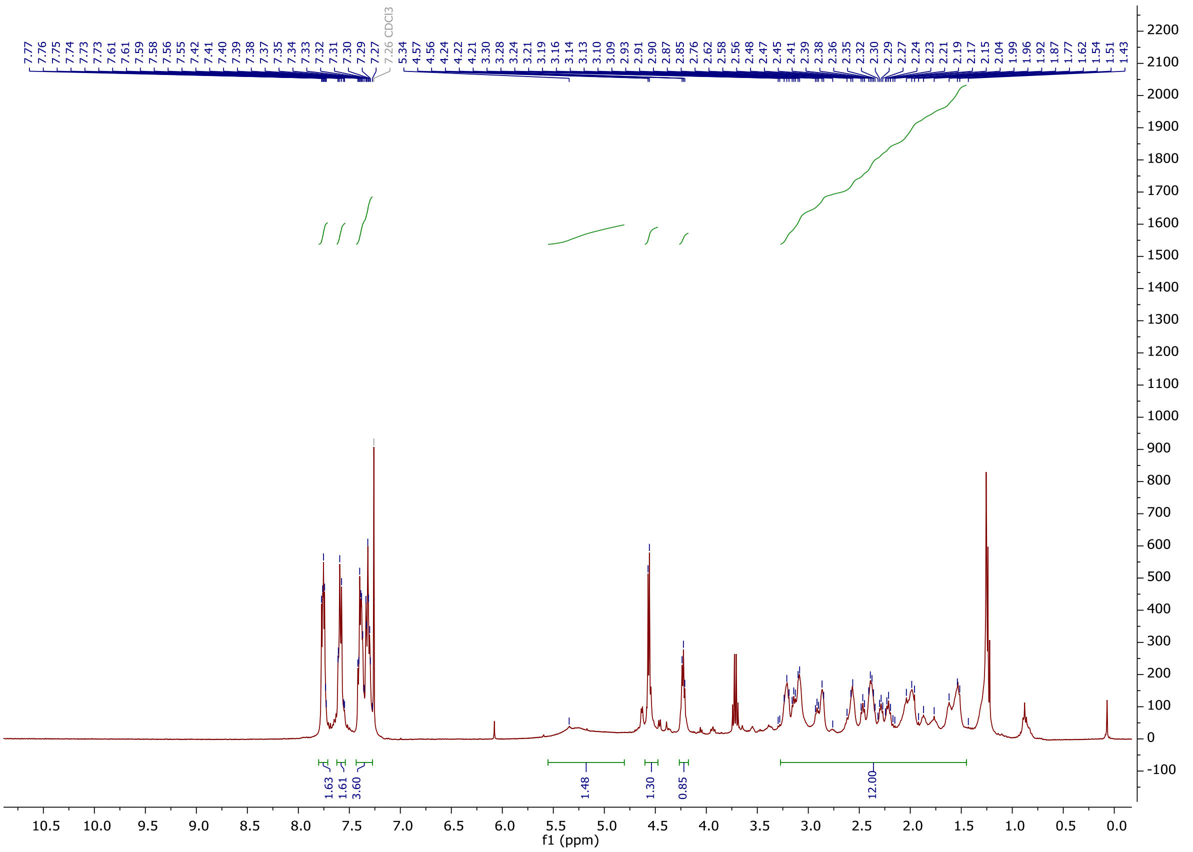

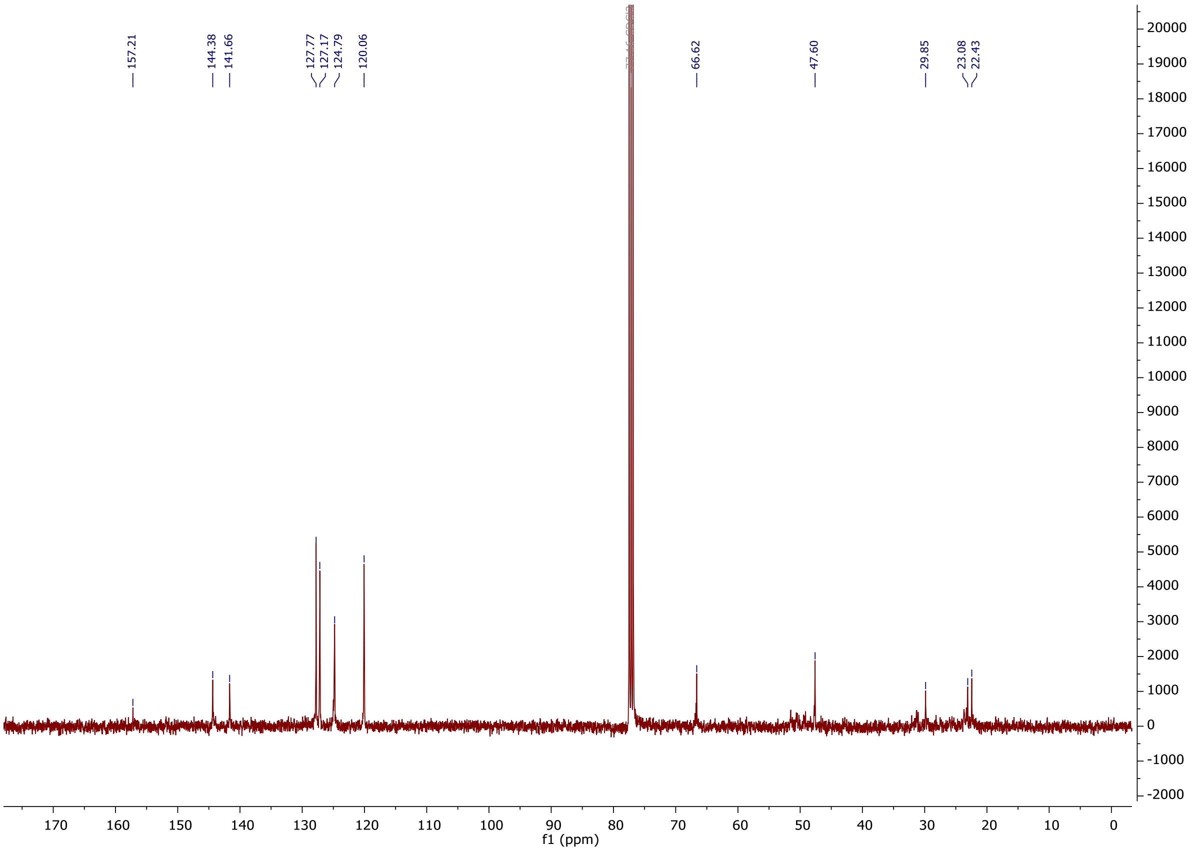

^{1}H and ^{13}C NMR of compound **12a**

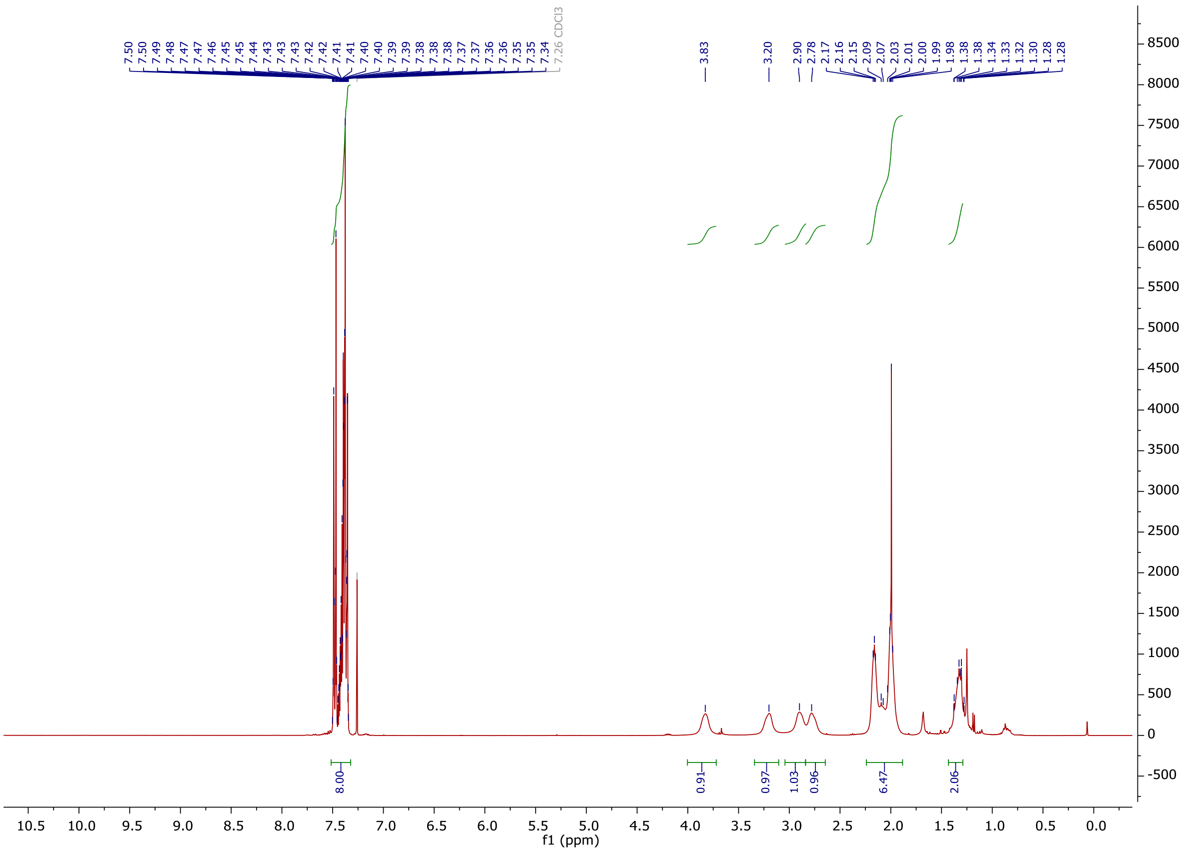

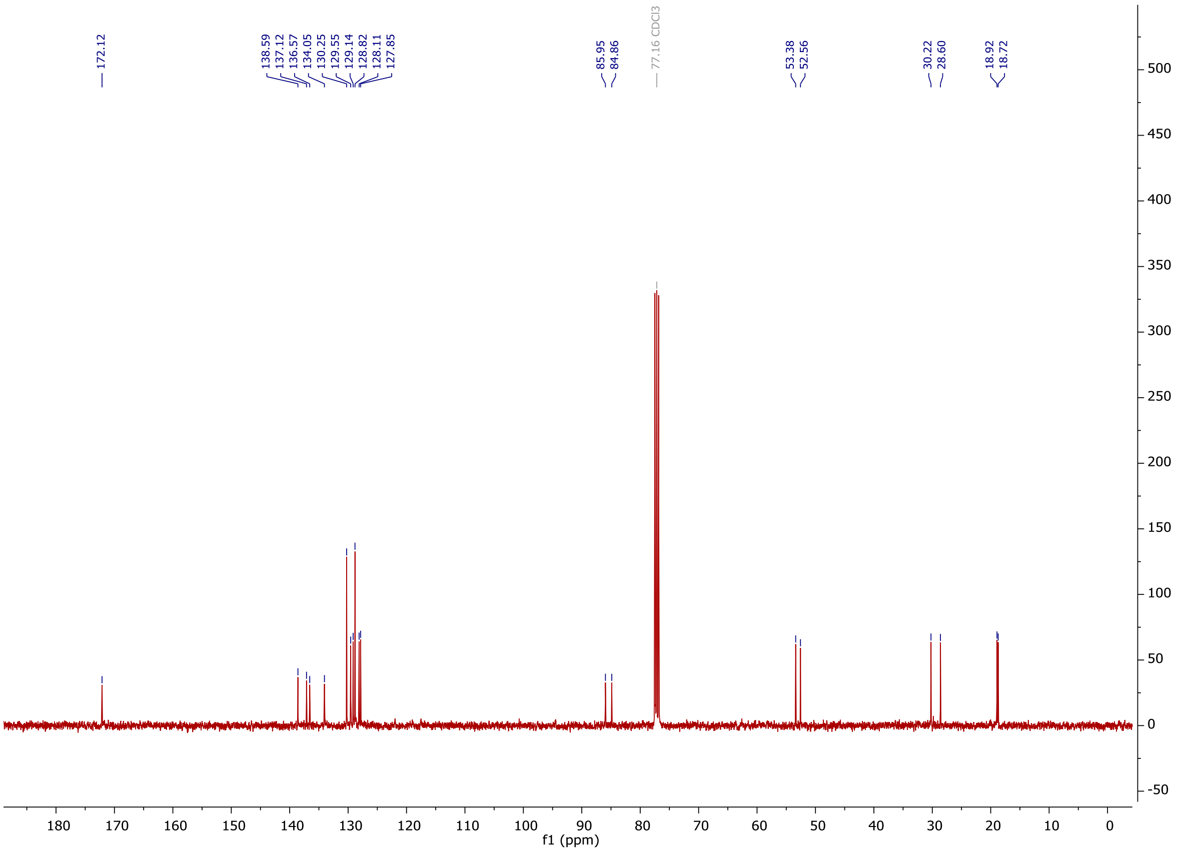

^{1}H and ^{13}C NMR of compound **11a**

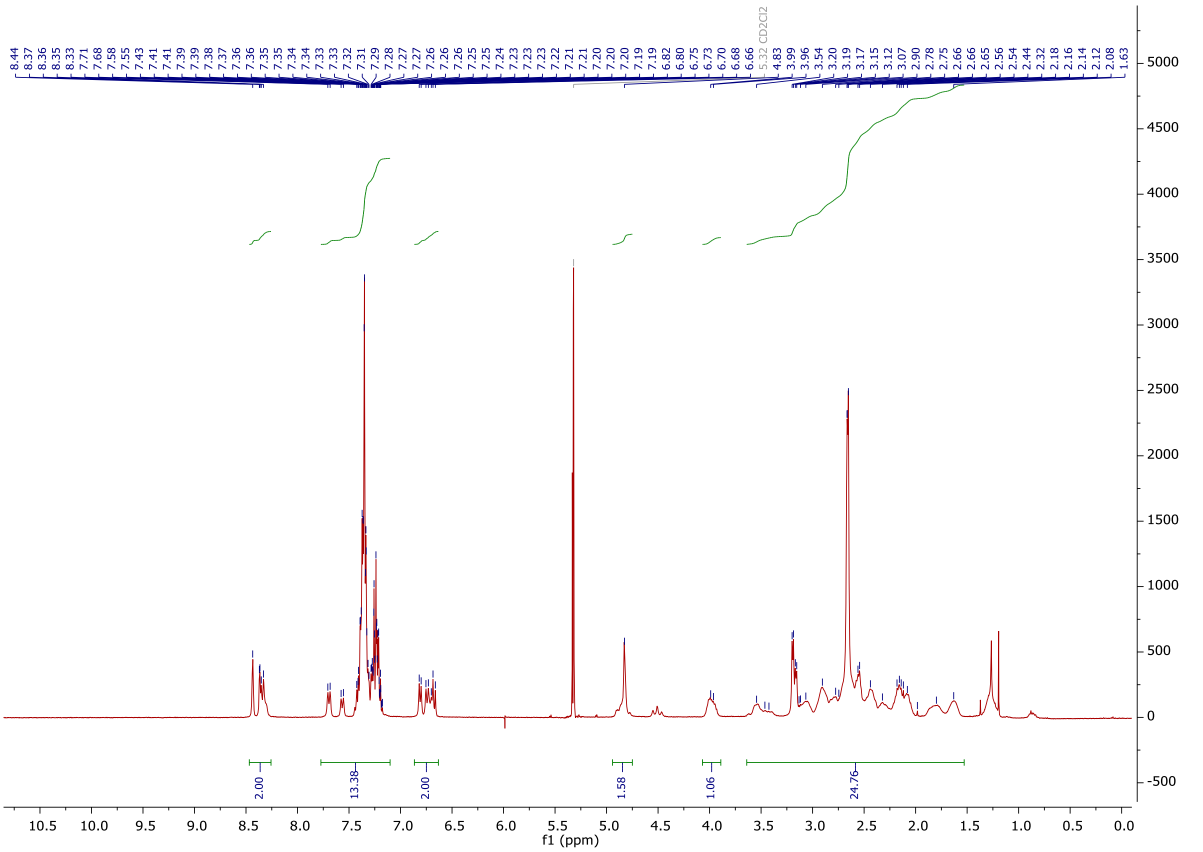

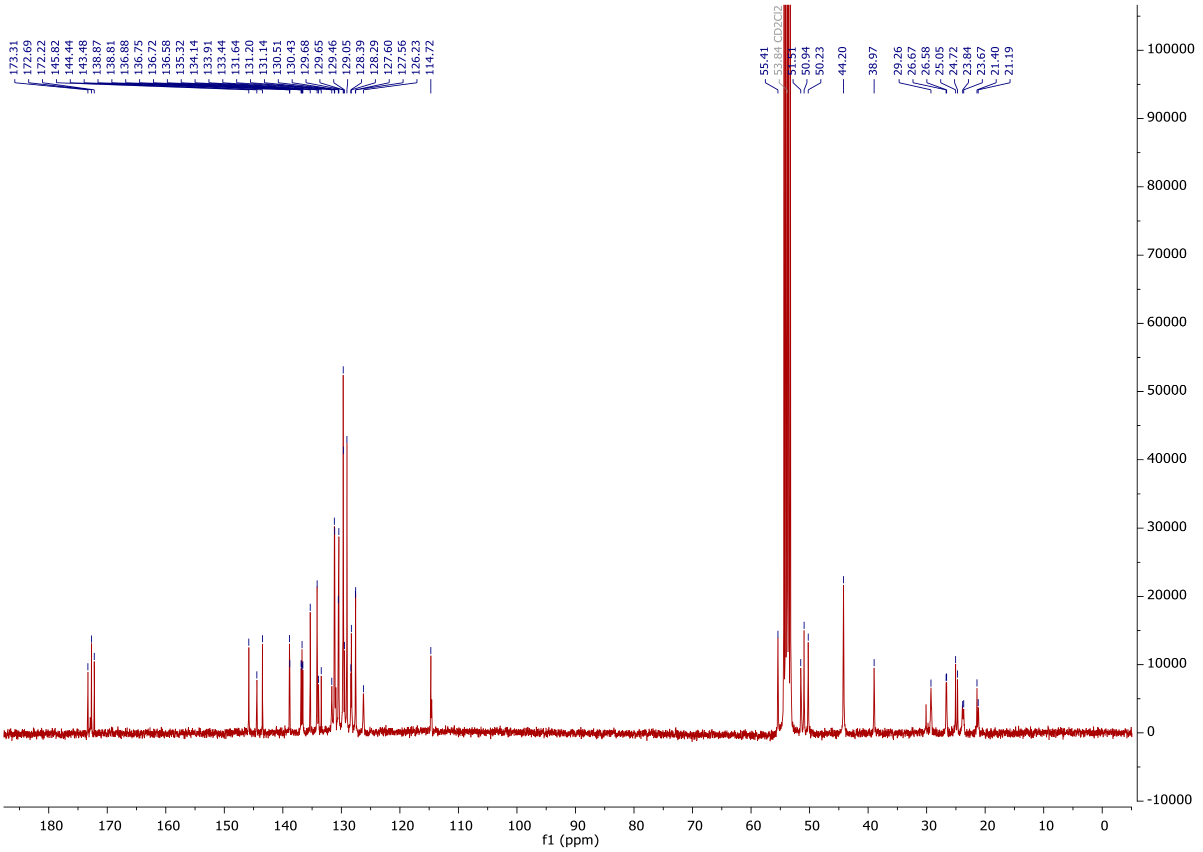

^{1}H and ^{13}C NMR of compound **12c**

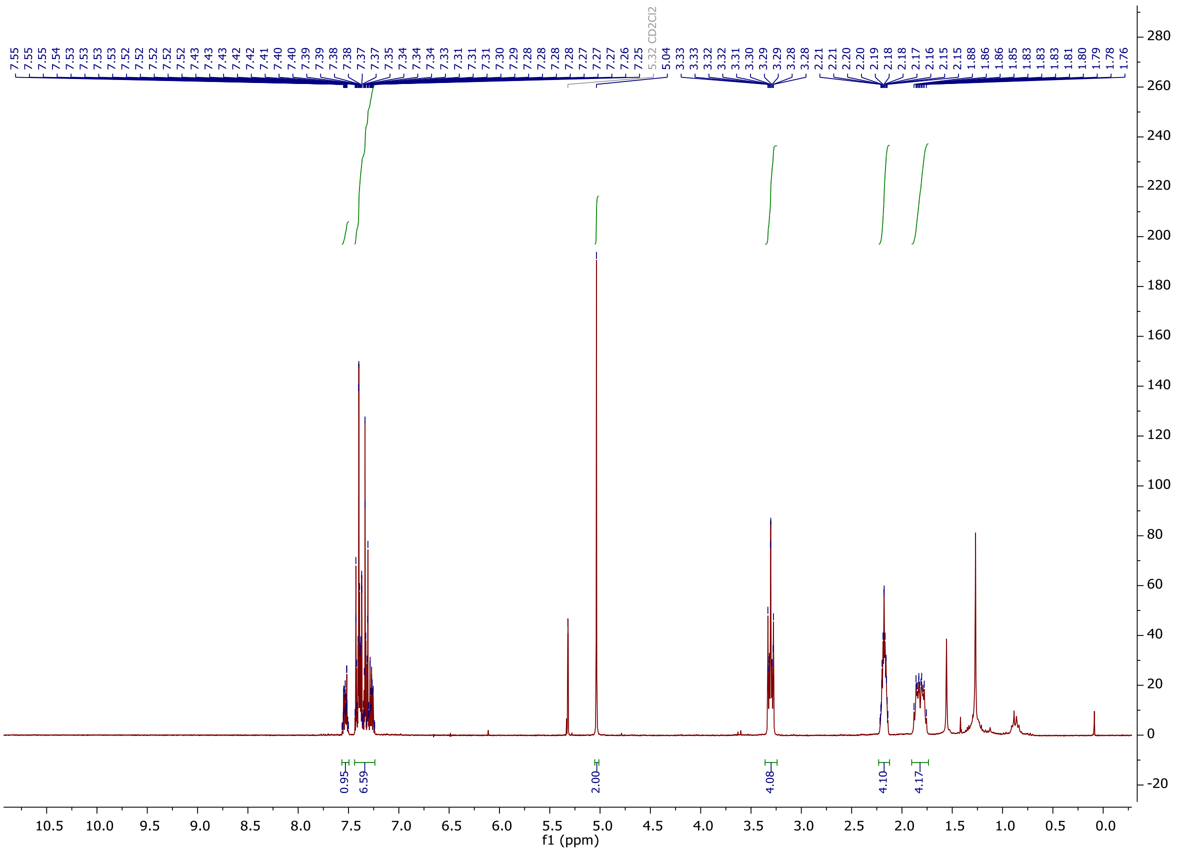

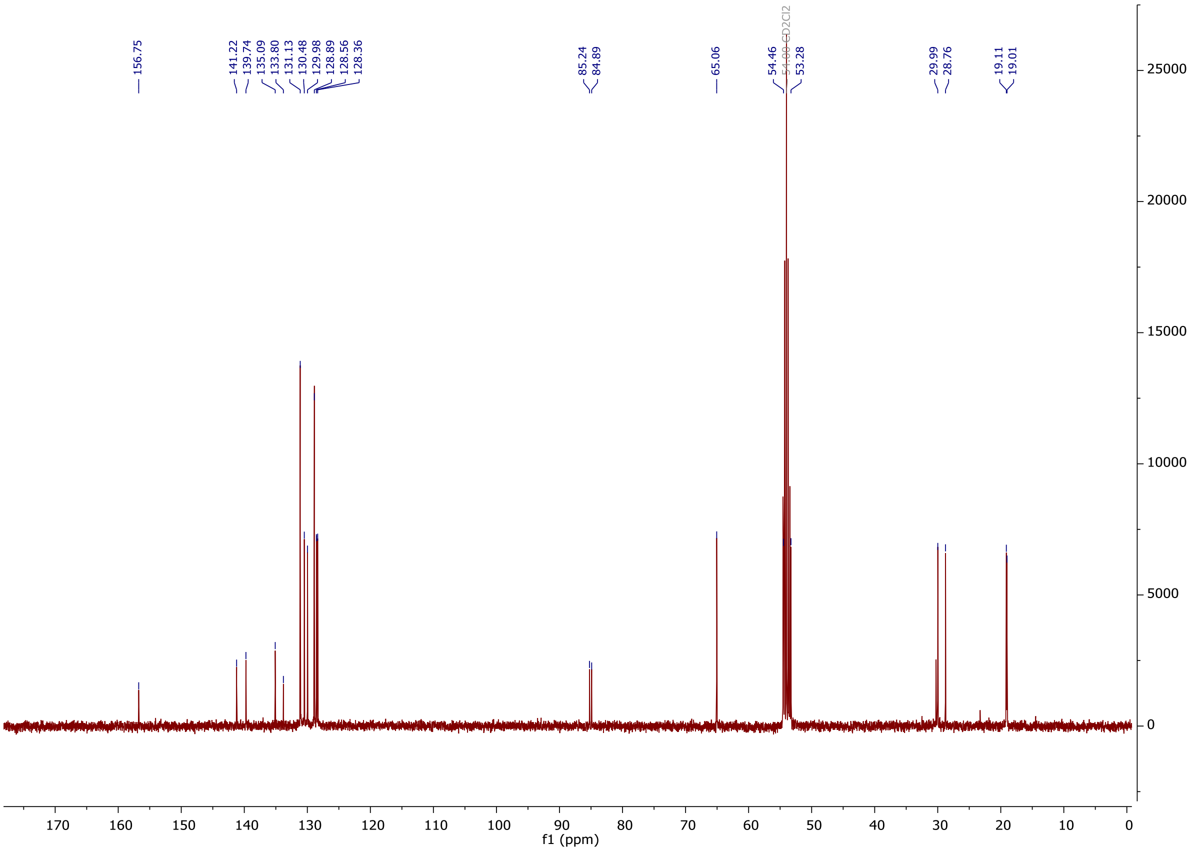

^{1}H and ^{13}C NMR of compound **11c**

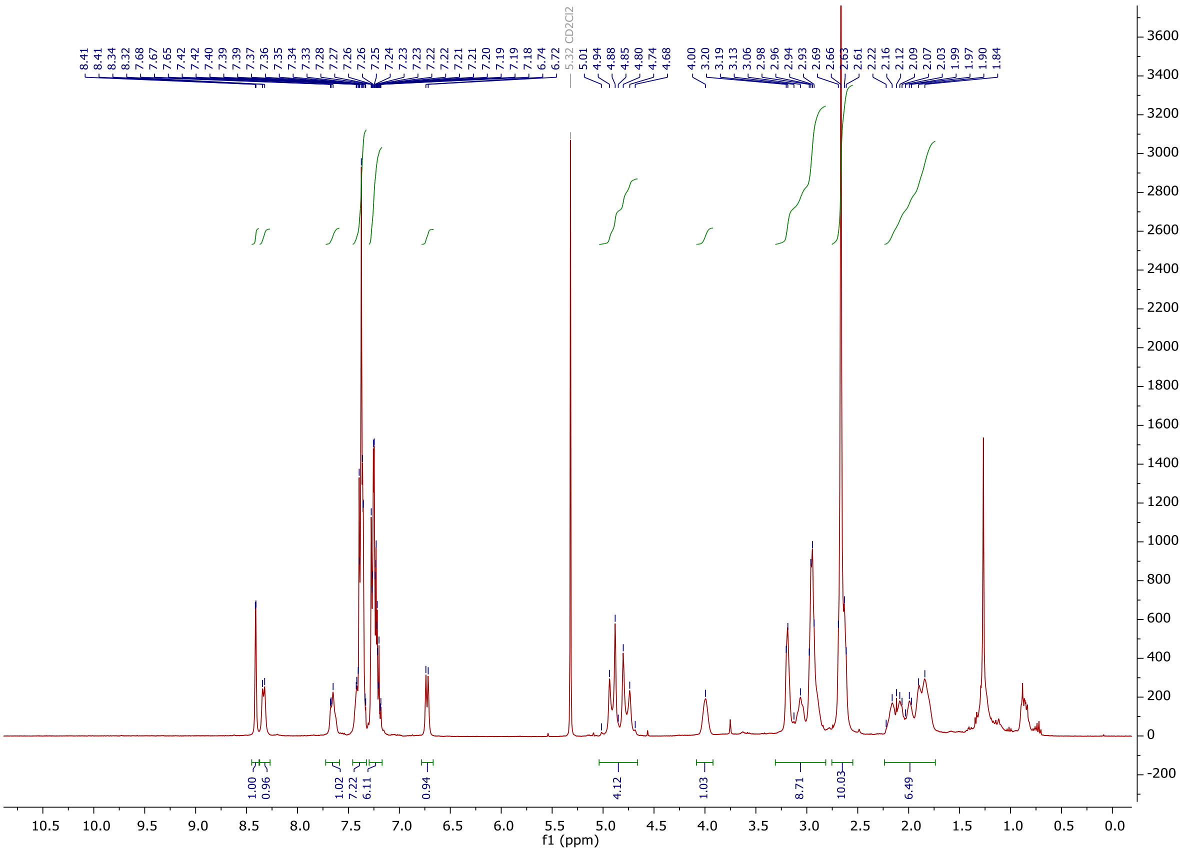

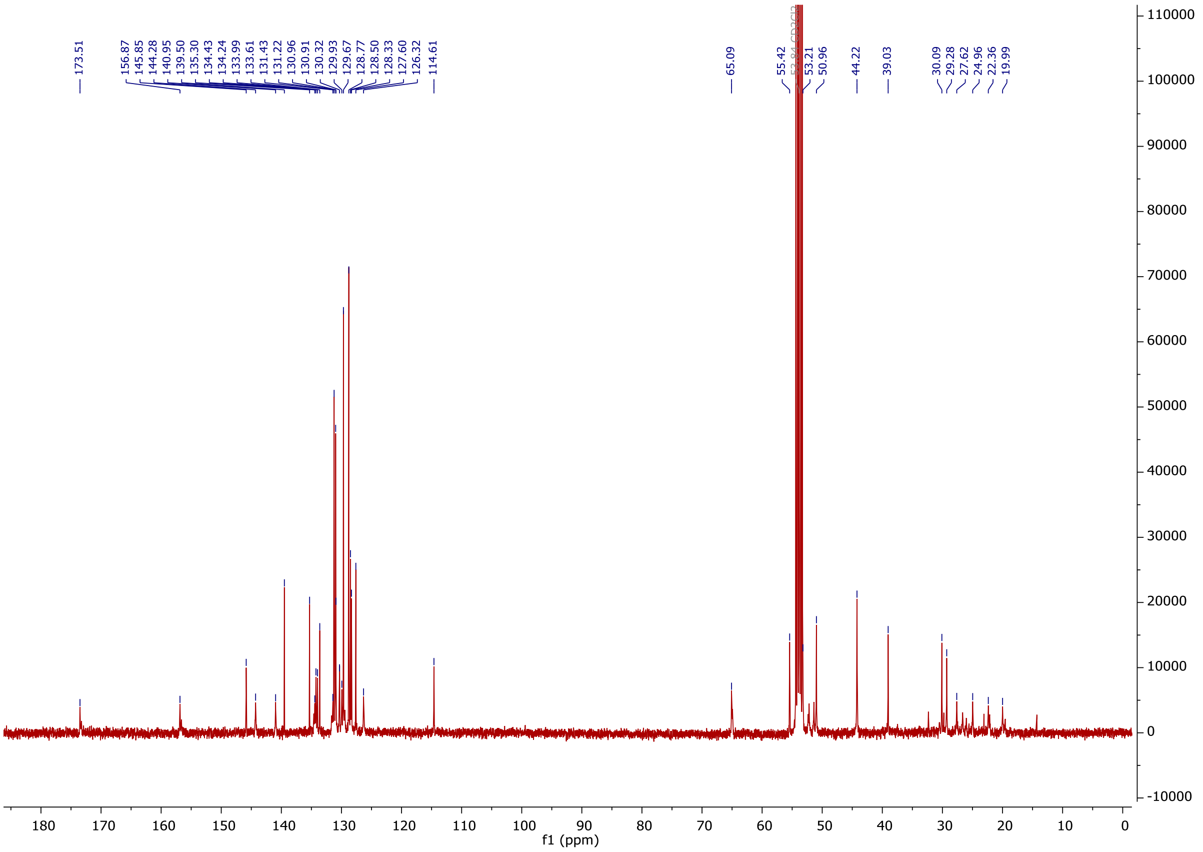

^{1}H and ^{13}C NMR of compound **16**

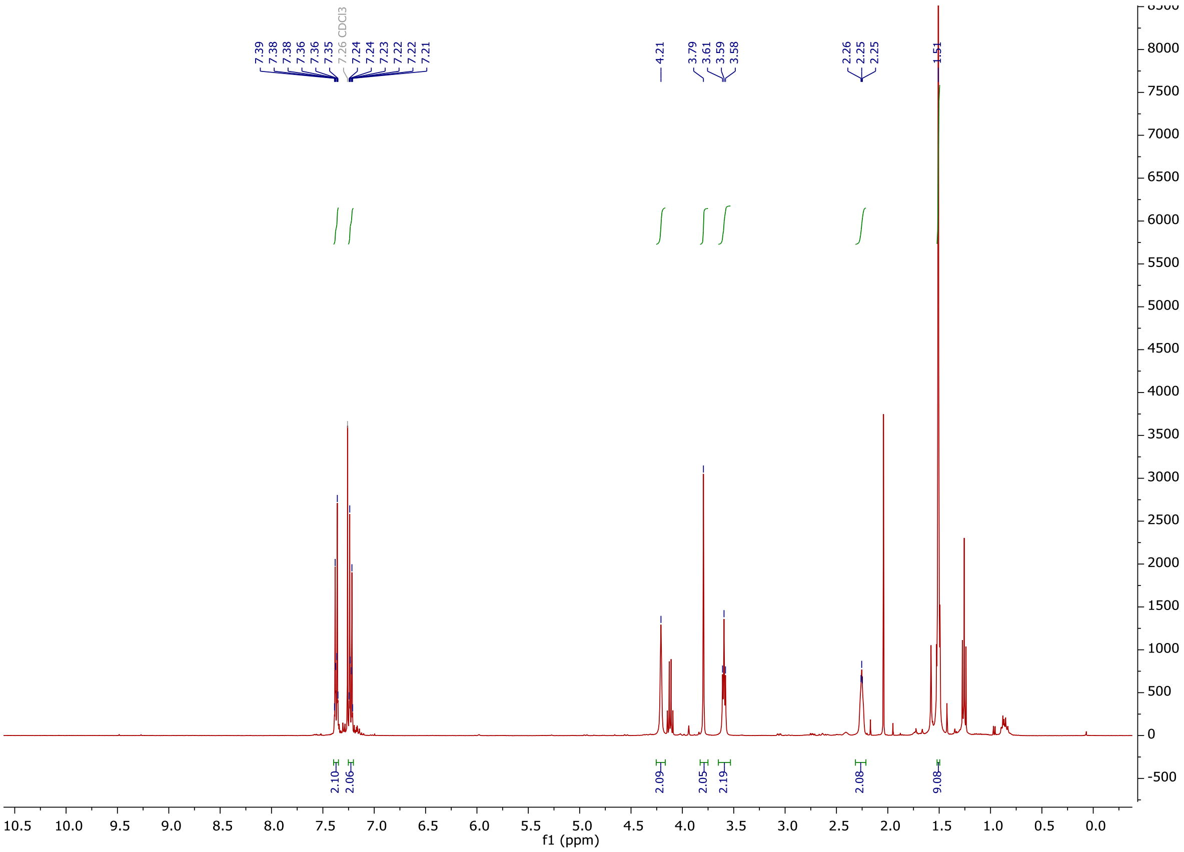

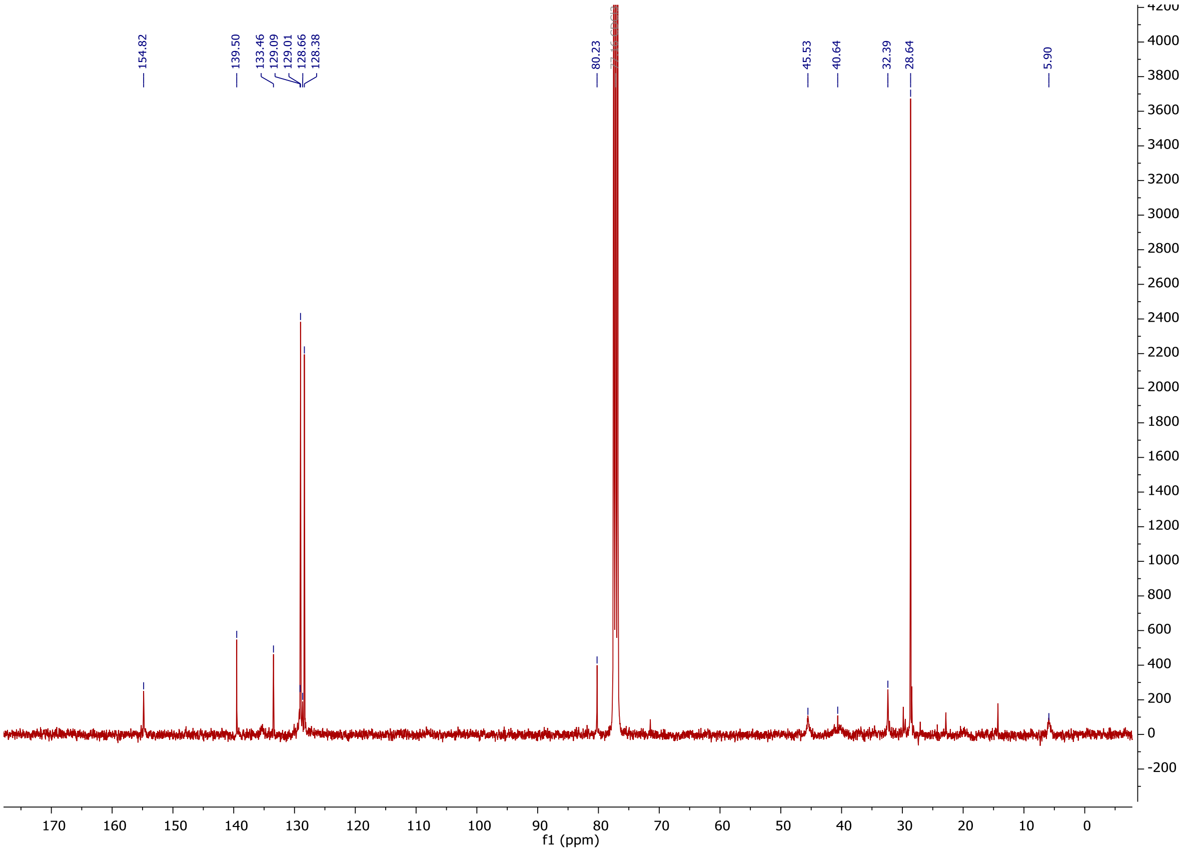

^{1}H and ^{13}C NMR of compound **17**

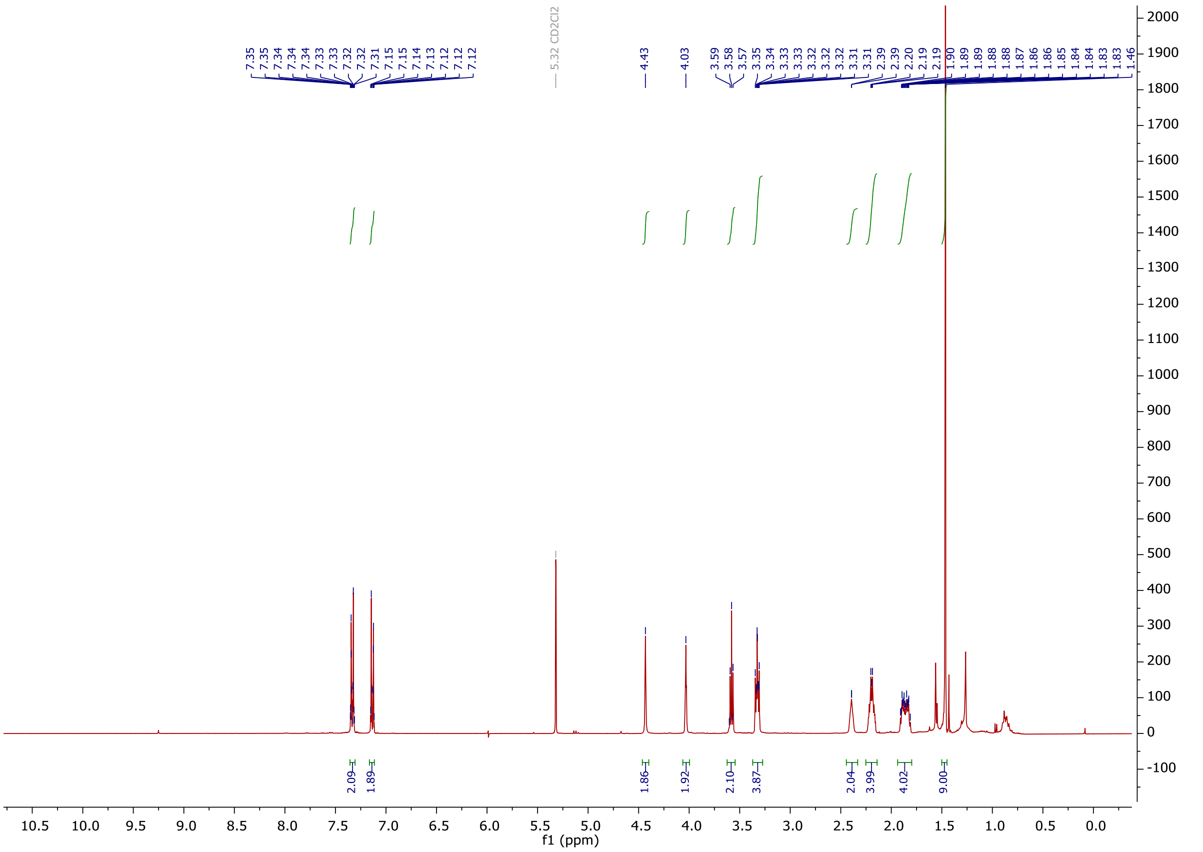

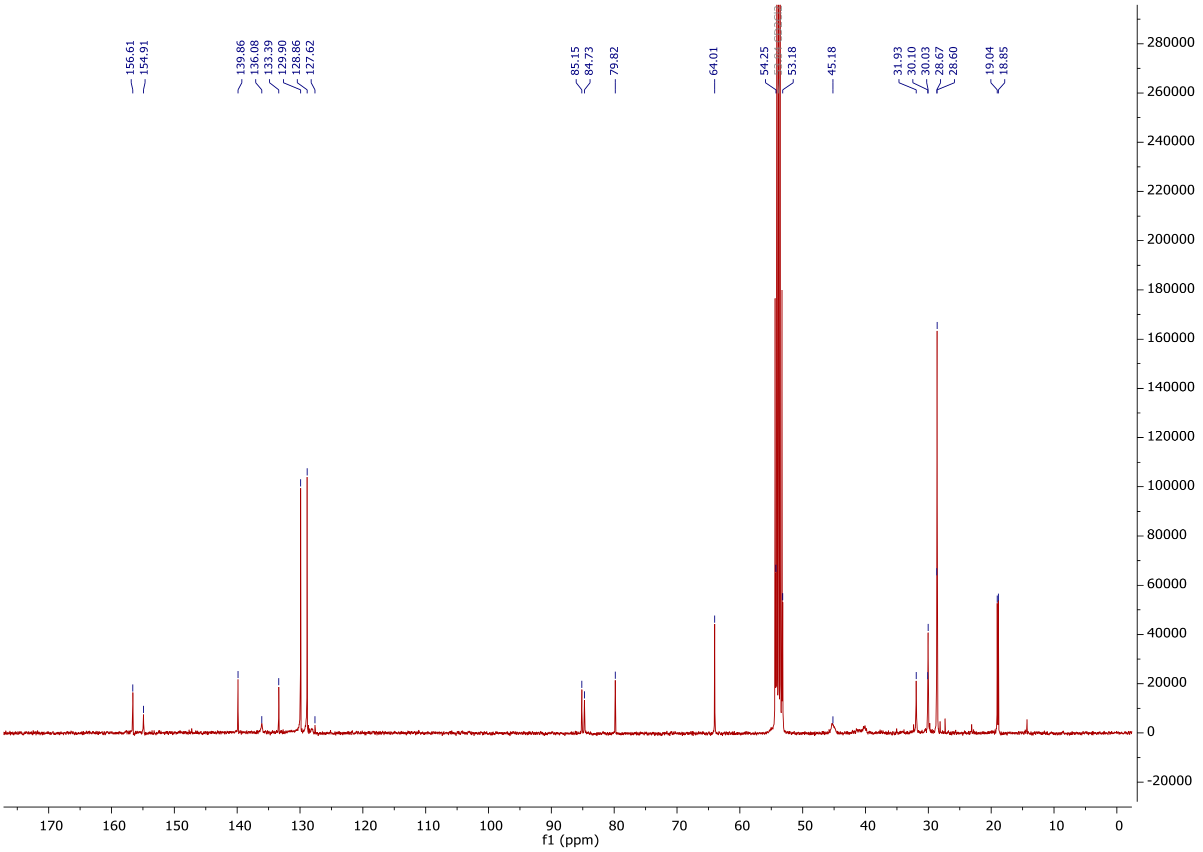

^{1}H and ^{13}C NMR of compound **12d**

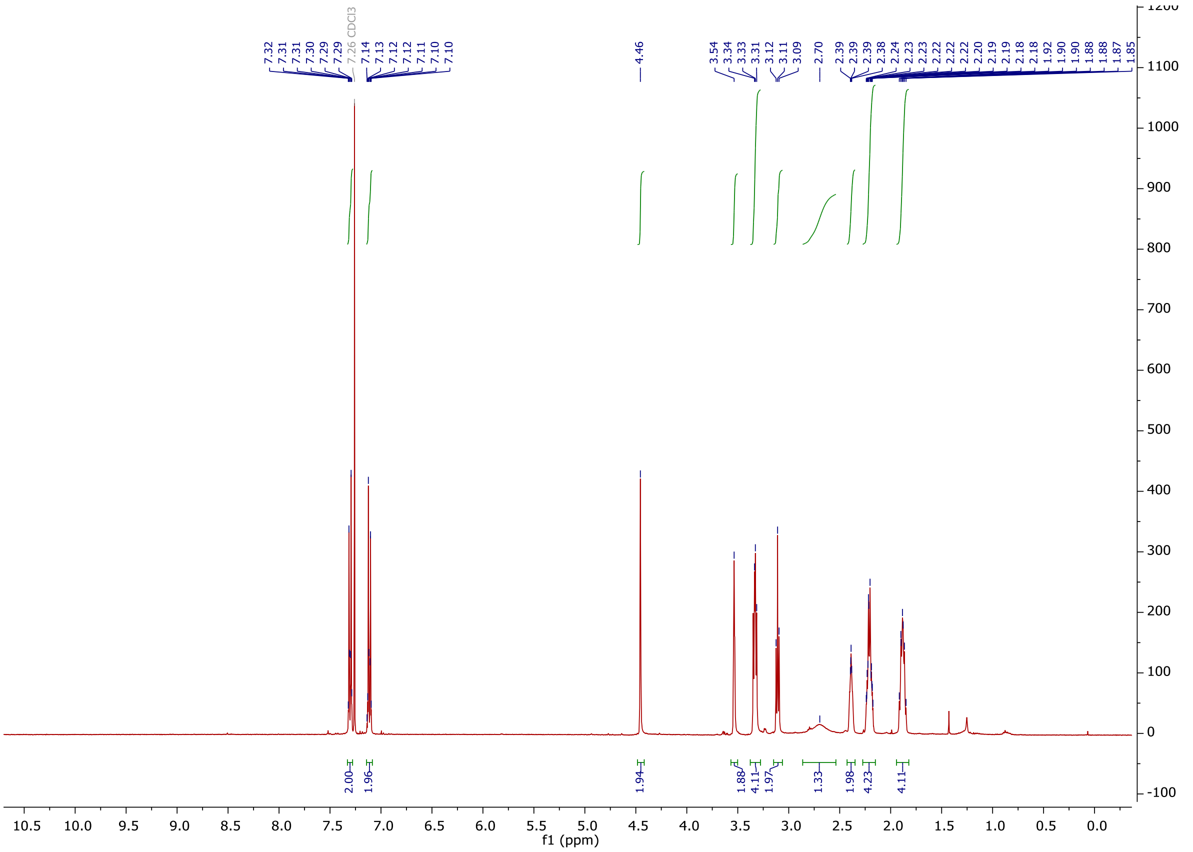

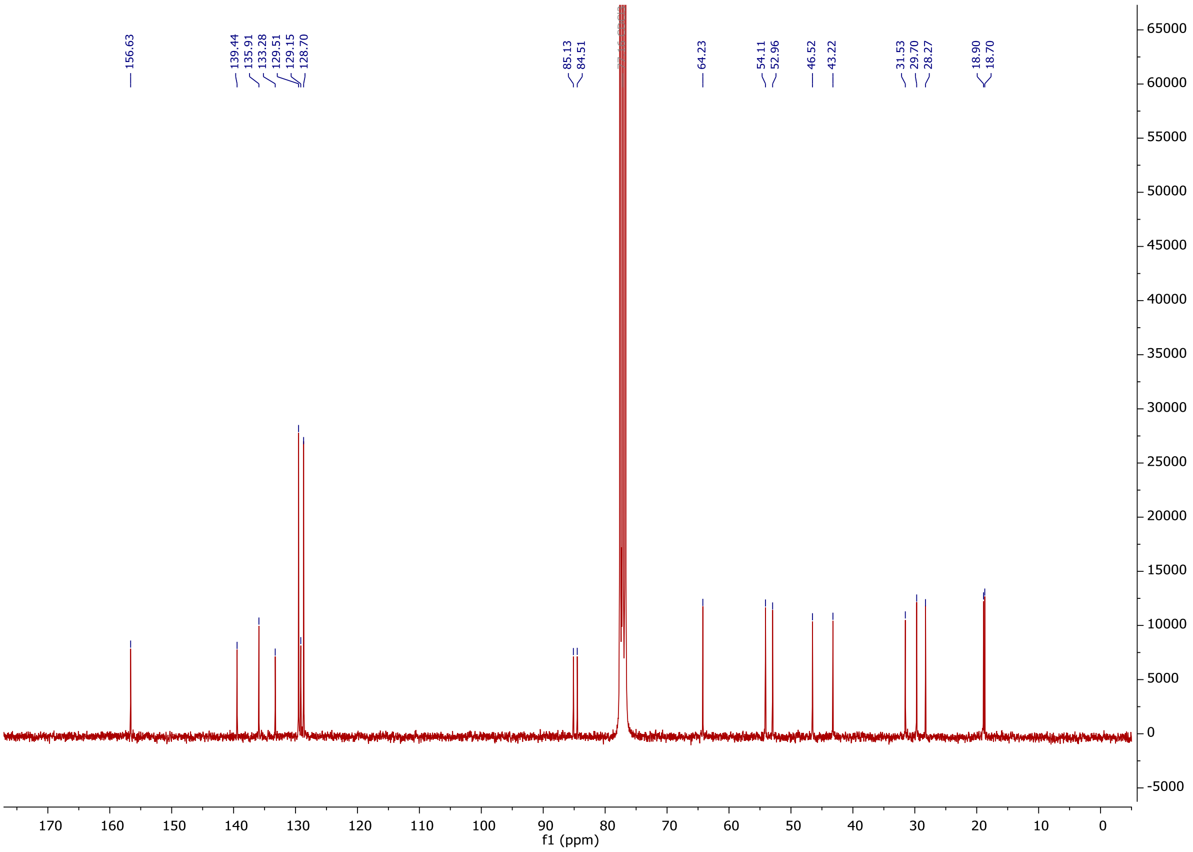

^{1}H and ^{13}C NMR of compound **11d**

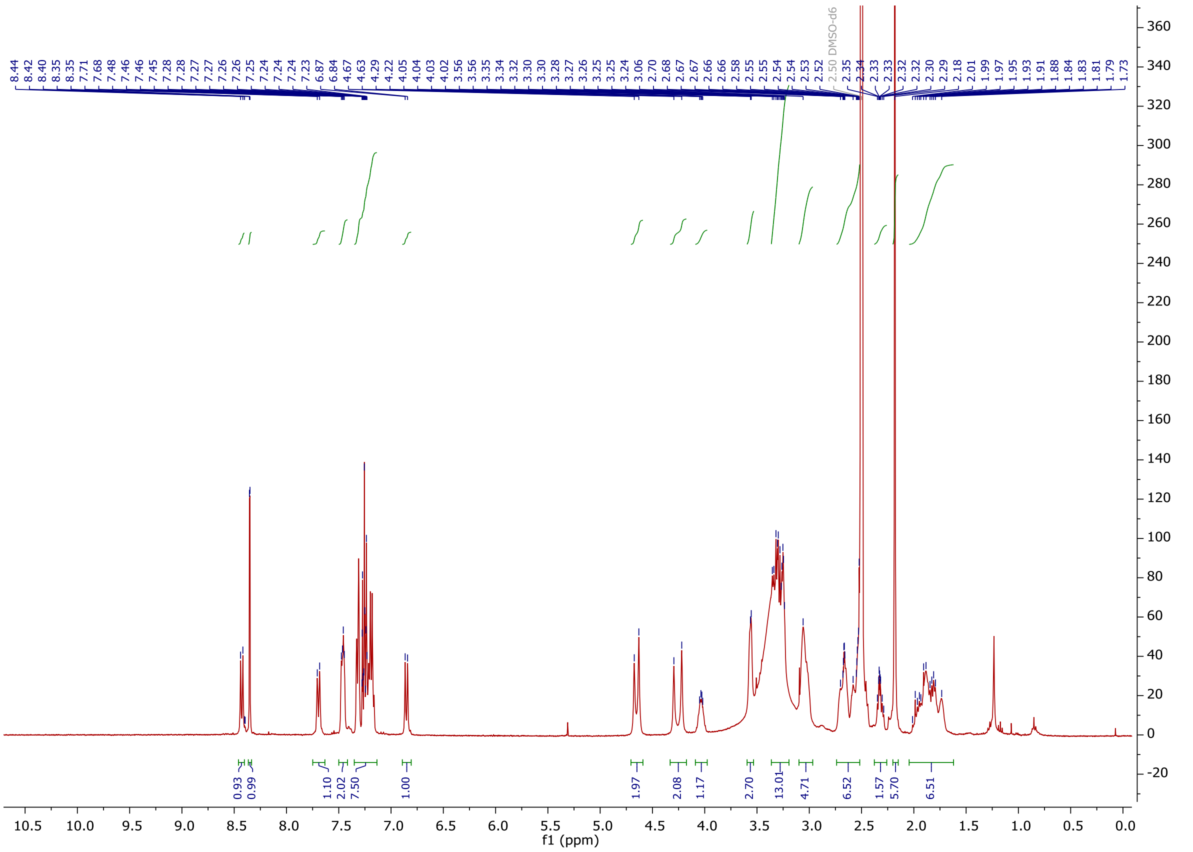

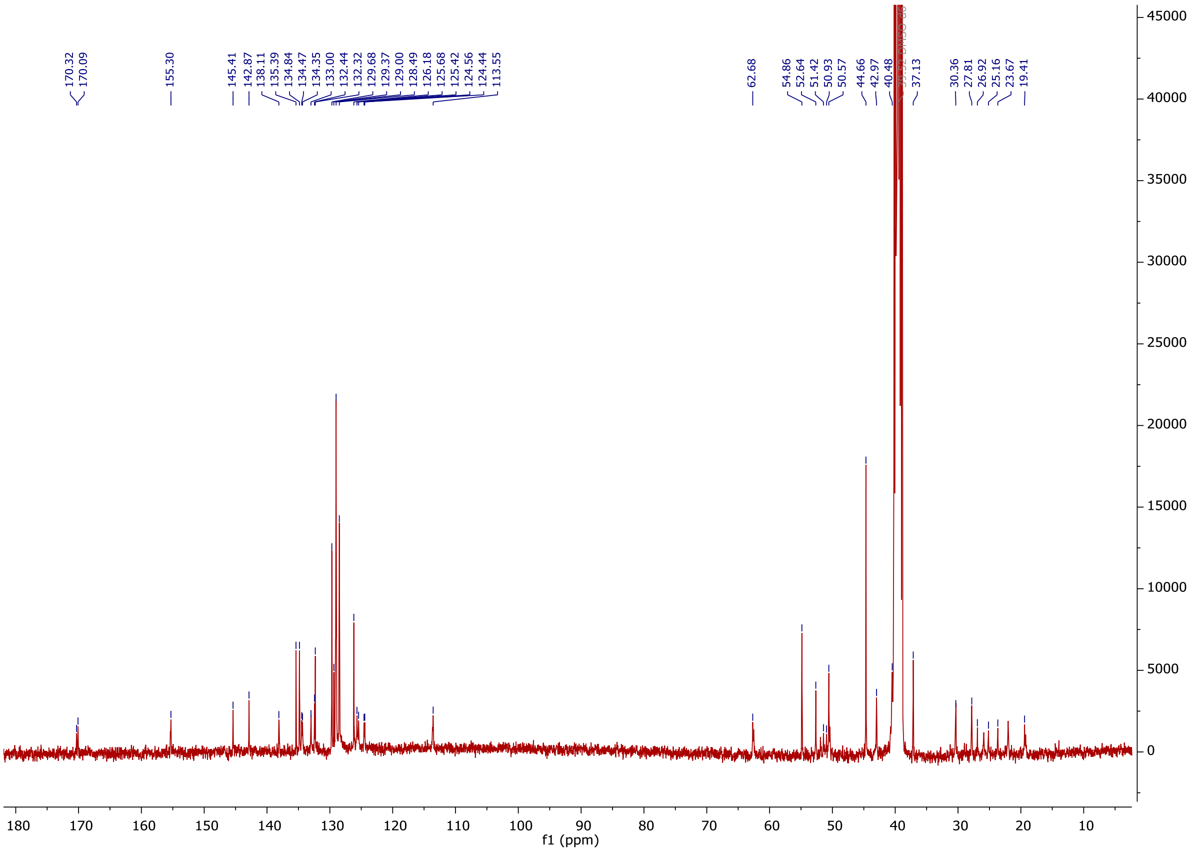

Supporting references

[1] N. M. Garrido, M. Blanco, I. F. Cascon, D. Diez, V. M. Vicente, F. Sanz, J. G. Urones, *Tetrahedron Asymmetry* **2008**, *19*, 2895-2900.

[2] D. Raederstorff, A. Y. L. Shu, J. E. Thompson, C. Djerassi, *J. Org. Chem.* **1987**, *52*, 2337-2346.

[3] D. M. Hodgson, L. A. Robinson, *Chem. Commun.* **1999**, 309-310.

[4] C. Lis, S. Rubner, C. Grost, R. Hoffmann, D. Knappe, T. Berg, *Chem. Eur. J.* **2018**, *24*, 13762-13766.

[5] R. Lacheretz, D. G. Pardo, J. Cossy, *Org. Lett.* **2009**, *11*, 1245-1248.

[6] Y. Zhang, W. Wu, Z. Li, S. Chen, *PCT Int. Appl. WO2017202376A1* **2017**.
